# Genome-wide analysis of putative peroxiredoxin in unicellular and filamentous cyanobacteria

**DOI:** 10.1186/1471-2148-12-220

**Published:** 2012-11-16

**Authors:** Hongli Cui, Yipeng Wang, Yinchu Wang, Song Qin

**Affiliations:** 1 Yantai Institute of Coastal Zone Research, Chinese Academy of Sciences, Chunhui Road, Yantai 264003, People’s Republic of China; 2 University of Chinese Academy of Sciences, Yuquan Road, Beijing 100049, People’s Republic of China

**Keywords:** Peroxiredoxin, Structure, Phylogeny and evolution, Comparative genomics, Cyanobacteria

## Abstract

**Background:**

Cyanobacteria are photoautotrophic prokaryotes with wide variations in genome sizes and ecological habitats. Peroxiredoxin (PRX) is an important protein that plays essential roles in protecting own cells against reactive oxygen species (ROS). PRXs have been identified from mammals, fungi and higher plants. However, knowledge on cyanobacterial PRXs still remains obscure. With the availability of 37 sequenced cyanobacterial genomes, we performed a comprehensive comparative analysis of PRXs and explored their diversity, distribution, domain structure and evolution.

**Results:**

Overall 244 putative *prx* genes were identified, which were abundant in filamentous diazotrophic cyanobacteria, *Acaryochloris marina* MBIC 11017, and unicellular cyanobacteria inhabiting freshwater and hot-springs, while poor in all *Prochlorococcus* and marine *Synechococcus* strains. Among these putative genes, 25 open reading frames (ORFs) encoding hypothetical proteins were identified as *prx* gene family members and the others were already annotated as *prx* genes. All 244 putative PRXs were classified into five major subfamilies (1-Cys, 2-Cys, BCP, PRX5_like, and PRX-like) according to their domain structures. The catalytic motifs of the cyanobacterial PRXs were similar to those of eukaryotic PRXs and highly conserved in all but the PRX-like subfamily. Classical motif (CXXC) of thioredoxin was detected in protein sequences from the PRX-like subfamily. Phylogenetic tree constructed of catalytic domains coincided well with the domain structures of PRXs and the phylogenies based on 16s *rRNA*.

**Conclusions:**

The distribution of genes encoding PRXs in different unicellular and filamentous cyanobacteria especially those sub-families like PRX-like or 1-Cys PRX correlate with the genome size, eco-physiology, and physiological properties of the organisms. Cyanobacterial and eukaryotic PRXs share similar conserved motifs, indicating that cyanobacteria adopt similar catalytic mechanisms as eukaryotes. All cyanobacterial PRX proteins share highly similar structures, implying that these genes may originate from a common ancestor. In this study, a general framework of the sequence-structure-function connections of the PRXs was revealed, which may facilitate functional investigations of PRXs in various organisms.

## Background

Cyanobacteria are among the earliest organism branching groups on earth, dating back 2.5-3.5 billion years, based on the fossil evidences [1]. As a taxonomic unit characterized by the first photosynthetic organisms with an oxygenic type of photosynthesis [2,3], cyanobacteria comprise a large number of species with diverse genome sizes and ecological habitats. Specifically, the genome size varies from 1.6 Mb (*Prochlorococcus* sp. MIT9301) to 9.0 Mb (*Nostoc punctiforme* PCC 73102) and the number of genes ranges from 1,756 (*Prochlorococcus marinus* MED4) to 8,462 (*Acaryochloris marina* MBIC11017) [4-6]. The remarkable variation in genome size indicates their significance in comparative genome research [7]. Cyanobacteria may also be unicellular or filamentous and can be found in almost all the conceivable environments, including marine and freshwater habitats, soil and rocks and extreme environments [8,9]. Unicellular cyanobacteria (*Prochlorococcus* and *Synechococcus*), which can inhabit ocean and possess the smallest genome size, is responsible for significant biomass and primary production in the marine biosphere [10]. Three unicellular cyanobacteria (*Thermosynechococcus elongatus* BP-1, *Synechococcus* sp. JA-2-3B’a (2–13) and *Synechococcus* sp. JA-3-3Ab) were isolated from hot-springs. Other unicellular species have larger genome sizes, including water bloom forming cyanobacteria (*Synechocystis* sp. PCC 6803 and *Microcystis aeruginosa* NIES-843), a thylakoids-absence cyanobacterium (*Gloeobacter* sp. PCC 7421), a nitrogen-fixing cyanobacterium (*Cyanothece* sp. ATCC 51142), and an animal-cyanobacterial symbionsis (*Acaryochloris marina* MBIC11017) [11] . The diazotrophic filamentous cyanobacteria have the largest genome sizes and include strains isolated from fresh water (*Anabaena* PCC7120, *Anabaena variabilis* ATCC 29413 and *Arthrospira. platensis* NIES-39), from a plant-cyanobacterial symbionsis (*Nostoc punctiforme* PCC29133), and from tropical and subtropical oceans (*Trichodesmium erythraeum* IMS101). In addition, the phylogeny of sequenced cyanobacterial organisms has been reported in previous studies [7,12,13].

Similar to heterotrophic organisms, cyanobacteria need to manage the ROS generated by oxygen reduction; however, they must also regulate ROS produced during photosynthetic electron transport [14,15]. Indeed, cyanobacteria constantly produce oxygen under illumination, which makes it crucial for them to prevent electron escape from normal electron transfer pathways to oxygen and ROS production [14]. Living organisms have developed various antioxidant defense mechanisms to protect themselves against ROS damage, including enzymatic (catalases, superoxide dismutases (SOD) and peroxidases), and non-enzymatic (glutathione, peroxiredoxins, vitamin A, C, E, and carotenoids) pathway [14,16,17].

The main factors involved in the cyanobacterial ROS-scavenging system are low molecular mass antioxidants (peroxiredoxins, ferredoxin, glutathione, *beta*-carotenoids, and tocopherol) and enzymes of the Halliwell-Asada cycle in combination with peroxisomal catalase and superoxide dismutase [15,18-20]. A catalase-peroxidase was purified and characterized from *Synechococcus elongatus* PCC 7942 [21]. Additionally, the *katG* gene (encoding bi-functional catalase-peroxidase) was cloned and characterized from *Synechocystis* sp. strain PCC 6803 [22-24]. Recently, several studies about the catalytic mechanisms of the bi-functional catalase KatG from *Synechocystis* PCC 6803 have been published (for a review, see [25]). Genome sequence analysis of 64 cyanobacterial SODs indicated that the Cu/Zn form of SOD is rare among all cyanobacteria. Specifically, the marine unicellular *Prochlorococcus* species only possess Ni SOD, whereas other unicellular strains possess Fe SOD and Ni SOD or Fe SOD and Mn SOD [26].

Peroxiredoxins (PRXs) comprise an important antioxidant protein family with the ability to detoxify peroxide and the *prx* gene has recently been identified from higher plants [27]. Members of the PRX family are thiol-specific reductases or peroxidases [28]. PRXs exist as the form of multiple isoforms and catalyze the reduction of a broad range of different peroxides, including hydrogen peroxide, alkyl hydroperoxides and peroxinitrite [29,30]. The existence of different PRX family members has already been recorded in a wide variety of organisms ranging from archaea to mammals [31]. Six different sub-classes of PRXs, PRX I-IV (2-Cys PRX), PRX V (Type II PRX) and PRX VI (1-Cys PRX), have been identified from mammalian systems [32]. However, only four PRX sub-classes (1-Cys PRX, 2-Cys PRX, Type II PRX and PRX Q) have been reported in higher plants systems [29]. Analyses of the genome sequence of *Synechocystis* sp. PCC 6803 revealed the presence of five genes encoding peroxiredoxins 2-Cys PRX (*sll0755*), 1-Cys PRX (*sll1198*), two PRX Q (*sll0221* and *slr0242*) and one Type II PRX (*sll1621*) [19,28,33]. Analyses of the genome sequence of *Synechococcus elongatus* PCC 7942 led to identification of six putative *prx* genes including one 1-Cys PRX, one 2-Cys PRX and four PRX Q [34]. Now that with the complete and partial of genomes from several cyanobacterial species, genome-wide identification and analysis of PRXs in cyanobacteria becomes possible.

Recently, 37 genomes of unicellular and filamentous cyanobacteria became available, which has facilitated the cyanobacterial systemic analysis of carotenoid cleavage dioxygenases [35], the metacaspases family [7], fatty acid desaturases [36], serine/threonine protein kinases [12], restriction modification systems [37], and carotenoids biosynthesis [38]. Comparative genomic investigations of cyanobacterial superoxide dismutases have also been conducted [26]. In this study, we selected 11 previously characterized PRXs from *Synechocystis* sp. PCC 6803 and *Synechococcus elongatus* PCC 7942 to search for cyanobacterial PRXs at the genome level. A BLASTp-plus-HMMsearch-phylogeny reconstruction approach was employed to analyze PRXs, focusing on their classification, distribution, structure, phylogeny and evolution. A better understanding of cyanobacterial PRXs can help us to understand the antioxidant mechanisms of cyanobacteria.

## Results

### Identification of open reading frames encoding PRX proteins

**Figure 1:**
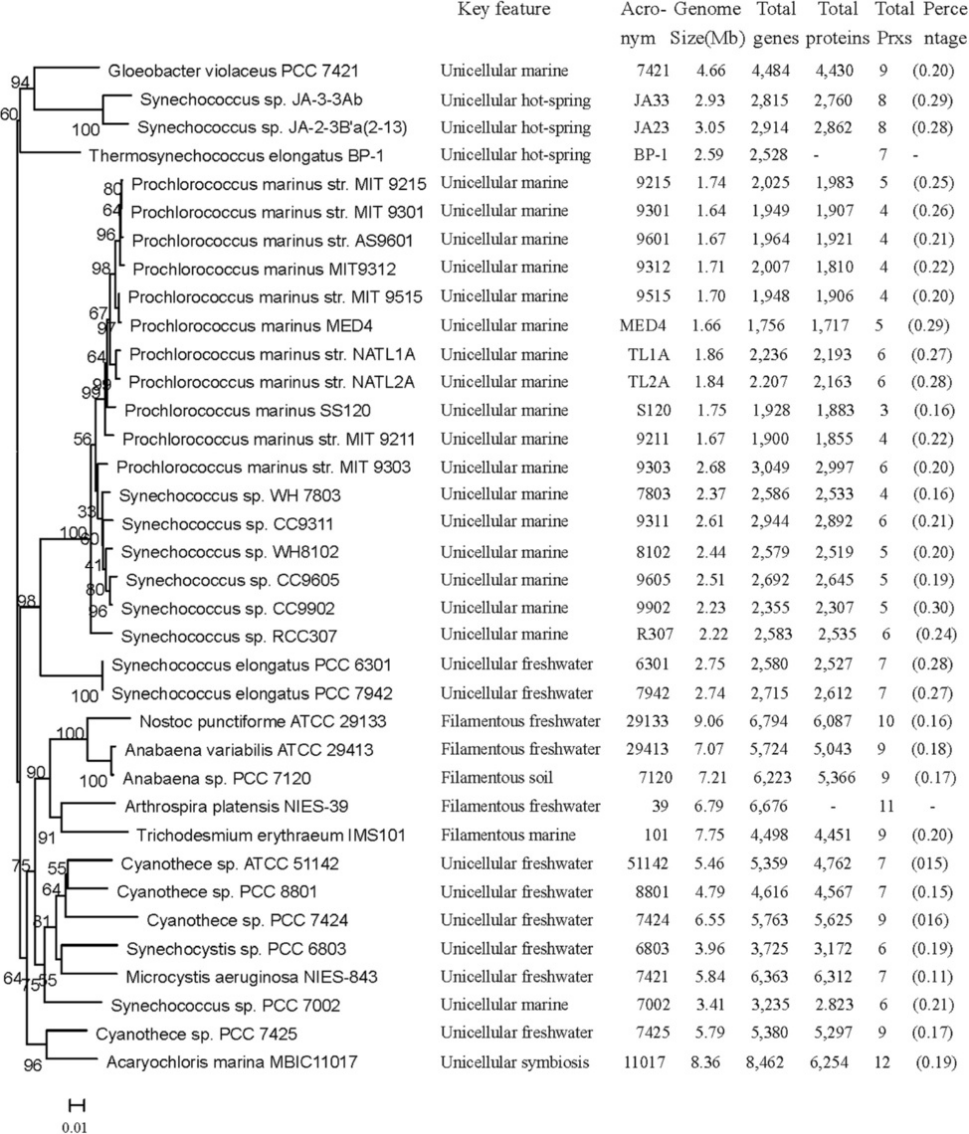
**Phylogenetic tree of the sequenced cyanobacterial strains and *prx *information.** A Maximum likelihood tree for 36 sequenced cyanobacteria constructed based on 16s rRNA was reconstructed as described in the Methods [35]. The General Time Reversible (GTR) substitution model was selected assuming an estimated proportion of invariant sites and four gamma-distributed rate categories to account for rate heterogeneity across sites. The reliability on internal branches was assessed using the bootstrapping method (400 bootstrap replicates). Percentages in brackets represent total PRXs as a percentage of the total proteins. *Prochlorococcus marinus* MIT 9313 are absent from this tree because they are partial genomes and have no complete *rRNA* genes.

A total of 37 complete and partial cyanobacterial genomes were downloaded from the JGI genome portal [39] or Cyanobase [40] and used for this analysis. The information and phylogeny of 37 sequenced cyanobacterial strains were listed in Figure 1. The BLAST (BLASTp and tBLASTn) and HMM (hmmsearch) programs were used to search for proteins similar to confirmed cyanobacterial PRXs in each cyanobacterial genome. Pfam and SMART analysis using the derived sequences were then carried out to eliminate false positives. Among the 254 investigated proteins, ten that were originally annotated as Trx (NIES39_D06120, P9215_11961, Syncc9605_1945, Syncc9902_0720, and SYNW0724), Trx like protein (9301_02651), putative SOD (Syncc9902_0982), probable BCP (BP-1_0473), HP (7421_3157) and Trx domain 2 (Syncc9902_0354) respectively, were found to lack the important catalytic domains of typical PRX upon Pfam and SMART analysis and thus excluded from further consideration. As a result, a total of 244 proteins were considered in this study and an additional table file shows this in more detail [see Additional file 1, Table S1 and S2, among which 79 were originally annotated as AhpC/TSA or AhpC/TSA family members, 66 were originally annotated as BCPs (putative BCP or BCP homolog), 25 were originally annotated as peroxidases and 25 were originally annotated as peroxiredoxins. The remaining 49 proteins were accepted as PRX family members for this study, including 12 proteins annotated by other additional domains (such as 1-Cys, 2-Cys, TSPA, glutaredoxin-family domain protein and rehydin), 25 proteins annotated as hypothetical proteins, 8 proteins annotated as redoxins, and 4 proteins annotated as twin-arginine translocation pathway proteins.

### The distribution of *prx* genes encoding PRX proteins

Amid diverse cyanobacterial genomes, the number of *prx* genes varies from 3 to 12 and the percentage of PRXs in the total proteins ranges from 0.11-0.30% (Figure 1). Among all unicellular cyanobacteria, symbiont *Acaryochloris marina* MBIC 11017 possesses 12 *prx*s, which is much higher than other species. However, the percentage of PRXs within the total proteins of this organism was only 0.19%, which is not the highest among unicellular cyanobacteria. The low ratio may be a result of the large genome of *Acaryochloris marina* MBIC 11017. Within marine unicellular cyanobacteria, the thylakoids-lacking cyanobacterium *Gloeobacter* sp. PCC 7421 possesses 9 *prxs*, which is much higher than others. Only three *prx* genes were found in *Prochlorococcus marinus* SS120, while four to six *prx* genes were found in other *Prochlorococcus marinus* strains and all marine *Synechococcus* strains, including WH 7803/8102, CC 9311/9605/9902, RCC 307, and PCC 7002. The percentage of PRXs within the total proteins was approximately 0.20% in the *Prochlorococcus marinus* strains and marine *Synechococcus* strains. Three *Synechococcus* strains inhabiting hot springs (BP-1, JA-2-3B’a(2–3),and JA-3-3Ab) and two freshwater *Synechococcus elongatus* strains (PCC 6301 and PCC 7942) were found to contain eight and seven *prx* genes, respectively, and these had similar percentages of PRXs in the total proteins (0.27-0.29%). All *Cyanothece* strains were found to contain seven (ATCC 51142 and PCC 8801) or nine (PCC 7424 and PCC 7425) *prx* genes, and the percentages of PRXs within the total proteins were 0.15%-0.17% for these cyanobacteria. The water-blooming cyanobacterium *Microcystis aeruginosa* NIES-843 was found to contain seven *prx* genes and the percentage of PRXs (0.11%) was the lowest among all investigated cyanobacteria. Six *prx* genes were found in *Synechocystis* sp. PCC 6803.

Compared with unicellular cyanobacteria, filamentous diazotrophic cyanobacteria possess more *prx* genes (10 for *Nostoc punctiforme* PCC 29133, 9 for *Anabaena variabilis* ATCC 29413, 9 for *Anabaena* sp. PCC 7120, 9 for *Trichodesmium erythraeum* IMS 101, and 11 for *Arthrospira platensis* NIES-39). However, the percentages of PRXs in the total proteins of these cyanobacteria were only 0.16%-0.18%, which was lower than those from marine unicellular cyanobacteria.

**Figure 2:**
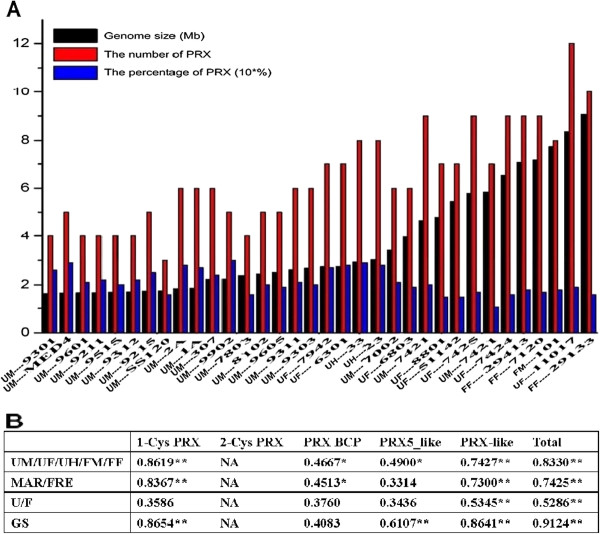
**Correlation between the distribution of *prx *and the eco-physiological properties and genome sizes of cyanobacteria.** The number of *prx* in each species was determined based on the genes encoding PRX in the genomes. Percentages represent total *prx* as a percentage of total proteins. Strain names and the eco-physiological properties are as in Figure 1 and Additional file 1. Statistical analyses on the relationship between the distribution of genes encoding PRXs from different sub-families and the properties of 37 cyanobacterial organisms were performed using the Spearman Rank Correlation test (R), respectively. For the test of the distribution of *prx* and the genome size, cellular morphology, habitat and eco-physiological properties of 37 cyanobacterial organisms, the *X*_*i*_ is GS, U/E, MAR/FRE or UM/UF/UH/FM/FF and the *Y*_*i*_ is the total number of *prx* or the number of *prx* belonging to different sub-families and the in each cyanobacterial organisms. Note: *X*, the independent variable; *Y*, the dependent variable; GS, genome size (from small to large); U/F, unicellular or filamentous; MAR /FRE, marine or freshwater; UM/UF/UH/FM/FF, unicellular marine, unicellular freshwater, unicellular hot-spring, filamentous marine or filamentous freshwater. Spearman Rank test indicated that the distribution of some PRX family such as PRX-like or 1-Cys PRX correlate well with the eco-physiological properties and genome sizes of cyanobacteria (“*”, *p*-value <=0.01; “**”, p-value <=0.001).

The number of *prx* gene is different from various habitat niches and genome sizes (Figure 2A). Unicellular cyanobacteria habiting marine contain the minimum amount of *prx* than those from freshwater and hot-springs. A similar phenomenon occurred in the filamentous cyanobacteria from marine and freshwater. The number of *prx* gene is increasing along with the increasing of the genome size of different cyanobacteria (Figure 2A). However, regardless of the habitat niches and cellular morphology, the percentage of PRX in the total proteins decreased along with the increased genome sizes. It is evident from these findings that filamentous diazotrophic cyanobacteria contain more *prx* genes than unicellular species, whereas the number of *prx* genes provides insufficient representation after allowing for their larger genomes. Moreover, in order to study the relationship between gene distribution and properties of the organisms, Spearman Rank Correlation test (R) was carried out and specific results were summarized in Figure 2B. Based on the summary on Figure 2B, the correlations between different properties and gene distribution were different. The total number of *prx* genes and genes encoding PRX from 1-Cys and PRX-like subfamilies share close correlations with genome size and eco-physiology properties of the organisms, while the other did not.

### Structures and functions

**Figure 3:**
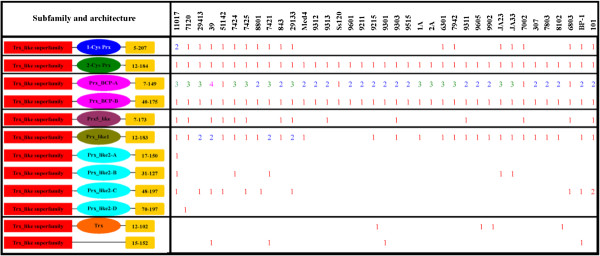
**Schematic representation and distribution of putative cyanobacterial PRX.** Fused domains forming a single polypeptide chain are connected by a horizontal line. The red rectangles represent the Trx_like superfamily. Elliptical shadows with different colours represent different PRX subfamilies. Yellow rectangles represent the length of overlap. Strain and domain names are as in Figure 1 and Additional file 1, respectively. Figures are not drawn to scale.

Pfam and SMART domain analysis could not distinguish subfamilies among the cyanobacterial PRXs. Moreover, most of the proteins originally annotated as AhpC/TSA, BCP, and peroxiredoxin were not classified into distinct subfamilies. Fortunately, based on structural characteristics acquired from the CDD domain (Conserved Domain Database) analysis, the identified cyanobacterial PRXs could be classified into five major subfamilies: 1-Cys PRX, 2-Cys PRX, PRX BCP, PRX5_like, and PRX-like (Figure 3).

Cyanobacterial PRX subfamily I (1-Cys PRX) includes 20 (8.19%) PRXs with less than 200 amino acid residues and is considered to possess the basic active sites in 26–50 residues. Genes encoding PRX proteins from this subfamily are present in five filamentous cyanobacteria (*Anabaena* sp. PCC 7120, *Anabaena variabilis* ATCC 29413, *Arthrospira platensis* NIES-39, *Nostoc punctiforme* ATCC 29133 and *Trichodesmium erythraeum* IMS101), eight unicellular cyanobacteria inhabiting freshwater (*Synechocystis* sp. PCC 6803, *Microcystis aeruginosa* NIES-843, *Synechococcus elongatus* PCC 6301/7942, *Cyanothece* sp. PCC 8801/7424/7425 and *Cyanothece* sp. ATCC 51142), and three unicellular cyanobacteria inhabiting hot-springs (*Thermosynechococcus elongatus* BP-1, *Synechococcus* sp. JA-3-3Ab and *Synechococcus* sp. JA-2-3B'a(2–13)). It is interesting that 1-Cys PRX coding genes are a single gene in each cyanobacterial strain, whereas two genes encoding this PRX are found in *Acaryochloris marina* MBIC11017. However, genes encoding PRX from this subfamily are absent from all marine unicellular cyanobacteria except for *Gloeobacter violaceus* PCC 7421 and *Synechococcus* PCC 7002.

Subfamily II (2-Cys PRX) is the largest class of PRXs and characterized by two conserved redox-active cysteines, a peroxidatic cysteine (generally near residues 51–73) and a resolving cysteine (near residues 183–188). Subfamily II contains 37 (15.16%) proteins with less than 210 amino acid residues. Every one of all cyanobacterial organisms possess a single gene coding 2-Cys PRX respectively, suggesting that these genes are highly conserved throughout the evolutionary history.

Subfamily III (PRX BCP), bacterioferritin comigratory protein (BCP), was named based on its electrophoretic mobility before its function was known. BCP contains the peroxidatic cysteine and a putative resolving cysteine near the N-terminal. This subfamily was further divided into two types. Type a (PRX BCP-A) contains 85 (34.84%) proteins with less than 170 amino acid residues and was considered to possess the peroxidatic cysteinal basic structure in residues 44–61. There are several paralogous genes encoding PRXs from this type, which are widely distributed among almost all cyanobacteria except for *Cyanothece* sp. ATCC 51142, *Prochlorococcus marinus* SS120, *Synechococcus* PCC 7002, and *Synechocystis* sp. PCC 6803. Type b (PRX BCP-B) comprises 37 (15.16%) proteins with less than 200 amino acid residues and is considered to possess the peroxidatic cysteinal basic structure in residues 75–93. Compared to the paralogous genes encoding PRX BCP-A, all 37 cyanobacterial organisms possess a single gene encoding PRX BCP-B. It is apparent that the position of the peroxidatic cysteinal basic structure can be applied to distinguish these two types of PRX BCP proteins, which comprise the majority (50.00%) of cyanobacterial PRXs.

The fourth subfamily of PRX is PRX5-like, a homodimeric trx peroxidase, is widely expressed in mitochondria, peroxisomes and cytosol. This subfamily comprises 15 (6.14%) proteins with less than 190 amino acid residues and is considered to possess a peroxidatic cysteinal basic structure in residues 46–63. These 15 (6.14%) proteins are found in *Acaryochloris marina* MBIC11017, *Anabaena* sp. PCC 7120, *Cyanothece* sp. PCC 7424/7425, *Cyanothece* sp. ATCC 51142, *Nostoc punctiforme* ATCC 29133, *Microcystis aeruginosa* NIES-843, *Prochlorococcus marinus* 9313/9303/9311, *Synechococcus* PCC 7002, *Synechocystis* sp. PCC 6803, *Arthrospira platensis* NIES-39, and *Trichodesmium erythraeum* IMS101. *Prx* genes encoding PRX proteins from this subfamily are only detected in a few cyanobacteria, rather than all cyanbacterial strains, implying that they may exist in a species-specific fashion.

The last subfamily of PRX is PRX-like, members of which were originally annotated as hypothetical proteins. The protein sequences from this subfamily show similarity to PRXs and contain the conserved CXXC motif. We speculated that one specific cysteine in the motif corresponds to the peroxidatic cysteine of PRX. However, these proteins do not contain the other two residues of the typical catalytic triad of PRX. This subfamily was further divided into two types. Type c (PRX_like1) possesses the CXXC motif (near residues 52–65) in the N-terminal, as well as the putative typical catalytic triad of PRX in the C-terminal (near residues 134–140). The 32 (13.11%) proteins from this type were found to be distributed among all filamentous cyanobacteria and unicellular cyanobacteria living in marine (*Synechococcus*), freshwater (except for *Synechocystis* sp. PCC 6803), and hot-springs, whereas they were absent from all *Prochlorococcus marinus* (except for 9215 and 9301). Type d (PRX_like2) possesses the CXXC motif (near residues 64–77) in the N-terminal and contains 17 proteins (6.96%) that are distributed in all five filamentous cyanobacteria (*Anabaena* sp. PCC 7120, *Anabaena variabilis* ATCC 29413, *Arthrospira platensis* NIES-39, *Nostoc punctiforme* ATCC 29133, and *Trichodesmium erythraeum* IMS101), three hot-springs inhabitant cyanobacteria (*Thermosynechococcus elongatus* BP-1, *Synechococcus* sp. JA-3-3Ab and *Synechococcus* sp. JA-2-3B'a(2–13)), and the freshwater unicellular *Cyanothece* group.

### Phylogenetic analysis

**Figure 4:**
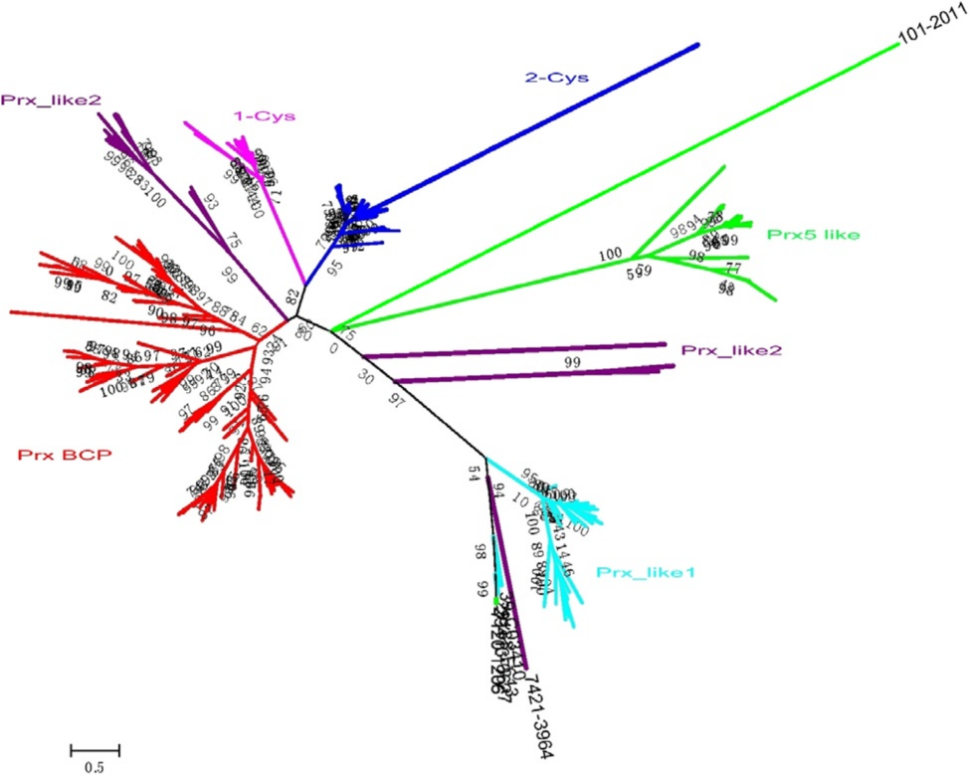
**Phylogenetic trees of the total Prxs.** A Maximum likelihood tree of 260 PRX sequences from cyanobacteria, higher plants, and *Metazoa* was constructed as described in the Methods. The Le and Gascuel evolutionary mode (LG) was selected assuming an estimated proportion of invariant sites and four gamma-distributed rate categories to account for rate heterogeneity across sites. Reliability of internal branches was assessed using the bootstrapping method (400 bootstrap replicates). PRXs from distinct subfamilies are indicated by different colours.

To elucidate the evolutionary histories between species and cyanobacterial *prx* genes, the translated protein sequences of these genes and previously proven PRX proteins (Table 1) were applied to construct the phylogenetic tree. Six major clades were observed in the phylogenetic tree in general (Figure 4). PRXs from 1-Cys, 2-Cys, PRX BCP and most of the PRX-like2 subfamilies belonged to the first monophyletic (BS: 80%) group. The second monophyletic (BS: 75%) group contains members of the PRX5_like and PRX-like subfamily with all PRX-like1 and some PRX-like2, which cluster separately according to their domains, respectively. According to the results of the phylogenetic tree (Figure 4), most members (except proteins 7120_1206 and 11107_5336) of different subfamilies are consistent with the classification (Figure 3 and Additional file 1, Table S1) based on CDD domain analysis, which indicates that cyanobacterial PRXs cluster strictly according to their structural characteristics. In addition, the PRXs generally cluster within each subfamily according to the phylogeny of the species.

**Table 1:** List of organisms and PRX protein sequences analyzed in this study (except for the sequences from cyanobacterial genomes)

**Species**	**Accession No.**	**Length**	**Protein**
*Arabidopsis thaliana*	CAA72804.1	216	1-cys
*Arabidopsis thaliana*	sp|Q96291.2	266	2-Cys Prx A
*Arabidopsis thaliana*	sp|Q9C5R8.3	273	2-Cys Prx B
*Arabidopsis thaliana*	AEE77109.1	216	Prx Q
*Arabidopsis thaliana*	AAM65848.1	162	Prx -2B
*Arabidopsis thaliana*	AEE74337.1	201	Prx -2F
*Arabidopsis thaliana*	NP_176774.1	553	Prx -2A
*Arabidopsis thaliana*	sp|O22711.2	162	Prx -2D
*Arabidopsis thaliana*	sp|Q949U7.2	234	Prx -2E
*Arabidopsis thaliana*	sp|Q9SRZ4.1	162	Prx -2C
*Homo sapiens*	AAA50464	199	Prx I (2-Cys)
*Homo sapiens*	AAA50465	198	Prx II (2-Cys)
*Homo sapiens*	BAA08389	256	Prx III (2-Cys)
*Homo sapiens*	AAB95175	271	Prx IV (2-Cys)
*Homo sapiens*	AAF03750	214	Prx V (atypical 2-Cys)
*Homo sapiens*	BAA03496	224	Prx VI (1-Cys)

Several interesting results emerged from further analysis of the phylogeny of cyanobacterial PRXs. All *prx* genes encoding PRX BCP formed three major clades and an additional figure file shows this in more detail [see Additional file 2, Figure S1]. Several paralogous genes encoding PRX BCP-A compose a monophyletic (BS: 90%) group. As expected, the PRX Q from *Arabidopsis thaliana* [GenBank: AEE77109.1] clusters with the PRX BCP subfamily, suggesting a cyanobacterial-origin of this gene in higher plants. Meanwhile, genes encoding PRX BCP-B proteins form a monophyletic (BS: 89%) group. Most genes encoding PRX BCP are paralogous based on their close evolutionary relationships, suggesting that they share common ancestors and may have been produced by recent gene duplication. It is obvious that PRXs BCP from *Gloeobacter violaceus* PCC7421 (gll_0506), *Synechococcus* sp. JA-2-3B'a(2–13) (CYB_1376), *Synechococcus* sp. JA-3-3Ab (CYA_2305), and *Arthrospira platensis* NIES-39 (NIES39_E02230) formed a separate cluster, respectively, indicating obvious species-specific duplication events in these strains. The 2-Cys PRX from higher plants build a monophyletic group (BS: 88%) with all the cyanobacterial 2-Cys PRXs except for 7421_3158, suggesting a common ancestor and an additional figure file shows this in more detail [see Additional file 3, Figure S2]. Surprisingly, more than one *prx* genes coding 2-Cys were discovered from *Homo sapiens* (four genes) and higher plants (two genes), indicating recent gene duplication occur in linage-specific fashion. All *prx* genes encoding PRX-like were clustered into two major clades and an additional figure file shows this in more detail [see Additional file 4, Figure S3]. Members belonging to PRX-like1 comprise a monophyletic (BS: 84%) group. Members from PRX-like2 build a monophyletic group (BS: 99%). It is interesting that one protein (*Anabaena* sp. PCC 7120: 7120_1206) belonged to Prx5_like subfamily build a monophyletic group (BS: 96%) with three *prx* encoding Prx-like1, suggesting that a natural recombination, a lateral gene transfer, or convergent evolution took place. In the subfamily PRX5_like and an additional figure file shows this in more detail [see Additional file 5, Figure S4], the PRX5_like subfamily also includes six type II PRXs (type 2A/2B/2C/2D/2E/2F) from *Arabidopsis thaliana* [GenBank: NP_176774.1, AAM65848.1, sp|Q9SRZ4.1, sp|O22711.2, sp|Q949U7.2, and AEE74337.1] and the typical 2-Cys PRX from *Metazoa* [GenBank: AAF03750]. Surprisingly, six *prx* genes encoding PRX from higher plants clustered with one protein from *Metazoa* but the cyanobacterial PRX, implying that a non-cyanobacterial origin of this gene encoding PRX typeII proteins in higher plants. Additionally, 1-Cys PRXs from *Arabidopsis thaliana* and *Homo sapiens* formed one clade and build sister group with all cyanobacterial 1-Cys PRXs, indicating a non-cyanobacterial origin of 1-Cys *prx* genes in higher plants and an additional figure file shows this in more detail [see Additional file 6, Figure S5].

### Conserved domain features

**Figure 5:**
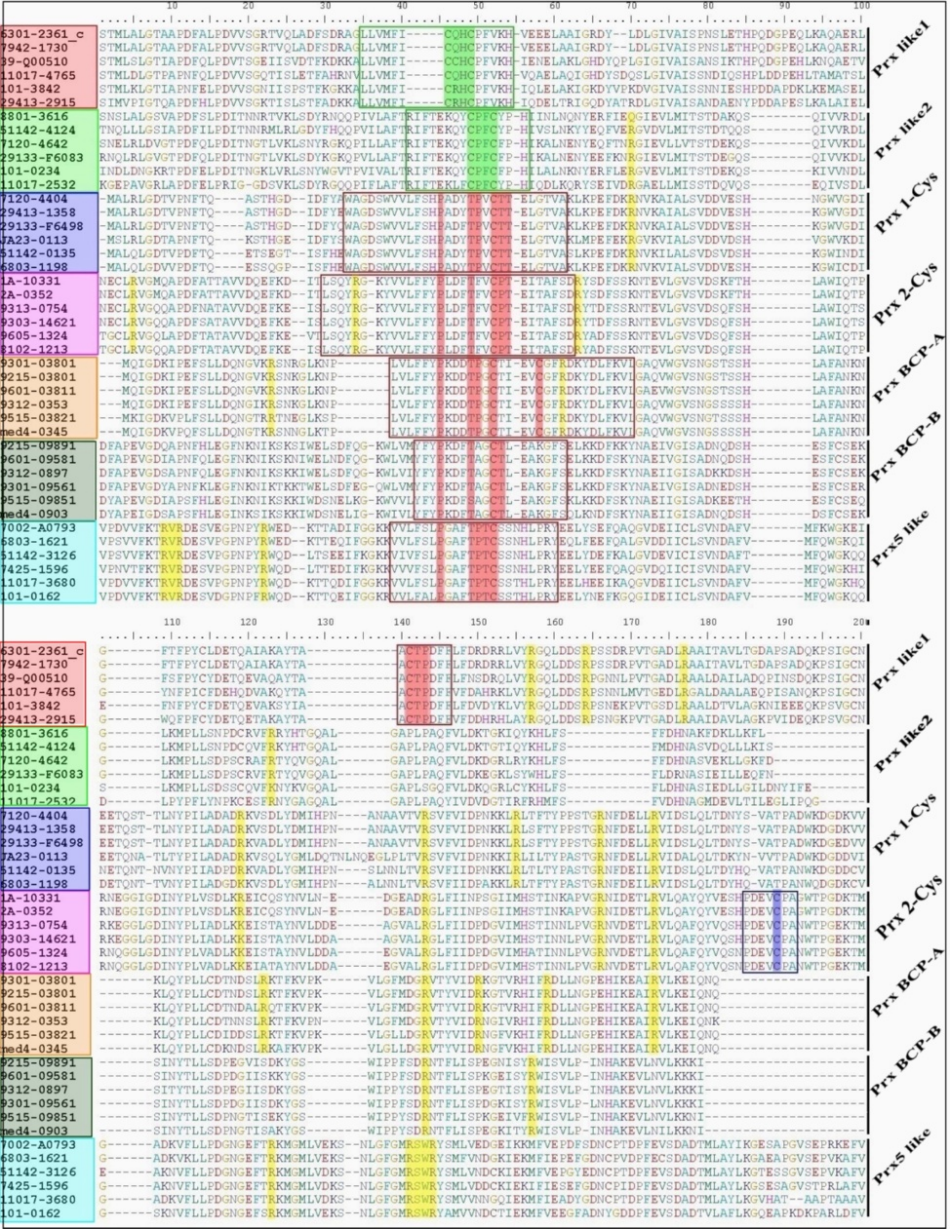
**Conserved cysteine-including domains and arginine in cyanobacterial PRX from different subfamilies.** A total of 42 sequences were used in the alignment of cyanobacterial PRX. Six samples were extracted to represent their own subfamilies. Shades with different colours indicate the distinct PRX subfamilies. Typical catalytic traits of PRX are indicated by the red box. The conserved arginines are indicated by yellow shading.

The redox-active cysteines play a crucial role in the function of all PRXs which were originally divided into two categories, 1-Cys and 2-Cys PRXs, based on the number of cysteine residues directly involved in catalysis. The guanidino group of the conserved arginine is presumed to stabilize the ionized peroxidatic cysteine [41]. We surveyed the cysteine-including motif and the number of conserved arginines from distinct protein sequences to facilitate the classification of different subfamilies (Figure 5 and Table 2). PRXs from 1-Cys and PRX5_like subfamilies contain only one cysteine-including motif (WAGDSWVVLFSHPADYTPVCTTELG) and (VVLXXLPGAFTPTCSS) in the N-terminal, respectively. Two cysteine-including motifs (VVLFFYPLDFTFVCPTEVIAFSD) and (DEVCPA) were found in the N-terminal and C-terminal of the 2-Cys PRXs, respectively. The results from PRX BCP are similar to those from 2-Cys PRXs, whereas the second cysteine-including motif is not conserved among some sequences. Members of PRX-like1 contain a cysteine-including motif in the N-terminal and a cysteine-including motif (AACTPDF) in the C-terminal, whereas PRX_like2 only possess the CXXC motif in the N-terminal. In addition, some arginines are conserved among all PRXs and these are primarily distributed near the C-terminal of the protein sequences.

**Table 2:** Conserved cysteine-including motifs and arginines of PRXs in cyanobacteria

**Subfamily**	**Cys-including**	**Cys-including**	**Position (Arg)**
	**motif 1**	**motif 2**	
Prx 1-Cys	WAGDSWVVLFSHPADYTPVCTTELG		5 (62, 126, 138,
	(26–50)		149 and 156)
Prx 2-Cys	VVLFFYPLDFTFVCPTEVIAFSD	DEVCPA	7 (9, 46, 74, 104,
	(51–73)	(183–188)	140, 163 and 170)
Prx BCP-A	VLYFYPKDDTPGCT	C	2 (64 and 132)
	(44–57)	61	
Prx BCP-B	WVVLYFYPQDFTPGCTLEA	C	1 (160)
	(75–93)	125	
Prx_like1	LLVMFICQHCPFVK	AACTPDF	3 (152, 164
	(52–65)	(134–140)	and 178)
Prx_like2	IFTEXXYCPFCXPH		4 (12, 47, 63
	(64–77)		and 130)
Prx5_like	VVLXXLPGAFTPTCSS		3 (18, 138
	(46–61)		and 141)

The typical catalytic domains and CXXC motif of PRXs was represented by red and blue colour, respectively.

## Discussion

### Peroxiredoxins (PRXs) are an important type of antioxidant protein

Photosynthetic organisms have evolved complicated mechanisms to protect themselves against ROS damage (for a review, see [14,42]). These include enzymatic methods (superoxide dismutases, peroxidases and catalases) that can be used to sequentially detoxify superoxide and hydrogen peroxide [43], and non-enzymatic mechanisms (glutathione, vitamin A, C, E, carotenoids, etc.) [14]. Peroxiredoxins (PRXs) are an important type of antioxidant proteins that are also known as the thioredoxin peroxidases or alkyl-hydroperoxide-reductase-C22 proteins [44,45]. PRXs have been identified from plants [27] and have received considerable attention in recent years. PRXs exert their protective antioxidant role in host cells through their peroxidase activity, which leads to the reduction and detoxification of hydrogen peroxide, peroxynitrite and a wide range of organic hydroperoxides (ROOH) [46-48]. The catalytic efficiency (~ 10^5^ M^-1^ s^-1^) of PRXs is lower than that of better known glutathione peroxidases (~ 10^8^ M^-1^ s^-1^) [46] and catalases (~ 10^6^ M^-1^ s^-1^) [49], which makes their importance as other peroxidases questionable.

What makes PRXs so important and interesting in cyanobacteria? The multi-isoforms and the high abundance of PRXs in a wide range of cells may be the first reason [41,50,51]. Additionally, a recent study revealed that a bacterial PRX (alkyl hydroperoxide reductase C22 (AhpC)), rather than catalase, is responsible for the reduction of endogenously generated hydrogen peroxide [52]. Finally, based on the evaluation of 37 cyanobacterial genomes in this study, it could be found that all *Prochlorococcus marinus* strains and most of the other cyanobacteria do not possess gene(s) with homology to catalase, but possess several genes with homology to PRXs (according to our unpublished results and [53]). Taken together, these characteristics indicate that PRX may actually be important to the detoxification of peroxide in cyanobacterial and other living cells.

### Peroxiredoxins (PRXs): a structural conserved enzyme

Six different sub-classes of PRXs, PRX I–IV (2-Cys PRX), PRX V (Type II PRX) and PRX VI (1-Cys PRX), have been identified from mammalian systems [32]. Among these, only four have been reported in plant systems, namely, 1-Cys PRX, 2-Cys PRX, Type II PRX and PRX Q [29]. According to our results, cyanobacterial PRXs were classified into five major subfamilies (1-Cys, 2-Cys, BCP, PRX5_like, and PRX-like) according to their domain structures. Based on the crystal structures of six PRXs that has been published to date, including four typical 2-Cys PRXs (PRXI, PRXII, TryP and AhpC [54-56], one atypical 2-Cys PRX (PRXV [57]) and one 1-Cys PRX (PRXVI [58]). All PRXs share a similar structure, with each containing a thioredoxin fold and a few additional secondary-structural elements present as insertions. In addition, the structure and sequences of the peroxidatic active site are highly conserved in the protein sequences from all the PRX subfamilies [41]. According to previous study [41], the peroxidatic cysteine in the reduced (SH) form is in a narrow, solvent-accessible pocket formed by a loop-helix structural motif. The cysteine is located in the first turn of the helix and is surrounded by three residues conserved among all classes-Pro44, Thr48 and Arg127 (PRX II numbering) [41]. Our results indicated that the typical catalytic triad of PRXs is found in the N-terminal of those proteins from the 1-Cys, 2-Cys, PRX BCP, and PRX5_like subfamilies (Figure 5 and Table 2). The resolving cysteine near the C-terminal was detected in the proteins from the 2-Cys PRXs subfamily. It is interesting to note that another cysteine was identified in the C-terminal of PRX BCP. This result is not consistent with the results of previous studies, which showed PRX BCP contains the peroxidatic cysteine but without a resolving cysteine [41,59]. However, the role of the second cysteine is still unknown. Members of PRX-like (1 and 2) contain a CXXC motif near the N-terminal that is similar to the classic redox active CXXC motif of Trx [60]. Schultz et al. (1999) claimed that the second cysteine in this motif corresponds to the peroxidatic cysteine of PRXs. However, these proteins do not contain the other two residues of the catalytic triad of PRXs [61]. All PRXs share a highly conserved active-site arginine, which would lower the p*K*a of the peroxidatic cysteine somewhat by stabilizing its thiolate form (see review [41]). As expected, at least one conserved active-site arginine was detected in all cyanobacterial PRXs (Figure 5 and Table 2). Therefore, we speculated that the mechanisms of PRXs of 1-Cys, 2-Cys, PRX BCP, and PRX5_like are similar [41,62], whereas the mechanisms of the PRX_like subfamily are different. According to the definition of the Thioredoxin_like Superfamily [CDD: cl00388], we inferred that PRX_like members do not function as protein disulfide oxidoreductases, even though they containing a Trx-fold domain. However, the catalytic triad of PRXs was discovered in C-terminal sequences from the PRX-like1 subfamily, which exceeded our expectations. Additional experimental results are needed to determine whether this predicted catalytic triad of PRXs is active in PRX-like1. However, such an analysis is beyond the scope of this paper.

### The distribution of PRXs is related to genome sizes and habitat niches

Although the number of *prx* genes and their transcriptional regulation under stress in some cyanobacteria have been reported in previous studies, modification and supplementation is needed with the complete and partial sequencing genomes of several cyanobacterial species. Five genes encoding peroxiredoxin 2-Cys PRX (*sll0755*), 1-Cys PRX (*sll1198*), two PRX Q (*sll0221* and *slr0242*) and one Type II PRX (*sll1621*) were reported in *Synechocystis* sp. PCC 6803 [28,33,34], whereas another gene (ID: *sll1159*, annotation: probable BCP) was detected and classified into the PRX-like2 subfamily. Analysis of the genome of *Synechococcus elongatus* PCC 7942 led to the identification of six putative prx genes [34] with one 1-Cys PRX, one 2-Cys PRX and four PRX Q, while a gene (ID: 7942_1730, annotation: hypothetical protein) was found and classified into the PRX-like1 subfamily. The computational method and the quality of the genome data may be responsible for these different results. Moreover, multi-isoforms (3–12) of genes encoding PRXs were present in all cyanobacteria investigated in the present study. However, the reason for the existence of multiple *prx* genes in these cyanobacteria is still unclear [33,34].

The distribution of putative PRX encoding open reading frames (ORFs) from some sub-families like PRX-like or 1-Cys PRX in different cyanobacteria correlate with the genome size, eco-physiology, and physiological properties of the organisms. Although the number (8–11) of *prx* genes in filamentous cyanobacteria (with large genome size) is higher than those (3–6) from marine unicellular cyanobacteria (with small genome size), the percentage (0.16-0.18%) of PRXs among the total proteins from the former is lower than the latter (0.20-0.30%). Moreover, most of the cyanobacteria possess disproportionate numbers of putative *prx* genes with different genome sizes, indicating that not a basic set is amplified to achieve a larger genome, but that additional functions may be encoded by larger genomes. This result is not consistent with the previous studies who found that not only the number of Serine/threonine kinases and metacaspase genes in filamentous cyanobacteria is higher than those from marine unicellular cyanobacteria, but also the percentage of Serine/threonine kinases and metacaspase genes in the total proteins is higher [7,12]. The reason for this phenomenon may be that PRXs are not the only protein to protect against ROS. For example, other proteins such as catalase, SOD and ferredoxin have been detected in cyanobacteria and the number of genes encoding SODs in filamentous cyanobacteria (with large genome size) is much higher than other cyanobacteria with small genome size [22,26,63]. However, two unicellular cyanobacterial strains inhabiting freshwater (*Synechococcus elongatus* PCC 7942 and *Synechococcus elongatus* PCC 6301) and three unicellular cyanobacterial strains living in hot-springs (*Thermosynechococcus elongatus* BP-1, *Synechococcus* sp. JA-2-3B’a (2–13), and *Synechococcus* sp. JA-3-3Ab) maintain more *prx* genes (7–8) than unicellular cyanobacteria from marine. Considering that unicellular cyanobacterial strains from different habitats share similar genome sizes, various environmental selective pressures may be responsible for the number of *prx* genes in these organisms. The distribution of a small numbers of *prx* genes in cyanobacteria from the ocean is consistent with Serine/threonine kinases and metacaspase genes in cyanobacteria, which are remarkably reduced in marine species [7,12]. Gene loss has been shown to facilitate the acclimatization of these cyanobacteria to the oligotrophic environment of the sea. The major force driving this phenomenon was reportedly a selective process favoring the adaptation of these cyanobacteria, which has been discussed in detail by Alexis Dufresne et al. [64].

### The evolution of PRXs

The protein sequences from the 1-Cys PRX subfamily contains a single conserved catalytic cysteine and is thus denoted 1-Cys PRX [65-67]. Our results revealed that the 1-Cys PRX subfamily was absent from all marine unicellular cyanobacteria except for *Gloeobacter violaceus* PCC 7421 and *Synechococcus* PCC 7002. The phylogenic relationship among 1-Cys PRXs from cyanobacteria, higher plants, and *Metazoa* strongly supports a non-cyanobacterial origin of these proteins in higher plants, indicating that genes encoding 1-Cys PRX are not unique for cyanobacteria and the higher plants do not acquire this gene by endosymbiosis event. Immunochemical study revealed that the 1-Cys PRXs from higher plants are preferentially localized in the nucleus and within the nucleolus [17,65,68]. In addition, the 1-Cys PRXs have been widely recorded in mammalian systems [69]. The 2-Cys PRXs (classical or typical) functioned as a homodimer in a head-to-tail arrangement in which the sulfenic acid derivative of the peroxidatic cysteine of one subunit interacts with the resolving cysteine of the other subunit during the catalytic cycle [70,71]. The 2-Cys PRX subfamily includes chloroplastic 2-Cys PRX, mammalian PRX I-IV and yeast thiol-specific antioxidant (TSA) [17]. Meanwhile, this subfamily is highly conserved among all cyanobacteria. The phylogenetic tree for 2-Cys PRXs revealed that cyanobacteria and higher plants share a common ancestor, which is consistent with the previous studies [70] and the sub-cellular localization (chloroplast) of this protein in *A. thaliana*[17]. PRX BCP subfamily constitutes the largest group of *prx* in cyanobacteria. The *prxq* genes cloned from higher plants are homologous to the bacterioferritin comigratory protein (BCP) from *Escherichia coli*[72] and cluster into the cyanobacterial PRX BCP group. Thus many *prx* genes were originally annotated BCP (PRX Q) in cyanobacteria. PRX Q is the only one that has not been isolated from an animal system [72]. Type II PRXs (A/B/C/D/E/F) from higher plants build a monophyletic group with members from PRX5_like as a sister group, implying that the higher plants acquire this gene via photoautotrophic endosymbiosis. In addition to the above subfamilies, a novel subfamily (PRX-like1 and PRX-like2) was firstly identified from cyanobacteria in this study. Most members of this subfamily are noted as hypothetical proteins that show sequence similarity with PRXs. The structure and mechanism of members of this subfamily are currently unclear.

## Conclusions

Comparative analysis based on the availability of cyanobacterial genome sequences becomes a powerful tool for systematic studies of gene families. Peroxiredoxins comprise one of the most important proteins that play key roles in protecting own cells from the damage of ROS. In this study, 244 putative *prx* genes were identified from 37 species of cyanobacteria using BLASTp, tBLASTn, HMMsearch and SMART domains analysis. Among these putative PRXs, 25 *prx* genes originally annotated as hypothetical proteins were accepted as PRXs firstly in this study. The quantity of *prx* genes in unicellular and filamentous cyanobacteria depends on the genome size, eco-physiology, and ecological habitats. According to the results of CDD domain and phylogenetic analysis, the 244 PRXs were divided into five major groups (1-Cys, 2-Cys, PRX BCP, PRX5_like, and PRX-like). The 2-Cys, PRX BCP, and PRX-like subfamilies are conserved and widely distributed among cyanobacteria. However, PRXs from other subfamilies have only been detected in a few cyanobacterial strains, indicating that they are species or habitat-specific. The typical catalytic trait of PRXs was identified in all PRXs except those from the PRX-like2 subfamily. The proteins from the PRX-like2 subfamily share the classical redox active CXXC motif of thioredoxin. Phylogenetic trees based on the catalytic domains of PRXs from each subfamily coincide well with the phylogenies based on the16s *rRNA*.

## Methods

### Identification of *prx* genes encoding PRX proteins

A total of 37 species of cyanobacteria, including *Prochlorococcus*, *Synechococcus*, *Synechocystis*, *Gloeobacter*, *Cyanothece*, *Microcystis*, *Trichodesmium*, *Acaryochloris*, *Anabaena* and *Nostoc* were used in this analysis. These cyanobacterial genomes were downloaded from the JGI genome portal [39] or Cyanobase [40]. Ten photosynthetic eukaryotic PRX proteins from *Arabidopsis thaliana* and six eukaryotic PRX proteins from *Homo sapiens* were also downloaded from NCBI Genbank [73].

To identify genes encoding peroxiredoxins, eleven previously characterized PRXs from freshwater cyanobacteria *Synechocystis* sp. PCC 6803 and *Synechococcus elongatus* PCC 7942 [34] and ten PRXs from *Arabidopsis thaliana* were used to construct a query protein set. BLASTp [74-76] and tBLASTn [77] programs were conducted locally to search for all *prx* genes from all 37 cyanobacterial genomes using a threshold e-value of 1e-10. Briefly, the *prx* genes encoding PRX proteins used in this study were first identified by local BLASTp and tBLASTn program rather than from the COG database in IMG. Following, we manually checked the extracted proteins by SMART and Pfam analyses to avoid false-positive hits that commonly arise during large-scale automated analyses. PRXs found by this method were added to the query set for another round of BLASTp searches. This procedure was continued until no new proteins were found. Moreover, in order to check for false negatives, two hmm models [Pfam: PF00578] and [Pfam: PF08534] derived from the known PRX proteins were applied to search for genes encoding PRX on all proteins encoded in the 37 cyanobacterial genomes [78,79]. All translated protein sequences of genes encoding PRXs used in this paper were listed in more detail [see Additional file 7.

### Multiple sequence alignment and structure analysis

Proteins identified by the BLAST searches were aligned using ClustalW [80,81] with a gap opening penalty of 10, a gap extension penalty of 0.2, and Gonnet as the weight matrix. The SMART [82] and Pfam 26.0 [78] databases were applied to delete false positives. The alignment was then examined by inspection of the PRX_1cys, PRX_Typ2cys, PRX_BCP, PRX5_like, PRX_like1, and PRX_like2 domains [CDD: cd03016, cd03015, cd03017, cd03013, cd02969, and cd02970] in the NCBI Conserved Domain Database [83]. A protein was accepted as PRX if it was possible to recognize any domain above or known to participate in the function of PRXs. Structural analysis of the obtained PRXs was performed using the SMART (Simple Modular Architecture Research Tool) [82] and the CDD (Conserved Domains Database) [83], methods, relying on hidden Markov models and Reverse Position-Specific BLAST, separately.

### Phylogenetic analysis

Maximum likelihood trees of 16s *rRNA* and PRX proteins were constructed using PhyML [84]. For the 16S *rRNA* tree, the General Time Reversible (GTR) substitution model was selected to assume an estimated proportion of invariant sites and four gamma-distributed rate categories to account for rate heterogeneity across sites [85]. The reliability of internal branches was assessed using the bootstrapping method (400 bootstrap replicates). The Le and Gascuel evolutionary model [86] was selected for analysis of the protein phylogenies assuming an estimated proportion of invariant sites and a gamma correction (four categories). Bootstrap values (BS) were inferred from 400 replicates. Graphical representation and edition of the phylogenetic tree were performed with TreeDyn (v198.3) [87].

### Statistical analyses

Statistical analyses on the relationship between the distribution of genes encoding PRXs and properties of 37 cyanobacterial organisms were performed using the Spearman Rank Correlation test (R). For all of the data analyses, a *p*-value <0.01 was considered statistically significant [88].

## Abbreviations

PRX: Peroxiredoxin; Cys: Cysteine; 1-Cys PRX: 1-cysteine peroxiredoxin; 2-Cys PRX: 2-cysteine peroxiredoxin; PRX Q: Peroxiredoxin Q; PRX II: peroxiredoxin type II; ROS: Reactive oxygen species; Trx: Thioredoxin; AhpC/TSA: Alkyl hydroperoxide reductase/thiol specific antioxid; BCP: Bacterioferritin comigratory protein; RDP: Redoxin domain protein; TSAP: Thiol specific antioxidant protein; HP: Hypothetical protein.

## Competing interests

The authors have declared that no competing interests exist.

## Authors' contributions

H C conceived of the study, participated in the sequence alignment and phylogeny analysis and drafted the manuscript. Y W participated in the domain analysis and statistical analyses. Y W and S Q participated in drafting the manuscript. All authors have read and approved the final manuscript.

## Supplementary Material

Additional file 1 **Table S1.** Cyanobacterial genes encoding peroxiredoxin were predicted by BLAST program (BLASTp and tBLASTn). Note: “-” stands for those do not belong to PRX family. The “complete” and “in complete” present for the complete or partial of the genomes (data collected at 1 Jan, 2012). Table S2 Cyanobacterial genes encoding peroxiredoxin were predicted by hiddenMarkov model (hmmsearch). Note: “*” stands for additional genes encoding PRXs predicted basedon hmmsearch analysis. The “complete” and “in complete” present for the complete or partial of thegenomes (data collected at 1 Jan, 2012). Click here for file

Additional file 2 **Figure S1.** Maximum likelihood tree of PRXs from Prx-BCP subfamily. Click here for file

Additional file 3 **Figure S2.** Maximum likelihood tree of PRXs from 2-Cys subfamily. Click here for file

Additional file 4 **Figure S3.** Maximum likelihood tree of PRXs from Prx-like subfamily. Click here for file

Additional file 5 **Figure S4.** Maximum likelihood tree of PRXs from Prx5_like subfamily. Click here for file

Additional file 6 **Figure S5.** Maximum likelihood tree of PRXs from 1-Cys subfamily. Click here for file

Additional file 7 The translated protein sequences of 244 putative PRXs in 37 cyanobacteria. Click here for file
