# Supplementary material for: Genome-wide analysis of putative peroxiredoxin in unicellular and filamentous cyanobacteria
Source: BMC Evol Biol. 2012 Nov 16;12:220. doi: 10.1186/1471-2148-12-220 (PMC3514251; doi:10.1186/1471-2148-12-220)
Supplement: Additional file 1 — Table S1. Cyanobacterial genes encoding peroxiredoxin were predicted by BLAST program (BLASTp and tBLASTn). Note: “-” stands for those do not belong to PRX family. The “complete” and “in complete” present for the complete or partial of the genomes (data collected at 1 Jan, 2012). Table S2 Cyanobacterial genes encoding peroxiredoxin were predicted by hiddenMarkov model (hmmsearch). Note: “*” stands for additional genes encoding PRXs predicted basedon hmmsearch analysis. The “complete” and “in complete” present for the complete or partial of thegenomes (data collected at 1 Jan, 2012). [file 1471-2148-12-220-S1.pdf]

Additional file 1 Table S1 Cyanobacterial genes encoding peroxiredoxin were predicted by BLAST program (BLASTp and tBLASTn). Note: “-” stands for those do not belong to PRX family. The “complete” and “in complete” present for the complete or partial of the genomes (data collected at 1 Jan, 2012).

**Gene No.**	**Length**	**Annotation**	**Subfamily**
***Acaryochloris marina* MBIC11017 (Complete)**			
AM1_2940	184	AhpC/TSA	Prx BCP-B
AM1_1161	156	BCP	Prx BCP-A
AM1_5436	145	BCP	Prx BCP-A
AM1_6007	184	AhpC/TSA	Prx BCP-A
AM1_2698	202	2-cys Prx, putative	Prx 2-Cys
AM1_A0300	211	Peroxidase/ antioxidant protein	Prx 1-Cys
AM1_4173	215	RDP, putative AhpC/TSA family	Prx_like2-C
AM1_5494	211	AhpC/TSA	Prx 1-Cys
AM1_2532	180	AhpC/TSA family protein	Prx_like2-B
AM1_5336	179	RDP, putative AhpC/TSA family	Prx_like2-A
AM1_4765	193	RDP, putative AhpC/TSA family	Prx_like1
AM1_3680	190	Prx, putative	Prx5_like
***Anabaena* sp. PCC 7120 (Complete)**			
all2556	185	BCP	Prx BCP-B
alr2503	159	BCP	Prx BCP-A
all2375	145	Similar to BCP	Prx BCP-A
alr3183	153	BCP	Prx BCP-A
alr4641	203	Prx	Prx 2-Cys
alr4404	212	AhpC/TSA family protein	Prx 1-Cys
alr4642	213	Putative TSAP	Prx_like2-D
all0513	187	HP	Prx_like1
alr1206	184	HP	Prx5_like
***Anabaena variabilis* ATCC 29413 (Complete)**			
Ava_0485	185	AhpC/TSA	Prx BCP-B
Ava_0435	159	AhpC/TSA	Prx BCP-A
Ava_0194	145	AhpC/TSA	Prx BCP-A
Ava_3881	153	AhpC/TSA	Prx BCP-A
Ava_2024	203	AhpC/TSA	Prx 2-Cys
Ava_1358	212	1-Cys Prx	Prx 1-Cys
Ava_2023	178	Putative TSAP	Prx_like2-C
Ava_2915	187	HP	Prx_like1
Ava_0627	184	HP	Prx_like1
***Arthrospira platensis* NIES-39 (In complete)**			
NIES39_L02380	181	Putative BCP	Prx BCP-B
NIES39_E01510	144	Putative BCP	Prx BCP-A
NIES39_O06760	155	Putative BCP	Prx BCP-A
NIES39_A03490	153	BCP	Prx BCP-A
NIES39_M00250	198	Prx	Prx 2-Cys

NIES39_E02230	377	Probable BCP	Prx BCP-A
NIES39_F00560	212	Putative Prx	Prx 1-Cys
NIES39_J00990	183	Putative TSAP	Prx_like2-C
NIES39_Q00510	186	HP	Prx_like1
NIES39_C03410	179	HP	Prx_like1
NIES39_D06120	127	Trx M	-
***Cyanothece* sp. ATCC 51142 (Complete)**			
cce_1296	180	BCP	Prx BCP-B
cce_3358	144	Putative BCP	Prx BCP-A
cce_2409	199	Trx peroxidase	Prx 2-Cys
cce_0135	211	Putative rehydrin	Prx 1-Cys
cce_4124	179	Putative TSAP	Prx_like2-C
cce_3126	190	Prx	Prx5_like
cce_3631	196	HP	Prx_like1
***Cyanothece* sp. PCC 7424 (Complete)**			
PCC7424_2927	183	AhpC/TSA	Prx BCP-B
PCC7424_4280	157	AhpC/TSA	Prx BCP-A
PCC7424_3638	144	AhpC/TSA	Prx BCP-A
PCC7424_5046	151	AhpC/TSA	Prx BCP-A
PCC7424_0090	197	AhpC/TSA	Prx 2-Cys
PCC7424_0397	211	Peroxidase	Prx 1-Cys
PCC7424_1402	176	AhpC/TSA	Prx_like2-B
PCC7424_4031	191	AhpC/TSA	Prx_like1
PCC7424_2508	245	Glutaredoxin-family domain protein	Prx5_like
***Cyanothece* sp. PCC 7425 (Complete)**			
Cyan7425_2289	183	AhpC/TSA	Prx BCP-B
Cyan7425_1511	161	AhpC/TSA	Prx BCP-A
Cyan7425_3716	153	AhpC/TSA	Prx BCP-A
Cyan7425_5136	149	AhpC/TSA	Prx BCP-A
Cyan7425_0928	198	AhpC/TSA	Prx 2-Cys
Cyan7425_0215	213	Peroxidase	Prx 1-Cys
Cyan7425_4300	185	AhpC/TSA	Prx_like2-C
Cyan7425_0955	193	AhpC/TSA	Prx_like1
Cyan7425_1596	190	Prx	Prx5_like
***Cyanothece* sp. PCC 8801 (Complete)**			
PCC8801_1115	195	AhpC/TSA	Prx BCP-B
PCC8801_3492	167	AhpC/TSA	Prx BCP-A
PCC8801_1631	144	AhpC/TSA	Prx BCP-A
PCC8801_1491	199	AhpC/TSA	Prx 2-Cys
PCC8801_4218	211	Peroxidase	Prx 1-Cys
PCC8801_3616	178	AhpC/TSA	Prx_like2-C
PCC8801_4240	193	AhpC/TSA	Prx_like1
***Gloeobacter violaceus* PCC 7421 (Complete)**			
glr3389	180	BCP	Prx BCP-B

glr2376	159	probable BCP	Prx BCP-A
glr2375	145	probable BCP	Prx BCP-A
gll0506	148	BCP	Prx BCP-A
gll3158	184	Probable Prx	Prx 2-Cys
glr2155	212	AhpC/TSA family protein	Prx 1-Cys
glr0029	179	HP	Prx_like2-B
glr3964	203	HP	Prx_like2-C
glr2108	193	HP	Prx_like1
gll3157	151	HP	-
***Microcystis aeruginosa* NIES-843 (Complete)**			
MAE15330	180	BCP	Prx BCP-B
MAE44920	155	BCP homolog	Prx BCP-A
MAE60930	144	BCP	Prx BCP-A
MAE35830	199	Trx peroxidase	Prx 2-Cys
MAE36510	211	Prx	Prx 1-Cys
MAE62780	243	Putative Prx	Prx5_like
MAE59730	193	HP	Prx_like1
***Nostoc punctiforme* ATCC 29133 (Complete)**			
Npun_F2425	185	AhpC/TSA	Prx BCP-B
Npun_R6477	159	AhpC/TSA	Prx BCP-A
Npun_F2872	152	AhpC/TSA	Prx BCP-A
Npun_R0493	145	AhpC/TSA	Prx BCP-A
Npun_F6082	203	AhpC/TSA	Prx 2-Cys
Npun_F6498	212	AhpC/TSA	Prx 1-Cys
Npun_F6083	178	AhpC/TSA	Prx_like2-C
Npun_F6135	184	AhpC/TSA	Prx_like1
Npun_F2528	193	AhpC/TSA	Prx_like1
Npun_R4657	244	Glutaredoxin family protein	Prx5_like
***Prochlorococcus marinus* MED4 (In complete)**			
PMM0903	177	AhpC/TSA	Prx BCP-B
PMM0079	155	Putative BCP	Prx BCP-A
PMM0345	149	Putative BCP	Prx BCP-A
PMM0856	194	Trx peroxidase	Prx 2-Cys
PMM0283	197	Conserved HP	Prx_like1
***Prochlorococcus marinus* MIT9312 (Complete)**			
PMT9312_0897	177	AhpC/TSA	Prx BCP-B
PMT9312_0082	155	Putative BCP	Prx BCP-A
PMT9312_0353	149	Putative BCP	Prx BCP-A
PMT9312_0944	194	Trx peroxidase	Prx 2-Cys
***Prochlorococcus marinus* MIT9313 (Complete)**			
PMT1012	187	AhpC/TSA	Prx BCP-B
PMT1622	155	Putative BCP	Prx BCP-A
PMT0387	153	Putative BCP	Prx BCP-A
PMT0754	200	Trx peroxidase	Prx 2-Cys

PMT1186	190	AhpC/TSA	Prx5_like
***Prochlorococcus marinus* SS120 (Complete)**			
Pro0932	183	Prx	Prx BCP-B
Pro0093	155	Prx	Prx BCP-A
Pro0978	197	Prx, AhpC/TSA family	Prx 2-Cys
***Prochlorococcus marinus* str. AS9601 (Complete)**			
A9601_09581	177	AhpC/TSA	Prx BCP-B
A9601_00921	155	Putative BCP	Prx BCP-A
A9601_03811	149	Putative BCP	Prx BCP-A
A9601_10131	194	Trx peroxidase	Prx 2-Cys
***Prochlorococcus marinus* str. MIT 9211 (In complete)**			
P9211_08571	183	AhpC/TSA	Prx BCP-B
P9211_15101	150	Prx	Prx BCP-A
P9211_00881	158	Putative BCP	Prx BCP-A
P9211_09041	199	Trx peroxidase	Prx 2-Cys
***Prochlorococcus marinus* str. MIT 9215 (In complete)**			
P9215_09891	177	AhpC/TSA	Prx BCP-B
P9215_00921	155	Putative BCP	Prx BCP-A
P9215_03801	149	Putative BCP	Prx BCP-A
P9215_10441	194	Trx peroxidase	Prx 2-Cys
P9215_03081	197	HP	Prx_like1
P9215_11961	107	Trx	-
***Prochlorococcus marinus* str. MIT 9301 (In complete)**			
P9301_09561	177	AhpC/TSA	Prx BCP-B
P9301_00911	155	Putative BCP	Prx BCP-A
P9301_03801	149	Putative BCP	Prx BCP-A
P9301_10121	194	Trx peroxidase	Prx 2-Cys
P9301_02651	153	Trx-like protein TxlA	-
***Prochlorococcus marinus* str. MIT 9303 (Complete)**			
P9303_10801	173	AhpC/TSA	Prx BCP-B
P9303_02611	155	Putative BCP	Prx BCP-A
P9303_19011	153	Putative BCP	Prx BCP-A
P9303_14621	200	Trx peroxidase	Prx 2-Cys
P9303_08311	190	Prx	Prx5_like
P9303_25151	221	HP	Prx_like1
***Prochlorococcus marinus* str. MIT 9515 (In complete)**			
P9515_09851	177	AhpC/TSA	Prx BCP-B
P9515_00891	155	Putative BCP	Prx BCP-A
P9515_03821	149	Putative BCP	Prx BCP-A
P9515_09331	194	Trx peroxidase	Prx 2-Cys
***Prochlorococcus marinus* str. NATL1A (Complete)**			
NATL1_09761	184	AhpC/TSA	Prx BCP-B
NATL1_15651	149	Prx	Prx BCP-A

NATL1_16391	151	Prx	Prx BCP-A
NATL1_01441	155	Putative BCP	Prx BCP-A
NATL1_10331	198	Trx peroxidase	Prx 2-Cys
NATL1_03631	206	HP	Prx_like1
***Prochlorococcus marinus* str. NATL2A (Complete)**			
PMN2A_0304	184	AhpC/TSA	Prx BCP-B
PMN2A_0727	149	Putative BCP	Prx BCP-A
PMN2A_0787	151	Putative BCP	Prx BCP-A
PMN2A_1443	155	Putative BCP	Prx BCP-A
PMN2A_0352	198	Trx peroxidase	Prx 2-Cys
PMN2A_1649	206	HP	Prx_like1
***Synechococcus elongatus* PCC 6301 (Complete)**			
syc0883_d	181	BCP	Prx BCP-B
syc2152_c	150	BCP	Prx BCP-A
syc2287_c	157	BCP	Prx BCP-A
syc1915_d	145	BCP	Prx BCP-A
syc1793_d	201	Trx peroxidase	Prx 2-Cys
syc1657_c	211	Antioxidant protein] rehydrin	Prx 1-Cys
syc2361_c	195	HP	Prx_like1
***Synechococcus elongatus* PCC 7942 (Complete)**			
Synpcc7942_0642	181	BCP	Prx BCP-B
Synpcc7942_1942	150	BCP	Prx BCP-A
Synpcc7942_1806	157	BCP	Prx BCP-A
Synpcc7942_2180	145	BCP	Prx BCP-A
Synpcc7942_2309	198	Trx peroxidase	Prx 2-Cys
Synpcc7942_2449	211	1-Cys Prx	Prx 1-Cys
Synpcc7942_1730	196	HP	Prx_like1
***Synechococcus* sp. CC9311 (Complete)**			
sync_1400	183	AhpC/TSA	Prx BCP-B
sync_2508	155	BCP	Prx BCP-A
sync_1556	152	BCP	Prx BCP-A
sync_1322	192	Trx peroxidase	Prx 2-Cys
sync_0254	209	Trx family protein	Prx_like1
sync_0689	192	Prx 2 family protein	Prx5_like
***Synechococcus* sp. CC9605 (Complete)**			
Syncc9605_1406	186	Twin-arginine translocation pathway	Prx BCP-B
Syncc9605_1141	151	Putative BCP	Prx BCP-A
Syncc9605_2307	155	Putative BCP	Prx BCP-A
Syncc9605_1324	200	Trx peroxidase	Prx 2-Cys
Syncc9605_0215	194	Conserved HP	Prx_like1
Syncc9605_1945	124	Trx	-
***Synechococcus* sp. CC9902 (Complete)**			
Syncc9902_1080	186	AhpC/TSA	Prx BCP-B

Syncc9902_1316	151	Putative BCP	Prx BCP-A
Syncc9902_0386	155	Putative BCP	Prx BCP-A
Syncc9902_1150	203	Trx peroxidase	Prx 2-Cys
Syncc9902_0982	175	Putative superoxide dismutase	-
Syncc9902_0354	309	Trx domain 2	-
Syncc9902_0242	222	Conesrved HP	Prx_like1
Syncc9902_0720	107	Trx	-
***Synechococcus* sp. JA-2-3B'a(2-13) (Complete)**			
CYB_2186	173	Putative Prx	Prx BCP-B
CYB_0523	154	BCP	Prx BCP-A
CYB_1057	145	Antioxidant, AhpC/Tsa family	Prx BCP-A
CYB_1376	155	Antioxidant, AhpC/Tsa family	Prx BCP-A
CYB_2254	202	Antioxidant, AhpC/Tsa family	Prx 2-Cys
CYB_0113	216	Antioxidant, AhpC/Tsa family	Prx 1-Cys
CYB_0140	193	AhpC/TSA family protein	Prx_like2-B
CYB_2461	206	HP	Prx_like1
***Synechococcus* sp. JA-3-3Ab (Complete)**			
CYA_0907	177	Antioxidant, AhpC/Tsa family	Prx BCP-B
CYA_2145	154	BCP	Prx BCP-A
CYA_0672	145	Antioxidant, AhpC/Tsa family	Prx BCP-A
CYA_2305	155	Antioxidant, AhpC/TSA family	Prx BCP-A
CYA_0537	202	Antioxidant, AhpC/Tsa family	Prx 2-Cys
CYA_2849	216	Antioxidant, AhpC/Tsa family	Prx 1-Cys
CYA_1949	193	AhpC/TSA family protein	Prx_like2-B
CYA_2781	196	HP	Prx_like1
***Synechococcus* sp. PCC 7002 (Complete)**			
SYNPCC7002_A0109	192	AhpC/TSA	Prx BCP-B
SYNPCC7002_A1108	154	BCP	Prx BCP-A
SYNPCC7002_A0558	195	AhpC/TSA	Prx 2-Cys
SYNPCC7002_A0320	211	AhpC/TSA family	Prx 1-Cys
SYNPCC7002_A1383	193	Prx	Prx_like1
SYNPCC7002_A0793	187	AhpC/TSA family protein	Prx5_like
***Synechococcus* sp. RCC307 (Complete)**			
SynRCC307_1384	181	Prx	Prx BCP-B
SynRCC307_0392	156	Prx	Prx BCP-A
SynRCC307_1342	151	Prx	Prx BCP-A
SynRCC307_1238	199	Prx, AhpC/TSA family	Prx 2-Cys
SynRCC307_0386	223	Prx	Prx 1-Cys
SynRCC307_2285	182	Possible Trx	Prx_like1
***Synechococcus* sp. WH 7803 (Complete)**			
SynWH7803_1236	183	Prx	Prx BCP-B
SynWH7803_1042	152	Prx	Prx BCP-A
SynWH7803_2172	157	Prx	Prx BCP-A
SynWH7803_1118	200	Prx, AhpC/TSA family	Prx 2-Cys

***Synechococcus* sp. WH8102 (Complete)**			
SYNW1280	186	AhpC/TSA	Prx BCP-B
SYNW1015	151	Putative BCP	Prx BCP-A
SYNW2162	155	Putative BCP	Prx BCP-A
SYNW1213	200	Trx peroxidase	Prx 2-Cys
SYNW0221	194	Conserved HP	Prx_like1
SYNW0724	107	Trx	-
***Synechocystis* sp. PCC 6803 (Complete)**			
sll0221	184	BCP	Prx BCP-B
slr0242	160	BCP homolog	Prx BCP-A
sll0755	200	Trx peroxidase	Prx 2-Cys
slr1198	211	Antioxidant protein	Prx 1-Cys
sll1159	218	probable BCP	Prx_like2-C
sll1621	189	AhpC/TSA family protein	Prx5_like
***Trichodesmium erythraeum* IMS101 (Complete)**			
Tery_2703	183	Redoxin	Prx BCP-B
Tery_3959	146	Redoxin	Prx BCP-A
Tery_3182	154	Redoxin	Prx BCP-A
Tery_0235	199	AhpC/TSA	Prx 2-Cys
Tery_5038	212	1-Cys Prx	Prx 1-Cys
Tery_0234	183	Redoxin	Prx_like2-C
Tery_3842	193	Redoxin	Prx_like1
Tery_0162	189	Redoxin	Prx5_like
***Thermosynechococcus elongatus* BP-1 (Complete)**			
tlr1194	179	BCP	Prx BCP-B
tll1451	156	BCP homolog	Prx BCP-A
tlr1198	145	BCP	Prx BCP-A
tll1454	197	Trx peroxidase	Prx 2-Cys
tlr2261	211	AhpC/TSA family protein	Prx 1-Cys
tsr0473	76	Probable BCP	-
tlr1289	196	HP	Prx_like2-C
tlr1788	193	HP	Prx_like1

Additional file 1 Table S2 Cyanobacterial genes encoding peroxiredoxin were predicted by hidden Markov model (hmmsearch). Note: "*" stands for additional genes encoding PRXs predicted based on hmmsearch analysis. The "complete" and "in complete" present for the complete or partial of the genomes (data collected at 1 Jan, 2012).

Full sequence			Best 1 domain			Dom			
**E-value**	**score**	**bias**	**E-value**	**score**	**bias**	**exp**	**N**	**Sequence**	**Description**
***Prochlorococcus marinus* str. NATL1A (Complete)**									
2.3e-40	134.4	0.0	3.3e-40	133.9	0.0	1.2	1	NATL1_10331	thioredoxin peroxidase
5.2e-37	123.6	0.0	5.9e-37	123.4	0.0	1.0	1	NATL1_16391	Peroxiredoxin
6.5e-36	120.0	0.0	7.7e-36	119.8	0.0	1.1	1	NATL1_01441	putative bacterioferritin comigratory (BCP) protein
1.5e-35	118.8	0.0	1.9e-35	118.5	0.0	1.1	1	NATL1_09761	Alkyl hydroperoxide reductase/ Thiol specific antioxidant protein
7.5e-33	110.1	0.0	8.8e-33	109.9	0.0	1.1	1	NATL1_15651	Peroxiredoxin
***Prochlorococcus marinus* str. NATL2A (Complete)**									
2.3e-40	134.4	0.0	3.3e-40	133.9	0.0	1.2	1	PMN2A_0352	thioredoxin peroxidase
5.1e-37	123.6	0.0	5.9e-37	123.4	0.0	1.0	1	PMN2A_0787	putative bacterioferritin comigratory protein
8.7e-37	122.8	0.0	1e-36	122.6	0.0	1.1	1	PMN2A_1443	putative bacterioferritin comigratory (BCP) protein
6e-35	116.9	0.0	7.5e-35	116.6	0.0	1.1	1	PMN2A_0304	alkyl hydroperoxide reductase/thiol specific antioxidant protein
7e-33	110.2	0.0	8.1e-33	110.0	0.0	1.1	1	PMN2A_0727	putative bacterioferritin comigratory protein
***Synechococcus* sp. JA-2-3B'a(2-13) (Complete)**									
4e-46	153.4	0.0	4.7e-46	153.2	0.0	1.0	1	CYB_0523	bacterioferritin comigratory protein
3.7e-44	147.0	0.0	5.3e-44	146.5	0.0	1.2	1	CYB_2254	antioxidant, AhpC/Tsa family
1.6e-42	141.7	0.0	1.9e-42	141.5	0.0	1.1	1	CYB_2186	putative peroxiredoxin
1.5e-41	138.6	0.0	1.8e-41	138.4	0.0	1.0	1	CYB_1057	antioxidant, AhpC/Tsa family
2.8e-37	124.8	0.0	3.3e-37	124.6	0.0	1.0	1	CYB_1376	antioxidant, AhpC/TSA family
4e-36	121.1	0.0	5.5e-36	120.6	0.0	1.2	1	CYB_0113	antioxidant, AhpC/Tsa family
7.3e-34	113.8	0.0	9.1e-34	113.5	0.0	1.1	1	CYB_0140	AhpC/TSA family protein
6.9e-27	91.2	0.0	9.1e-27	90.8	0.0	1.2	1	CYB_2461	hypothetical protein
***Synechococcus* sp. JA-3-3Ab (Complete)**									
1.6e-45	151.4	0.0	1.9e-45	151.2	0.0	1.0	1	CYA_2145	bacterioferritin comigratory protein
2.8e-44	147.4	0.0	3.9e-44	146.9	0.0	1.2	1	CYA_0537	antioxidant, AhpC/Tsa family
2.6e-43	144.2	0.0	3.2e-43	143.9	0.0	1.1	1	CYA_0907	antioxidant, AhpC/Tsa family
1.1e-42	142.2	0.0	1.3e-42	142.0	0.0	1.0	1	CYA_0672	antioxidant, AhpC/Tsa family
6.8e-36	120.3	0.0	9e-36	119.9	0.0	1.2	1	CYA_2849	antioxidant, AhpC/Tsa family
8.8e-36	119.9	0.0	1e-35	119.7	0.0	1.0	1	CYA_2305	antioxidant, AhpC/TSA family
9.8e-35	116.5	0.0	1.2e-34	116.2	0.0	1.1	1	CYA_1949	AhpC/TSA family protein
1.5e-26	90.1	0.0	2e-26	89.7	0.0	1.2	1	CYA_2781	hypothetical protein
***Arthrospira platensis* NIES-39 (In complete)**									
2.6e-43	145.5	0.0	3.2e-43	145.2	0.0	1.1	1	NIES39_A03490	bacterioferritin comigratory protein
3.5e-41	138.6	0.0	4.2e-41	138.3	0.0	1.1	1	NIES39_L02380	putative bacterioferritin comigratory protein
3.9e-41	138.4	0.0	4.9e-41	138.1	0.0	1.1	1	NIES39_M00250	peroxiredoxin
1.4e-40	136.7	0.0	1.6e-40	136.5	0.0	1.0	1	NIES39_E01510	putative bacterioferritin comigratory protein
1.1e-39	133.7	0.0	1.4e-39	133.5	0.0	1.1	1	NIES39_O06760	putative bacterioferritin comigratory protein
8.7e-34	114.7	0.0	1.2e-33	114.3	0.0	1.2	1	NIES39_F00560	putative peroxiredoxin
1.7e-28	97.6	0.0	2.1e-28	97.3	0.0	1.1	1	NIES39_J00990	putative thiol-specific antioxidant protein
8.3e-22	76.0	0.0	1.2e-21	75.5	0.0	1.2	1	NIES39_E02230	probable bacterioferritin comigratory protein
7.6e-21	72.9	0.0	9.9e-21	72.5	0.0	1.2	1	NIES39_Q00510	hypothetical protein
1.7e-19	68.5	0.0	2.1e-19	68.2	0.0	1.1	1	NIES39_C03410	hypothetical protein
7.8e-15	53.5	0.0	9.5e-15	53.2	0.0	1.1	1	NIES39_M02570	putative peroxiredoxin*
***Trichodesmium erythraeum* IMS101 (Complete)**									
4.9e-45	150.5	0.0	6.2e-45	150.2	0.0	1.1	1	Tery_0235	alkyl hydroperoxide reductase/ Thiol specific antioxidant protein

9.6e-43	143.1	0.0	1.1e-42	142.9	0.0	1.0	1	Tery_3959	redoxin
1.3e-42	142.7	0.0	1.6e-42	142.4	0.0	1.1	1	Tery_3182	redoxin
2.6e-40	135.2	0.0	3.6e-40	134.8	0.0	1.2	1	Tery_2703	redoxin
4.8e-36	121.4	0.0	6.2e-36	121.1	0.0	1.1	1	Tery_5038	1-Cys peroxiredoxin
3.3e-24	83.2	0.0	4.2e-24	82.8	0.0	1.2	1	Tery_0234	redoxin
3.7e-21	73.4	0.1	5e-21	72.9	0.0	1.2	1	Tery_3842	redoxin
3.3e-15	54.1	0.0	4.2e-15	53.8	0.0	1.2	1	Tery_0162	redoxin
4.1e-06	24.8	0.1	5e-06	24.5	0.0	1.1	1	Tery_2011	thioredoxin domain-containing protein*
***Prochlorococcus marinus* SS120 (Complete)**									
1.4e-39	131.7	0.0	1.8e-39	131.3	0.0	1.2	1	Pro0978	Peroxiredoxin, AhpC/TSA family
1.1e-35	119.0	0.0	1.3e-35	118.8	0.0	1.1	1	Pro0093	Peroxiredoxin
3e-35	117.7	0.0	3.6e-35	117.4	0.0	1.1	1	Pro0932	Peroxiredoxin
***Synechococcus* sp. RCC307 (Complete)**									
4.8e-44	146.5	0.0	7.2e-44	145.9	0.0	1.3	1	SynRCC307_1238	Peroxiredoxin, AhpC/TSA family
3.8e-40	133.9	0.0	4.5e-40	133.6	0.0	1.1	1	SynRCC307_0392	Peroxiredoxin
4.3e-35	117.6	0.0	5.1e-35	117.3	0.0	1.1	1	SynRCC307_1342	Peroxiredoxin
7.9e-34	113.5	0.0	9.6e-34	113.2	0.0	1.1	1	SynRCC307_1384	Peroxiredoxin
2.2e-33	112.1	0.0	2.9e-33	111.7	0.0	1.2	1	SynRCC307_0386	Peroxiredoxin
***Microcystis aeruginosa* NIES-843 (Complete)**									
5.5e-43	144.4	0.0	6.8e-43	144.0	0.0	1.1	1	MAE35830	thioredoxin peroxidase
5.1e-42	141.2	0.0	6.3e-42	140.9	0.0	1.1	1	MAE15330	bacterioferritin comigratory protein
3.8e-40	135.2	0.1	4.4e-40	135.0	0.0	1.0	1	MAE60930	bacterioferritin comigratory protein
2.2e-39	132.7	0.0	2.6e-39	132.5	0.0	1.1	1	MAE44920	bacterioferritin comigratory protein homolog
5.3e-33	112.1	0.0	7.2e-33	111.7	0.0	1.2	1	MAE36510	peroxiredoxin
1.4e-21	75.2	0.0	1.8e-21	74.8	0.0	1.2	1	MAE59730	hypothetical protein
3e-16	58.0	0.0	4.1e-15	54.3	0.0	2.0	2	MAE62780	putative peroxiredoxin
***Synechococcus elongatus* PCC 6301 (Complete)**									
7.7e-44	145.8	0.0	8.9e-44	145.6	0.0	1.0	1	syc2152_c	bacterioferritin comigratory protein homolog
5e-43	143.2	0.0	6.9e-43	142.7	0.0	1.2	1	syc1793_d	thioredoxin peroxidase
9.1e-40	132.7	0.0	1.1e-39	132.4	0.0	1.1	1	syc2287_c	bacterioferritin comigratory protein
2.6e-39	131.2	0.0	3e-39	131.0	0.0	1.1	1	syc0883_d	bacterioferritin comigratory protein
4.2e-38	127.3	0.0	4.9e-38	127.1	0.0	1.0	1	syc1915_d	bacterioferritin comigratory protein
1.2e-33	112.9	0.0	1.5e-33	112.6	0.0	1.1	1	syc1657_c	antioxidant protein] rehydrin
8.9e-20	68.1	0.0	1.2e-19	67.7	0.0	1.2	1	syc2361_c	hypothetical protein
***Synechocystis* sp. PCC 6803 (Complete)**									
1.6e-43	145.4	0.0	2e-43	145.0	0.0	1.2	1	sll0755	thioredoxin peroxidase
5.2e-42	140.4	0.0	6.2e-42	140.2	0.0	1.1	1	sll0221	bacterioferritin comigratory protein
5.4e-42	140.4	0.0	6.4e-42	140.1	0.0	1.1	1	slr0242	bacterioferritin comigratory protein homolog
1.3e-33	113.3	0.0	1.8e-33	112.9	0.0	1.2	1	slr1198	antioxidant protein
3.9e-33	111.8	0.0	5.3e-33	111.3	0.0	1.2	1	sll1159	probable bacterioferritin comigratory protein
2.5e-21	73.6	0.0	3e-21	73.4	0.0	1.1	1	sll1289	hypothetical protein
***Synechococcus* sp. PCC 7002 (Complete)**									
1e-43	145.7	0.0	1.4e-43	145.3	0.0	1.1	1	SYNPCC7002_A0558	Alkyl hydroperoxide reductase; peroxiredoxi
2.5e-41	138.0	0.0	2.9e-41	137.8	0.0	1.0	1	SYNPCC7002_A1108	bacterioferritin comigratory protein
1e-40	136.1	0.0	1.2e-40	135.8	0.0	1.1	1	SYNPCC7002_A0109	AhpC/TSA family (bacterioferritin comigratory protein)
3e-34	115.2	0.0	3.9e-34	114.8	0.0	1.1	1	SYNPCC7002_A0320	AhpC/TSA family
5.4e-24	82.0	0.0	6.8e-24	81.7	0.0	1.1	1	SYNPCC7002_A1383	Peroxiredoxin
2.4e-16	57.4	0.0	3.2e-16	56.9	0.0	1.2	1	SYNPCC7002_A0793	AhpC/TSA family protein
***Anabaena* sp. PCC 7120 (Complete)**									
5e-47	157.4	0.0	6.3e-47	157.0	0.0	1.1	1	alr4641	peroxiredoxin
7.2e-43	143.9	0.0	8.6e-43	143.7	0.0	1.1	1	alr3183	bacterioferritin comigratory protein
6.7e-42	140.8	0.0	7.9e-42	140.6	0.0	1.1	1	alr2503	bacterioferritin comigratory protein
9.3e-42	140.3	0.1	1.1e-41	140.2	0.0	1.0	1	all2375	similar to bacterioferritin comigratory protein

1.2e-39	133.5	0.0	1.5e-39	133.2	0.0	1.1	1	all2556	bacterioferritin comigratory protein
2.7e-32	109.8	0.0	3.8e-32	109.3	0.0	1.2	1	alr4404	AhpC/TSA family protein
2.6e-26	90.4	0.0	3.6e-26	90.0	0.0	1.2	1	alr4642	putative thiol-specific antioxidant protein
4.7e-22	76.7	0.0	5.9e-22	76.4	0.0	1.1	1	all0513	hypothetical protein
5.1e-18	63.7	0.0	6.1e-18	63.4	0.0	1.1	1	alr1206	hypothetical protein
***Gloeobacter violaceus* PCC 7421 (Complete)**									
1.6e-41	139.1	0.0	1.8e-41	138.9	0.0	1.0	1	glr2375	probable bacterioferritin comigratory protein
2.2e-40	135.4	0.0	2.6e-40	135.2	0.0	1.1	1	glr2376	probable bacterioferritin comigratory protein
3.2e-40	134.9	0.0	4e-40	134.6	0.0	1.1	1	gll3158	probable peroxiredoxin
2.1e-38	129.1	0.0	2.5e-38	128.8	0.0	1.1	1	glr3389	bacterioferritin comigratory protein
8.1e-38	127.2	0.0	1.1e-37	126.7	0.0	1.2	1	glr2155	AhpC/TSA family protein
2e-32	109.7	0.0	2.4e-32	109.5	0.0	1.1	1	gll0506	bacterioferritin comigratory protein
3.8e-28	95.9	0.0	4.8e-28	95.6	0.0	1.1	1	glr0029	hypothetical protein
1.2e-19	68.5	0.0	1.8e-19	67.9	0.0	1.3	1	glr2108	hypothetical protein
7.5e-16	56.2	0.0	1.1e-15	55.7	0.0	1.2	1	glr3964	hypothetical protein
***Cyanothece* sp. PCC 7424 (Complete)**									
2.1e-45	152.0	0.0	2.7e-45	151.7	0.0	1.1	1	PCC7424_0090	alkyl hydroperoxide reductase/ Thiol specific antioxidant protein
8.6e-43	143.6	0.0	1.1e-42	143.3	0.0	1.1	1	PCC7424_5046	alkyl hydroperoxide reductase/ Thiol specific antioxidant protein
1.3e-42	143.0	0.0	1.6e-42	142.7	0.0	1.1	1	PCC7424_2927	alkyl hydroperoxide reductase/ Thiol specific antioxidant protein
1.9e-42	142.4	0.1	2.2e-42	142.2	0.1	1.0	1	PCC7424_3638	alkyl hydroperoxide reductase/ Thiol specific antioxidant protein
5.3e-42	141.0	0.0	6.1e-42	140.8	0.0	1.0	1	PCC7424_4280	alkyl hydroperoxide reductase/ Thiol specific antioxidant protein
2.7e-36	122.6	0.0	3.7e-36	122.1	0.0	1.2	1	PCC7424_0397	Peroxidase
1.3e-28	97.7	0.0	1.6e-28	97.5	0.0	1.1	1	PCC7424_1402	alkyl hydroperoxide reductase/ Thiol specific antioxidant protein
2.2e-23	80.9	0.1	3.1e-23	80.4	0.0	1.2	1	PCC7424_4031	alkyl hydroperoxide reductase/ Thiol specific antioxidant protein
1.8e-17	61.8	0.0	1.1e-15	56.1	0.0	2.1	2	PCC7424_2508	glutaredoxin-family domain protein
***Cyanothece* sp. PCC 7425 (Complete)**									
2.3e-43	145.4	0.0	2.8e-43	145.0	0.0	1.1	1	Cyan7425_0928	alkyl hydroperoxide reductase/ Thiol specific antioxidant protein
2.6e-41	138.7	0.0	3e-41	138.5	0.0	1.0	1	Cyan7425_1511	alkyl hydroperoxide reductase/ Thiol specific antioxidant protein
5.6e-41	137.6	0.0	6.5e-41	137.4	0.0	1.0	1	Cyan7425_5136	alkyl hydroperoxide reductase/ Thiol specific antioxidant protein
1e-40	136.8	0.0	1.2e-40	136.6	0.0	1.1	1	Cyan7425_3716	alkyl hydroperoxide reductase/ Thiol specific antioxidant protein
4.7e-38	128.2	0.0	5.6e-38	127.9	0.0	1.1	1	Cyan7425_2289	alkyl hydroperoxide reductase/ Thiol specific antioxidant protein
3.5e-32	109.2	0.0	5.1e-32	108.7	0.0	1.3	1	Cyan7425_0215	Peroxidase
1.6e-27	94.2	0.0	2e-27	93.9	0.0	1.1	1	Cyan7425_4300	alkyl hydroperoxide reductase/ Thiol specific antioxidant protein
1.6e-22	78.0	0.0	2e-22	77.7	0.0	1.1	1	Cyan7425_0955	alkyl hydroperoxide reductase/ Thiol specific antioxidant protein
6.5e-17	59.9	0.0	8.3e-17	59.5	0.0	1.1	1	Cyan7425_1596	Peroxiredoxin
***Synechococcus* sp. WH 7803 (Complete)**									
6.2e-45	149.4	0.0	9.4e-45	148.8	0.0	1.3	1	SynWH7803_1118	Peroxiredoxin, AhpC/TSA family
4.3e-41	136.9	0.0	5e-41	136.7	0.0	1.0	1	SynWH7803_1042	Peroxiredoxin
1.1e-37	125.9	0.0	1.4e-37	125.6	0.0	1.1	1	SynWH7803_1236	Peroxiredoxin
2e-36	121.9	0.0	2.3e-36	121.7	0.0	1.0	1	SynWH7803_2172	Peroxiredoxin
***Synechococcus elongatus* PCC 7942 (Complete)**									
8.1e-44	145.8	0.0	9.4e-44	145.6	0.0	1.0	1	Synpcc7942_1942	bacterioferritin comigratory protein-like
5e-43	143.3	0.0	6.9e-43	142.8	0.0	1.2	1	Synpcc7942_2309	thioredoxin peroxidase
9.6e-40	132.7	0.0	1.1e-39	132.4	0.0	1.1	1	Synpcc7942_1806	bacterioferritin comigratory protein
2.7e-39	131.2	0.0	3.2e-39	131.0	0.0	1.1	1	Synpcc7942_0642	bacterioferritin comigratory protein
4.5e-38	127.3	0.0	5.2e-38	127.1	0.0	1.0	1	Synpcc7942_2180	bacterioferritin comigratory protein
3.9e-34	114.6	0.0	5e-34	114.2	0.0	1.1	1	Synpcc7942_2449	1-Cys peroxiredoxin
9.5e-20	68.1	0.0	1.3e-19	67.7	0.0	1.2	1	Synpcc7942_1730	hypothetical protein
***Synechococcus* sp. WH8102 (Complete)**									
1e-44	148.7	0.0	1.6e-44	148.0	0.0	1.3	1	SYNW1213	thioredoxin peroxidase
1.5e-38	128.7	0.0	1.8e-38	128.5	0.0	1.1	1	SYNW2162	putative bacterioferritin comigratory (BCP) protein
1.4e-35	119.1	0.0	1.6e-35	119.0	0.0	1.0	1	SYNW1015	putative bacterioferritin comigratory protein
5.3e-34	114.0	0.0	6.5e-34	113.8	0.0	1.1	1	SYNW1280	Alkyl hydroperoxide reductase/ Thiol specific antioxidant protein

***Cyanothece* sp. PCC 8801 (Complete)**									
7.7e-44	146.6	0.0	9.5e-44	146.3	0.0	1.1	1	PCC8801_3492	alkyl hydroperoxide reductase/ Thiol specific antioxidant protein
2e-43	145.3	0.0	2.4e-43	145.0	0.0	1.1	1	PCC8801_1115	alkyl hydroperoxide reductase/ Thiol specific antioxidant protein
4.1e-42	141.0	0.0	5.2e-42	140.7	0.0	1.1	1	PCC8801_1491	alkyl hydroperoxide reductase/ Thiol specific antioxidant protein
7.6e-42	140.2	0.1	8.7e-42	140.0	0.1	1.0	1	PCC8801_1631	alkyl hydroperoxide reductase/ Thiol specific antioxidant protein
2.5e-35	119.1	0.0	3.9e-35	118.5	0.0	1.3	1	PCC8801_4218	Peroxidase
1.6e-27	93.9	0.0	2e-27	93.6	0.0	1.1	1	PCC8801_3616	alkyl hydroperoxide reductase/ Thiol specific antioxidant protein
6.9e-24	82.1	0.1	8.6e-24	81.8	0.1	1.1	1	PCC8801_4240	alkyl hydroperoxide reductase/ Thiol specific antioxidant protein
***Prochlorococcus marinus* str. MIT 9211 (In complete)**									
2.6e-44	146.9	0.0	4e-44	146.3	0.0	1.3	1	P9211_09041	thioredoxin peroxidase
3.4e-38	127.1	0.0	4e-38	126.9	0.0	1.1	1	P9211_00881	putative bacterioferritin comigratory (BCP) protein
1.1e-36	122.2	0.0	1.4e-36	122.0	0.0	1.1	1	P9211_15101	Peroxiredoxin
1.1e-35	119.0	0.0	1.4e-35	118.7	0.0	1.1	1	P9211_08571	Alkyl hydroperoxide reductase/ Thiol specific antioxidant protein
***Prochlorococcus marinus* str. MIT 9215 (In complete)**									
1.6e-40	134.7	0.0	3.2e-40	133.8	0.0	1.4	2	P9215_10441	thioredoxin peroxidase
5.3e-37	123.4	0.0	6.3e-37	123.1	0.0	1.1	1	P9215_09891	Alkyl hydroperoxide reductase/ Thiol specific antioxidant protein
2.1e-36	121.5	0.1	2.4e-36	121.3	0.1	1.0	1	P9215_00921	putative bacterioferritin comigratory (BCP) protein
3.6e-33	111.0	0.0	4.2e-33	110.8	0.0	1.1	1	P9215_03801	putative bacterioferritin comigratory protein
***Prochlorococcus marinus* str. MIT 9301 (In complete)**									
1e-40	135.3	0.0	2e-40	134.4	0.0	1.5	2	P9301_10121	thioredoxin peroxidase
1.6e-36	121.8	0.0	1.9e-36	121.6	0.0	1.1	1	P9301_09561	Alkyl hydroperoxide reductase/ Thiol specific antioxidant protein
1.6e-36	121.8	0.1	1.9e-36	121.6	0.1	1.0	1	P9301_00911	putative bacterioferritin comigratory (BCP) protein
7.3e-33	110.0	0.0	8.5e-33	109.8	0.0	1.1	1	P9301_03801	putative bacterioferritin comigratory protein
***Prochlorococcus marinus* str. MIT 9303 (Complete)**									
3.8e-44	147.0	0.0	5.7e-44	146.5	0.0	1.3	1	P9303_14621	thioredoxin peroxidase
1.1e-38	129.5	0.0	1.3e-38	129.2	0.0	1.1	1	P9303_19011	putative bacterioferritin comigratory protein
1.5e-36	122.5	0.0	1.8e-36	122.3	0.0	1.1	1	P9303_02611	putative bacterioferritin comigratory (BCP) protein
4.2e-32	108.2	0.0	5.1e-32	107.9	0.0	1.1	1	P9303_10801	Alkyl hydroperoxide reductase/ Thiol specific antioxidant protein
***Synechococcus* sp. CC9311 (Complete)**									
2.5e-44	147.6	0.0	3.9e-44	146.9	0.0	1.3	1	sync_1322	thioredoxin peroxidase
4.6e-37	124.1	0.0	5.2e-37	123.9	0.0	1.0	1	sync_1556	bacterioferritin comigratory protein
7.7e-36	120.2	0.0	9.1e-36	119.9	0.0	1.1	1	sync_1400	Alkyl hydroperoxide reductase/ Thiol specific antioxidant protein
9e-36	119.9	0.0	1.1e-35	119.7	0.0	1.1	1	sync_2508	bacterioferritin comigratory protein
3e-15	53.7	0.0	4e-15	53.2	0.0	1.1	1	sync_0689	peroxiredoxin 2 family protein
2.3e-09	34.7	0.0	3e-09	34.3	0.0	1.2	1	sync_0254	Thioredoxin family protein
***Prochlorococcus marinus* MIT9312 (Complete)**									
6.7e-40	132.7	0.0	1.3e-39	131.8	0.0	1.5	2	PMT9312_0944	thioredoxin peroxidase
4.6e-37	123.6	0.0	5.5e-37	123.3	0.0	1.1	1	PMT9312_0897	alkyl hydroperoxide reductase/thiol specific antioxidant protein
4.2e-36	120.5	0.1	4.9e-36	120.3	0.1	1.0	1	PMT9312_0082	putative bacterioferritin comigratory (BCP) protein
1.3e-33	112.4	0.0	1.5e-33	112.2	0.0	1.1	1	PMT9312_0353	putative bacterioferritin comigratory protein
***Prochlorococcus marinus* MIT9313 (Complete)**									
2.4e-44	147.3	0.0	3.5e-44	146.8	0.0	1.3	1	PMT0754	thioredoxin peroxidase
1.2e-38	128.9	0.0	1.4e-38	128.6	0.0	1.1	1	PMT0387	putative bacterioferritin comigratory protein
8.5e-37	122.9	0.0	9.9e-37	122.7	0.0	1.1	1	PMT1622	putative bacterioferritin comigratory (BCP) protein
1.9e-32	108.9	0.0	2.3e-32	108.6	0.0	1.1	1	PMT1012	Alkyl hydroperoxide reductase/ Thiol specific antioxidant protein
8.2e-13	45.4	0.0	1.1e-12	45.0	0.0	1.2	1	PMT1186	Alkyl hydroperoxide reductase/ Thiol specific antioxidant protein
***Prochlorococcus marinus* str. MIT 9515 (In complete)**									
2.5e-41	137.3	0.1	4.8e-41	136.4	0.0	1.5	2	P9515_09331	thioredoxin peroxidase
3.5e-35	117.5	0.0	4.2e-35	117.2	0.0	1.1	1	P9515_09851	Alkyl hydroperoxide reductase/ Thiol specific antioxidant protein
4e-35	117.3	0.1	4.7e-35	117.0	0.1	1.0	1	P9515_00891	putative bacterioferritin comigratory (BCP) protein
2.7e-31	104.9	0.0	3.2e-31	104.7	0.0	1.1	1	P9515_03821	putative bacterioferritin comigratory protein
***Prochlorococcus marinus* str. AS9601 (Complete)**									
1.3e-40	135.0	0.0	2.5e-40	134.1	0.0	1.5	2	A9601_10131	thioredoxin peroxidase

4.3e-36	120.4	0.0	5.2e-36	120.1	0.0	1.1	1	A9601_09581 Alkyl hydroperoxide reductase/ Thiol specific antioxidant protein
8.4e-36	119.5	0.1	9.8e-36	119.3	0.0	1.0	1	A9601_00921 putative bacterioferritin comigratory (BCP) protein
4e-33	110.8	0.0	4.7e-33	110.6	0.0	1.1	1	A9601_03811 putative bacterioferritin comigratory protein
***Synechococcus* sp. CC9605 (Complete)**								
1.5e-43	145.0	0.0	2.4e-43	144.3	0.0	1.3	1	Syncc9605_1324 thioredoxin peroxidase
1.7e-38	128.6	0.0	2e-38	128.4	0.0	1.0	1	Syncc9605_2307 putative bacterioferritin comigratory (BCP) protein
5.4e-37	123.8	0.0	6.3e-37	123.6	0.0	1.0	1	Syncc9605_1141 putative bacterioferritin comigratory protein
7e-35	116.9	0.0	8.3e-35	116.7	0.0	1.1	1	Syncc9605_1406 Twin-arginine translocation pathway signal
1.2e-14	51.6	0.0	1.8e-14	51.0	0.0	1.2	1	Syncc9605_0215 conserved hypothetical protein
***Synechococcus* sp. CC9902 (Complete)**								
6.2e-45	149.2	0.0	9.6e-45	148.6	0.0	1.3	1	Syncc9902_1150 thioredoxin peroxidase
2.3e-38	128.0	0.0	2.7e-38	127.8	0.0	1.0	1	Syncc9902_0386 putative bacterioferritin comigratory (BCP) prptein
2.8e-38	127.7	0.0	3.3e-38	127.5	0.0	1.0	1	Syncc9902_1316 putative bacterioferritin comigratory protein
1.4e-36	122.2	0.0	1.7e-36	122.0	0.0	1.1	1	Syncc9902_1080 alkyl hydroperoxide reductase/thiol specific antioxidant protein
2.7e-11	40.5	0.0	4.2e-11	39.9	0.0	1.3	1	Syncc9902_0242 conserved hypothetical protein
***Acaryochloris marina* MBIC11017 (Complete)**								
9.7e-44	147.2	0.0	1.2e-43	146.9	0.0	1.1	1	AM1_2698 2-cys peroxiredoxin, putative
1.1e-41	140.6	0.0	1.3e-41	140.3	0.0	1.1	1	AM1_2940 alkyl hydroperoxide reductase/ Thiol specific antioxidant protein
1.1e-40	137.4	0.0	1.3e-40	137.1	0.0	1.1	1	AM1_1161 bacterioferritin comigratory protein
1.7e-39	133.4	0.0	2.2e-39	133.1	0.0	1.1	1	AM1_6007 Alkyl hydroperoxide reductase/Thiol specific antio
4.1e-38	129.0	0.0	4.8e-38	128.8	0.0	1.0	1	AM1_5436 bacterioferritin comigratory protein
8.9e-35	118.2	0.1	1.2e-34	117.9	0.0	1.2	1	AM1_A0300 peroxidase/ antioxidant protein
3.2e-32	110.0	0.0	4.4e-32	109.5	0.0	1.2	1	AM1_4173 redoxin domain protein, putative AhpC/TSA family
6.5e-30	102.5	0.1	8.4e-30	102.2	0.0	1.1	1	AM1_5494 Alkyl hydroperoxide reductase/Thiol specific antioxidant protein
5.7e-28	96.2	0.0	7e-28	96.0	0.0	1.1	1	AM1_2532 AhpC/TSA family protein
2.9e-21	74.6	0.0	4.7e-21	73.9	0.0	1.3	1	AM1_4765 redoxin domain protein, putative AhpC/TSA family
5.7e-17	60.7	0.0	6.8e-17	60.5	0.0	1.1	1	AM1_5336 redoxin domain protein, putative AhpC/TSA family
5.8e-15	54.2	0.0	7.6e-15	53.8	0.0	1.1	1	AM1_3680 peroxiredoxin, putative
***Nostoc punctiforme* ATCC 29133 (Complete)**								
3.5e-45	151.5	0.0	4.3e-45	151.2	0.0	1.1	1	Npun_F6082 alkyl hydroperoxide reductase/ Thiol specific antioxidant protein
5e-42	141.3	0.0	5.9e-42	141.1	0.0	1.1	1	Npun_F2872 alkyl hydroperoxide reductase/ Thiol specific antioxidant protein
8.9e-41	137.3	0.1	1e-40	137.1	0.1	1.0	1	Npun_R0493 alkyl hydroperoxide reductase/ Thiol specific antioxidant protein
3.8e-40	135.3	0.0	4.5e-40	135.0	0.0	1.1	1	Npun_R6477 alkyl hydroperoxide reductase/ Thiol specific antioxidant protein
4.9e-40	134.9	0.0	6e-40	134.6	0.0	1.1	1	Npun_F2425 alkyl hydroperoxide reductase/ Thiol specific antioxidant protein
6.1e-34	115.2	0.0	8e-34	114.8	0.0	1.2	1	Npun_F6498 alkyl hydroperoxide reductase/ Thiol specific antioxidant protein
2.7e-28	97.0	0.0	3.2e-28	96.7	0.0	1.1	1	Npun_F6083 alkyl hydroperoxide reductase/ Thiol specific antioxidant protein
8.4e-23	79.2	0.2	1.1e-22	78.8	0.1	1.2	1	Npun_F2528 alkyl hydroperoxide reductase/ Thiol specific antioxidant protein
9.6e-21	72.6	0.0	1.2e-20	72.3	0.0	1.1	1	Npun_F6135 alkyl hydroperoxide reductase/ Thiol specific antioxidant protein
8.5e-15	53.4	0.0	2.2e-13	48.8	0.0	2.1	2	Npun_R4657 glutaredoxin family protein
***Anabaena variabilis* ATCC 29413 (Complete)**								
4.6e-47	157.4	0.0	5.8e-47	157.0	0.0	1.1	1	Ava_2024 Alkyl hydroperoxide reductase/ Thiol specific antioxidant protein
6.3e-43	144.0	0.0	7.6e-43	143.7	0.0	1.1	1	Ava_3881 Alkyl hydroperoxide reductase/ Thiol specific antioxidant protein
8.5e-42	140.4	0.1	9.8e-42	140.2	0.0	1.0	1	Ava_0194 Alkyl hydroperoxide reductase/ Thiol specific antioxidant protein
1.2e-41	139.9	0.0	1.4e-41	139.7	0.0	1.1	1	Ava_0435 Alkyl hydroperoxide reductase/ Thiol specific antioxidant protein
8.4e-41	137.1	0.0	1e-40	136.9	0.0	1.1	1	Ava_0485 Alkyl hydroperoxide reductase/ Thiol specific antioxidant protein
4.7e-33	112.1	0.0	6.6e-33	111.6	0.0	1.2	1	Ava_1358 1-Cys peroxiredoxin
5.6e-27	92.5	0.0	7.4e-27	92.1	0.0	1.1	1	Ava_2023 putative thiol-specific antioxidant protein
1.4e-22	78.2	0.0	1.8e-22	77.9	0.0	1.1	1	Ava_2915 hypothetical protein
5.8e-20	69.8	0.0	7.1e-20	69.5	0.0	1.1	1	Ava_0627 hypothetical protein
***Cyanothece* sp. ATCC 51142 (Complete)**								
1.8e-42	142.5	0.0	2.2e-42	142.1	0.0	1.1	1	cce_2409 thioredoxin peroxidase
2.1e-42	142.2	0.0	2.5e-42	142.0	0.0	1.1	1	cce_1296 bacterioferritin comigratory protein
4.3e-42	141.2	0.1	4.9e-42	141.0	0.1	1.0	1	cce_3358 putative bacterioferritin comigratory protein

9.4e-36	120.7	0.0	1.2e-35	120.3	0.0	1.2	1	cce_0135	putative rehydrin
1.7e-25	87.6	0.0	2.1e-25	87.3	0.0	1.1	1	cce_4124	putative thiol-specific antioxidant protein
1.4e-24	84.7	0.0	2.1e-24	84.1	0.0	1.3	1	cce_3631	hypothetical protein
1.7e-16	58.6	0.0	2.2e-16	58.2	0.0	1.1	1	cce_3126	peroxiredoxin
***Thermosynechococcus elongatus* BP-1 (Complete)**									
1e-44	148.6	0.0	1.3e-44	148.3	0.0	1.1	1	tll1454	thioredoxin peroxidase
1.3e-43	145.1	0.0	1.5e-43	144.8	0.0	1.1	1	tlr1194	bacterioferritin comigratory protein
1.3e-42	141.8	0.0	1.6e-42	141.6	0.0	1.0	1	tlr1198	bacterioferritin comigratory protein
6.1e-40	133.2	0.0	7e-40	133.0	0.0	1.0	1	tll1451	bacterioferritin comigratory protein homolog
4.1e-33	111.1	0.0	9e-33	110.0	0.0	1.5	2	tlr2261	AhpC/TSA family protein
3.1e-25	85.7	0.0	3.9e-25	85.4	0.0	1.1	1	tlr1788	hypothetical protein
1.5e-22	77.1	0.0	1.9e-22	76.7	0.0	1.1	1	tlr1289	hypothetical protein
***Prochlorococcus marinus* MED4 (In complete)**									
5.6e-41	136.0	0.1	1e-40	135.2	0.0	1.5	2	PMM0856	thioredoxin peroxidase
2.4e-35	117.9	0.1	2.7e-35	117.6	0.1	1.0	1	PMM0079	putative bacterioferritin comigratory (BCP) protein
1.1e-34	115.8	0.0	1.3e-34	115.5	0.0	1.1	1	PMM0903	Alkyl hydroperoxide reductase/ Thiol specific antioxidant protein
2.4e-33	111.4	0.0	2.8e-33	111.2	0.0	1.1	1	PMM0345	putative bacterioferritin comigratory protein
3.6e-10	36.5	0.0	5.4e-10	35.9	0.0	1.2	1	PMM0283	conserved hypothetical protein
