# Supplementary figures and images for: Genome-wide analysis of putative peroxiredoxin in unicellular and filamentous cyanobacteria
Source: BMC Evol Biol. 2012 Nov 16;12:220. doi: 10.1186/1471-2148-12-220 (PMC3514251; doi:10.1186/1471-2148-12-220)

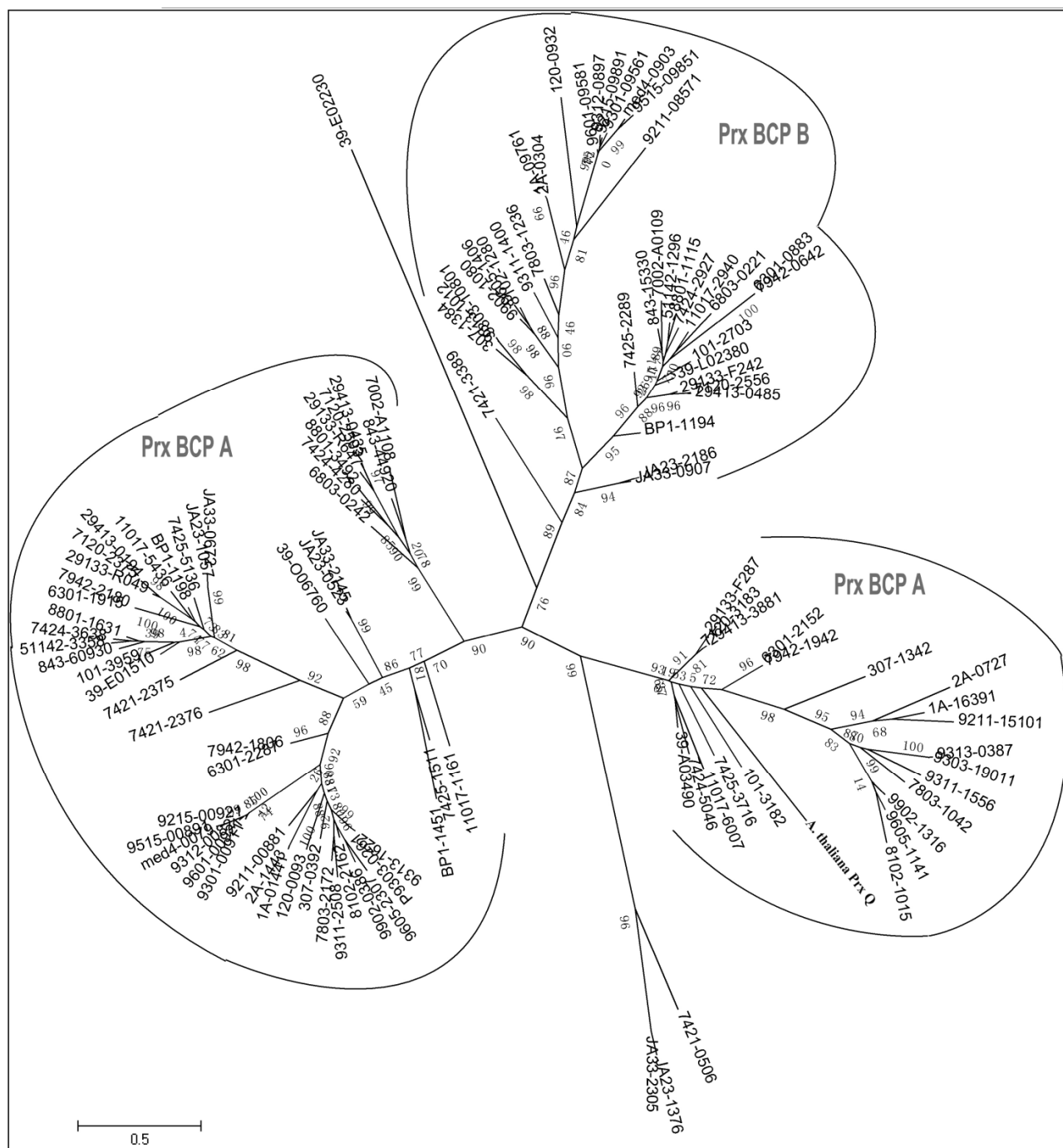

Supplement: Additional file 2 — Figure S1. Maximum likelihood tree of PRXs from Prx-BCP subfamily. [file 1471-2148-12-220-S2.pdf]

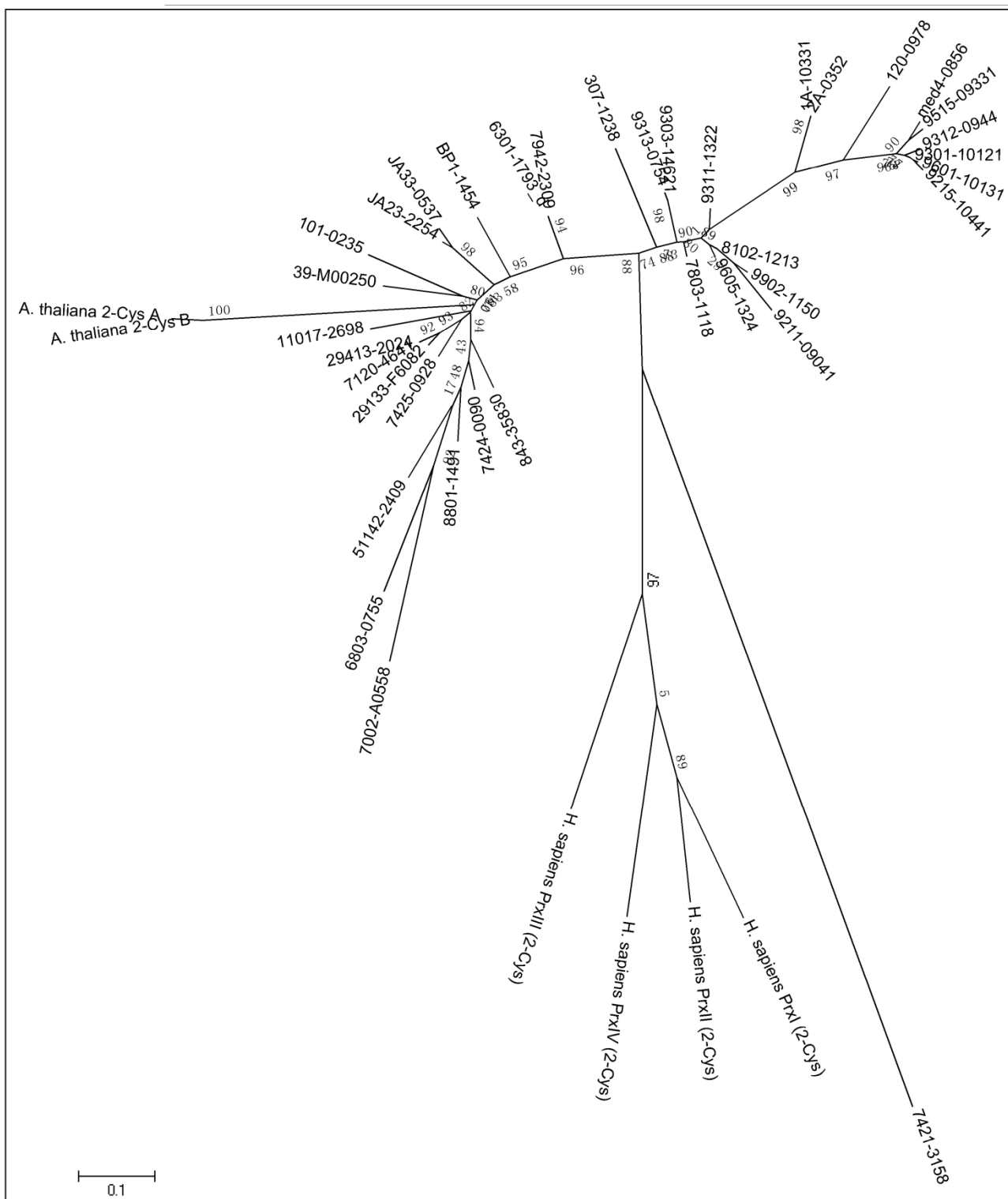

Supplement: Additional file 3 — Figure S2. Maximum likelihood tree of PRXs from 2-Cys subfamily. [file 1471-2148-12-220-S3.pdf]

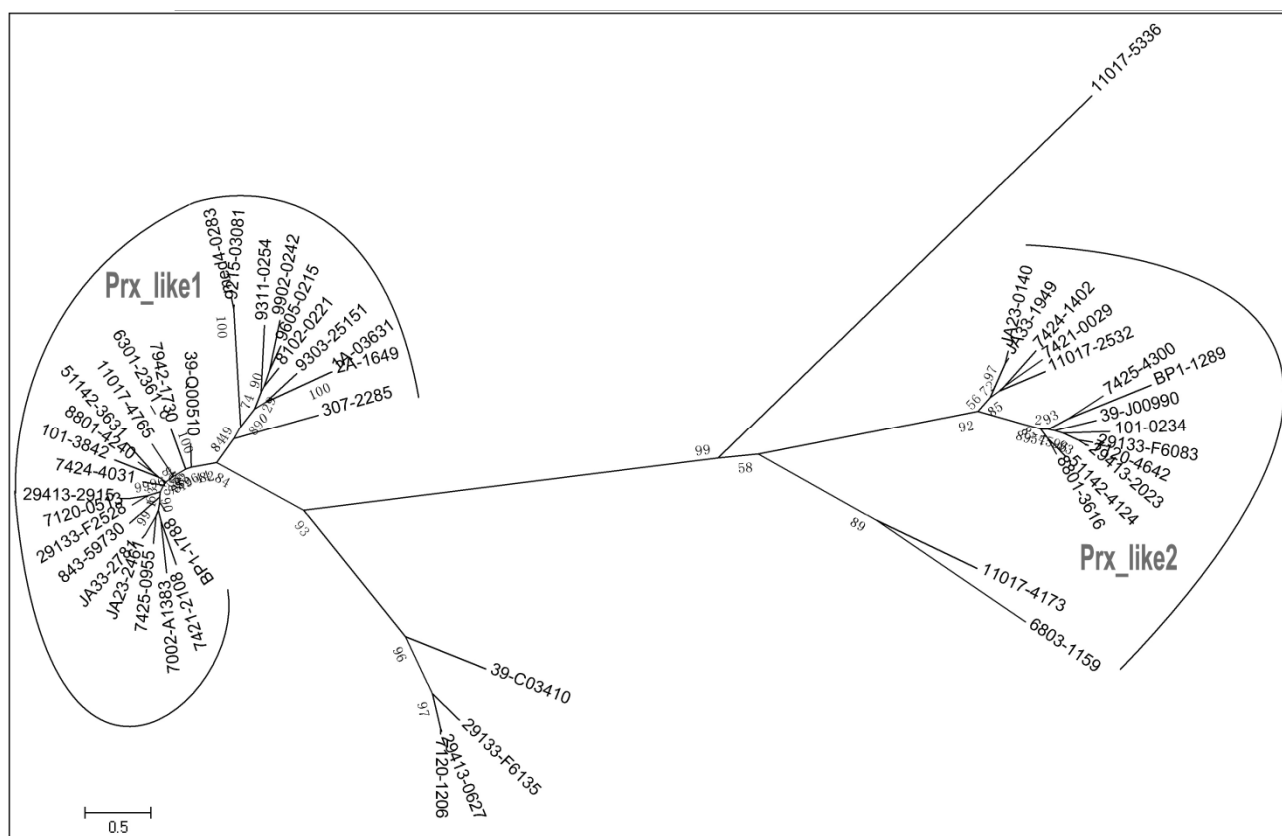

Supplement: Additional file 4 — Figure S3. Maximum likelihood tree of PRXs from Prx-like subfamily. [file 1471-2148-12-220-S4.pdf]

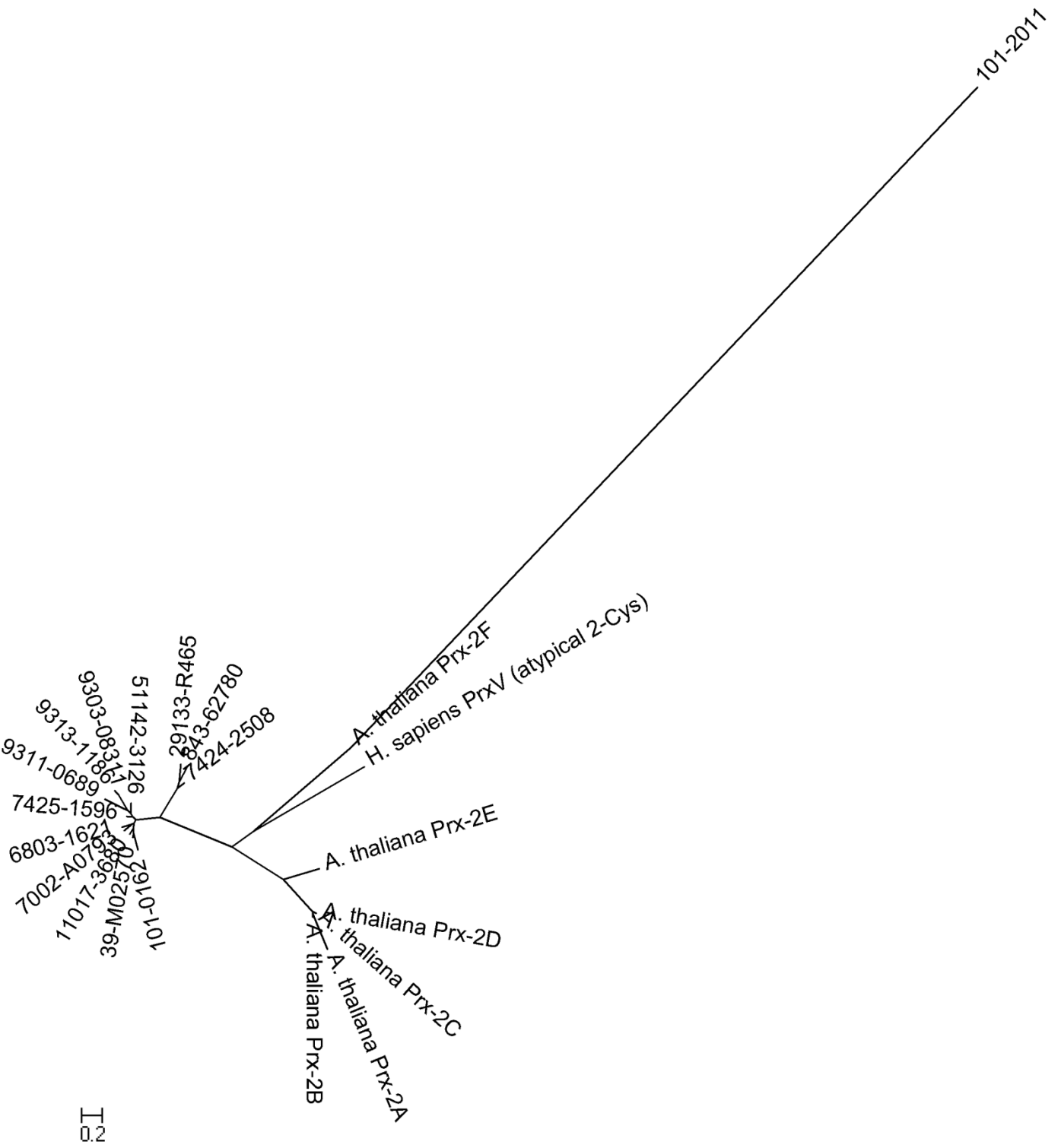

Supplement: Additional file 5 — Figure S4. Maximum likelihood tree of PRXs from Prx5_like subfamily. [file 1471-2148-12-220-S5.pdf]

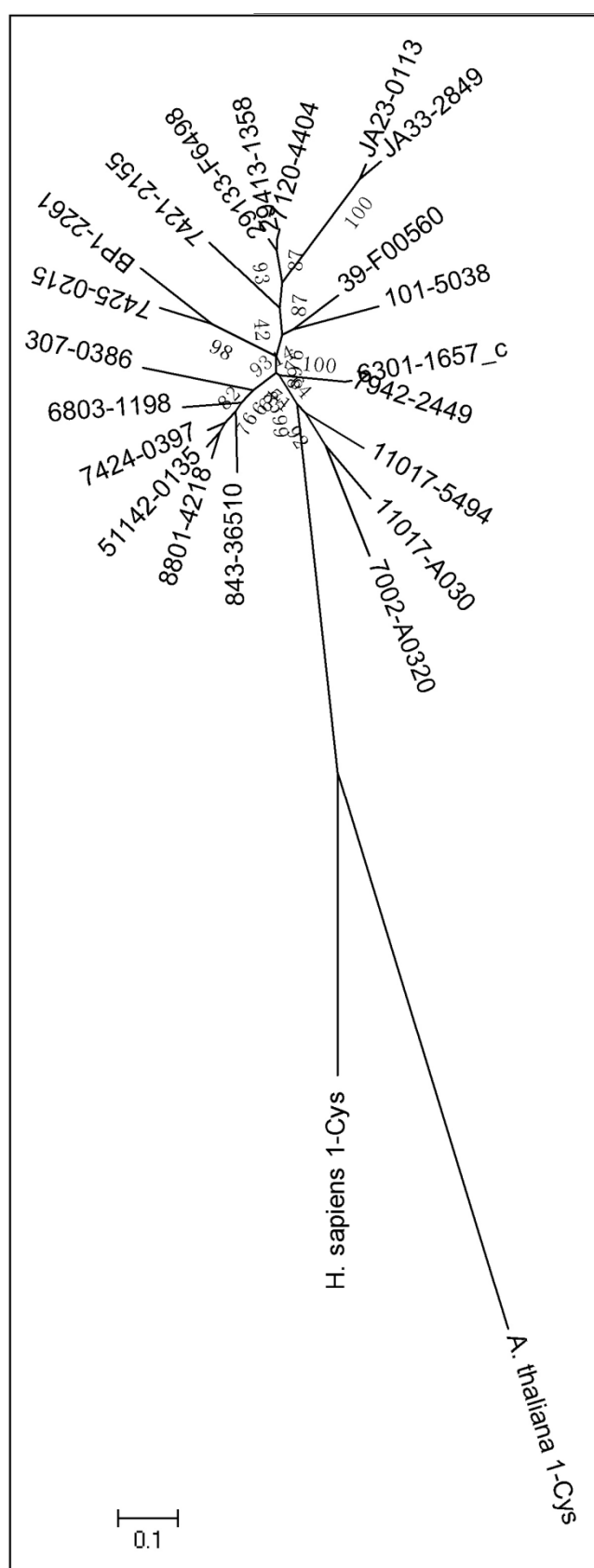

Supplement: Additional file 6 — Figure S5. Maximum likelihood tree of PRXs from 1-Cys subfamily. [file 1471-2148-12-220-S6.pdf]
