# Supplementary material for: Genome-wide analysis of putative peroxiredoxin in unicellular and filamentous cyanobacteria
Source: BMC Evol Biol. 2012 Nov 16;12:220. doi: 10.1186/1471-2148-12-220 (PMC3514251; doi:10.1186/1471-2148-12-220)
Supplement: Additional file 7 — The translated protein sequences of 244 putative PRXs in 37 cyanobacteria. [file 1471-2148-12-220-S7.pdf]

>ara-1 Arabidopsis thaliana 1-cys
MPGITLGDTVPNLEVETTHDKFKLHDYFANSWTVLFSHPGDFTPVCTTELGAMAKYAHEFDKRGVKLLGLSCD
DVQSHKDWIKDIEAFNHGSKVNYPIIADPNKEIIPQLNMIDPIENGPSRALHIVGPDSKIKLSFLYPSTTGRNMDV
LRALDSLLMASKHNNKIATPVNWKPDQPVVISPAVSDEEAKKMFPQGFKTADLPSKKGYLRHTEVS
>ara-2 Arabidopsis thaliana 2-Cys peroxiredoxin A
MASVASSTTLISSPSSRVFPAKSSLSSPSVSFLRTLSSPSASASLRSGFARRSSLSSTSRRSFAVKAQADDLPLVGNK
APDFEAEAVFDQEFIKVKLSDYIGKKYVILFFYPLDFTFVCPTEITAFSDRHSEFEKLNTEVLGVSVDSVFSHLAWV
QTDRKSGGLGDLNYPLISDVTKSISKSFGVLIHDQGIALRGLFIIDKEGVIQHSTINNLGIGRSVDETMRTLQALQYI
QENPDEVCPAGWKPGEKSMKPDPKLSKEYFSAI
>ara-3 Arabidopsis thaliana 2-Cys peroxiredoxin B
MSMASIASSSSTTLLSSSRVLLPSKSSLLSPTVSFPRIIPSSSASSSSLCSGFSSLGSLTTNRSASRRNFAVKAQADDP
LVGNKAPDFEAEAVFDQEFIKVKLSEYIGKKYVILFFYPLDFTFVCPTEITAFSDRYEEFEKLNTEVLGVSVDSVFSHL
AWVQTDRKSGGLGDLNYPLVSDITKSISKSFGVLIPDQGIALRGLFIIDKEGVIQHSTINNLGIGRSVDETMRTLAL
QYVQENPDEVCPAGWKPGEKSMKPDPKLSKEYFSAI
>ara-4 Arabidopsis thaliana peroxiredoxin Q
MAASSSSFTLCNHTTLRTLPLRKTLVTKTQFSVPTKSSESNFFGSTLTHSSYISPVSSSSLKGLIFAKVNKGQAAPDF
TLKDQNGKPVSLKKYKGKPVVLYFYPADETPGCTKQACAFRDSYEKFKKAGAEVIGISGDDSASHKAFASKYKLP
YTLLSDEGNKVRKDWGVPGDLFGALPGRQTYVLDKNGVVQLIYNNQFQPEKHIDETLKFLKAA
>ara-6 Arabidopsis thaliana peroxiredoxin-2B
MAPIAVGDVVPDGTISFFDENDQLQTVSVHSLAAGKKVILFGVPGAFTPTCSMKHVPGFIEKAEELKSKGVDDII
CFSVNDPFLMKAWGKTYPENKHVKFVADGSGEYTHLLGLELDLKDKGLGIRSRRFALLLDNLKVTVANLESGGG
FTVSSADDILEAL
>ara-7 Arabidopsis thaliana peroxiredoxin-2F
MAMSILKLRNLSALRSAANSARIGVSSRGFSKLAEGTDITSAAPGVSLQKARSWDEGVSSKFSTTPLSDIFKGKKV
VIFGLPGAYTGVCSQQHVPSYKSHIDKFKAKGIDSVICVSVNDPFAINGWAEKLGAKDAIEFYGDFDGKFHKSLG
LDKDLSAALLGPRSERWSAYVEDGKVKAVNVEEAPSDFKVTGAEVILGQI
>ara-8 Arabidopsis thaliana peroxiredoxin-2A
MAPIDVGDFVPDGSISFFDDDDQLQTVSVHSLAAGKKVILFGVPGAFPPTCSMNHVNGFIEKAEELKSNGVDEI
ICLSGDDPFMITACSENKHVKFVEDGSGEYIQLLGLELEVKDKGLGVRSRGFALLLDNLKVIVVNVGSGGDCSLF
QLMKMTTTTMSNLPTDLLEEIISRVPRKYMRAVRLTCKRWNGMFKSQSFTKMHIGKEEAATRELRQTRMIVM
MDYN
>ara-9 Arabidopsis thaliana peroxiredoxin-2D
MAPITVGDVVPDGTISFFDENDQLQTVSVHSIAAGKKVILFGVPGAFTPTCSMSHVPGFIGKAEELKSKGIDEIIC
FSVNDPFVMKAWGKTYQENKHVKFVADGSGEYTHLLGLELDLKDKGLGIRSRRFALLLDNLKVTVANVENGGE
FTVSSAEDILKAL
>ara-10 Arabidopsis thaliana peroxiredoxin-2E
MATSLSVSRFMSSSATVISVAKPLLSPTVSFTAPLSFTRSLAPNLSLKFRNRRTNSASATTRSFATTPVTASISVGDK
LPDSTLSYLDPSTGDVKTVTVSSLTAGKKTILFAVPGAFTPTCSQKHVPGFVSKAGELRSKGIDVIACISVNDAFVM
EAWRKDLGINDEVMLLSDGNGEFTGKLGVELDLRDKPVGLGVRSRRYAILADDGV
VKVLNLEEGGAFTNSSAEDMLKAL
>ara-11 Arabidopsis thaliana peroxiredoxin-2C
MAPITVGDVVPDGTISFFDENDQLQTVSVHSIAAGKKVILFGVPGAFTPTCSMSHVPGFIGKAEELKSKGIDEIIC
FSVNDPFVMKAWGKTYPENKHVKFVADGSGEYTHLLGLELDLKDKGLGIRSRRFALLLDNLKVTVANVESGGEF
TVSSAEDILKAL

>ara-12 Homo sapiens PrxI (2-Cys)
MSSGNAKIGHPAPNFKATAVMPDGQFKDISLSDYKGKYVVFFFYPLDFTFVCPTEIIAFSDRAEEFKKLNCQVIGA
SVDSHFCHLAWVNTPKKQGGLGPMNIPLVSDPKRTIAQDYGVLKADEGISFRGLFIIDDKGILRQITVNDPPCCR
SVDETLRLVQAFQFTDKHGEVCPAGWKPGSDTIKPDVPKTKEYFSKQK
>ara-13 Homo sapiens PrxII (2-Cys)
MASGNARIGKPAPDFKATAVVDGAFKEVKLSDYKGKYVVLFFYPLDFTFVCPTEIIAFSNRAEDFRKLGCEVLGVS
VDSQFNHLAWINTPRKEGGLGPLNIPLLGDVTRRLSEDYGVLKTDEGIAYRGLFIIDGKGVLRQITVNDLPVGRSV
DEALRLVQAFQYTDEHGEVCPAGWKPGSDTIKPNVDDSKEYFSKHN
>ara-14 Homo sapiens PrxIII (2-Cys)
MAAAVGRLLRASVARHVSAIPWGISATAALRPAACGRTSLTNLLCSGSSQAKLFSTSSSCHAPAVTQHAPYFKGT
AVVNGEFKDLSLDDFKGKYLVLFFYPLDFTFVCPTEIVAFSDKANEFHDVNCEVVAVSVDSHFSHLAWINTPRKN
GGLGHMNIALLSDLTKQISRDYGVLLEGSGLALRGLFIIDPNGVIKHLSVNDLPVGRSVEETLRLVKAFQYVETHG
EVCPANWTPDSPTIKPSPAASKEYFQKVNQ
>ara-15 Homo sapiens PrxIV (2-Cys)
MEALPLLAATTPDHGRHRRLLLLPLLLFLLPAGAVQGWETEERPRTREEECHFYAGGQVYPGEASRVSVADHSL
HLSKAKISKPAPYWEGTAVIDGEFKELKLTDYRGKYLVFFFYPLDFTFVCPTEIIAFGDRLEEFRSINTEVVACSVDS
QFTHLAWINTPRRQGGLGPIRIPLLSDLTHQISKDYGVYLEDSGHTLRGLFIIDDKGILRQITLNDLPVGRSVDETL
RLVQAFQYTDKHGEVCPAGWKPGSETIIPDPAGKLKYFDKLN
>ara-16 Homo sapiens PrxV (atypical 2-Cys)
MGLAGVCALRRSAGYILVGGAGGQSAAAAARRCSEGEWASGGVRSFSRAAAAMAPIKVGDAIPAVEVFEGEP
GNKVNLAELFKGKKGVLFGVPGAFTPGCSKTHLPGFVEQAEALKAKGVQVVACLSVNDAFVTGEWGRAHKAE
GKVRLLADPTGAFGKETDLLLDDSLVSIFGNRRLKRFSMVVQDGIVKALNVEPDGTGLTCSLAPNIISQL
>ara-17 Homo sapiens PrxVI (1-Cys)
MPGGLLLGDVAPNFEANTTVGRIRFHDFLGDSWGILFSHPRDFTPVCTTELGRAAKLAPEFAKRNVKLIALSIDS
VEDHLAWSKDINAYNCEEPTEKLPFPIIDDRNRELAILLGMLDPAEKDEKGMPVTARVVFVFGPDKKLKLSILYPA
TTGRNFDEILRVVISLQLTAEKRVATPVDWKDGDSVMVLPTIPEEEAKKLFPKGVFTKELPSGKKYLRYTPQP
>11017-2940
MKPLRHHLTRICLVLGLIVSLVMAAPAAFAMGGDPPPLDQPAPTFTLPSNTGDGEVSLSDYRGQWVVLYFYPQ
DFTPGCTVEAQRFQQDLPKYIERNTQVIGVSVDSVDSHAEFCDSEGLKFPLLADEDGSVSKAYGSWMSYFSLRH
TYLIDPEGILREQYVKVRPVIHSEEVLARLDELQAAS
>11017-1161
MTLDIGDYAPEFTLPNADGESIDLKRFRGQWVVLYFYPRDNTPGCTKEACGFRDQYETYQTEQVVILGVSGDD
AKSHQKFINKQNLPFQLLSDLDFNVAKSYEAYGPKKFMGKEYEGIYRHSFLIDPDGKLAKIYRKVKAADHATDVL
QDLELLRK
>11017-5436
MALTAGTAAPAFTTKDTNGNTVSLADYAGKTVVLYFYPKDDTPGCTKEACSFRDNYTAYQGKDIVVFGVSGDDE
GSHQDFTSKFNLPFPLLADVDHSLMKAYDVDGGGYAQRVTYVINGQGQIDKVYTSVNTDTHATDILADLGL
>11017-6007
MVHPLGLYNWDEISLIALGEEQKSHIQTKSMAIKVGNSVPDVTLQTPTGETVSLKNFLGEKIVVLYFYPKDDTPG
CTKEACGFRDSYTVFQEAGAEVIGVSNDTPNSHQQFANKYQLPFTLLSDGANQARKAFGVPSTLGLLPGRVTY
VIDTDGVVRHIFNSQLNFQGHIDESLKIVQSLTKDA
>11017-2698
MSQHEAHECLRVGQAAPDFTATAVYDQEFSEVKLSNYRGKYVVIFFYPLDFTFVCPTEITAFSDRYDAFKDLNTE
VLGISVDSEFSHLAWTQTDRKSGGVGDLNYPLVSDIKKEISTAYNVLDPDAGVALRGLFIIDKEGVVQHATINNLA

FGRNVDETLRTLQAIQHVQSHPDEVCPAGWQPGDKTMNPDPVKSKVYFESV
>11017-A0300
MTLQLGDTVPNFTQKTTEGDLNFYDWAGDSWVVLFSHPADYTPVCTTELGSVAKLKSEFASRNAKVIALSVDD
VNSHKGWINDINETQNTVVNYPIIADADQTVANLYGMIHPNANAKVTVRTVFVIDPDKKLRLTLTYPPSTGRNN
EILRVLDSLQLTDNYSVATPVDWTDGDDVVVAPSISTADAKQKFPKGVNEIKPYLRMTPQPNR
>11017-4173
MSLATELQAVTENVRQQAPENVFTTMEAATAKLAATGITDQALQTGQTMPDFELPDATGKSVSSSELRAKGLLL
ISFYRGNWCPYCNLELQALQARLDDIAALGATLVAISPESPDQSLTTQEKFDLKFPVLTDTGNQVARQFGLVFTLD
ESLRPIYNNFGIDITTHNGDQSFELPVPATYLVAADGTVLNHFVDVDYRERLAPETALAWLQAAQ
>11017-5494
MSLRLGDTVPNFTQDSTAGTIDFFEWAGDSWVVLFSHPADYTPVCTTELGSVSKLKPEFDKRHVKTIALSVDDA
QAHNGWIGDINETQNTTVDYPILADADKKVSDLYDMIHPNANAKVTVRTVFIIDNNKKLRASITYPPSTGRNFN
EILRVIDSLQLTDNYQVATPVDWQDGDDVVVAPTIPTEEAKQKFPKGVTEIKPYLRMTPQPNK
>11017-2532
MTQSSGFFNQRYVNNFVPLPGKGEPAVGRLAPDFELPRIGGDSVKLSDYRGQQPIFLAFTRIFTEKLFCPFCYPHI
QDLKQRYSEIVDRGAELLMISSTDQVQSEQIVSDLDLPYPFLYNPKCESFRNYGAGQALGAPLPAQYIVDVDGTI
RFRHMFSFVDHNAGMDEVLTILEGLIPQG
>11017-5336
MMTFAQSTSPKLLMGHTAPLLVVQTLDHGLWRLADQTPEHYTMVVFYRGLHCPVCEQYLTELDQKLSAFAQL
GVQVIALSGDGIDKTQQLKTQANLQQLSLGYGLTPEQMRDWGLYLSQGHFEQEPALFSEPAVFLIQPDGRLYFA
NIGTHPFSRIDFDSLLAGLAYVIPNNYPLRGTA
>11017-4765
MVLTASTMLDLGTPAPNFQLPDVVSGQTISLETFAHRNVLLVMFICQHCPFVKHVQAELAQIGHDYSDQSLGIV
AISSNDIQSHPLDDPEHLTAMATSLGYNFPICFDEHQDVAKQYTAACTPDFFVFDAHRKLVYRGQLDDSRPSNN
LMVTGEDLRGALDAALAEQPISANQKPSIGCNIKWKPGNEPAYYG
>11017-3680
MAVFATERVPDVVFKTRVRDESVPGPNPYRWQDKTTQDIFGGKRVVLFSLPGAFTPTCSSTHLPRYEELHEEIK
AQGVDEIICLSVNDAFVMFQWGKHQGADKVFLLPDGNGEFTRKMGMLVDKSNLGFGMRSWRYSMVVNN
GQIEKMFIEADYGDNCPTDPFEVSDADTMLAYLKGVHATAAPTAAAVG
>11017-3814
MSFFQPQRRKLLSLLGLGAASAVGAALLPRSQNRSVTAFETPSNTSLWTKRDDVNLGQPLHPFQGISHWLNSE
PLSIADLKGKVVLVQFWTFGCINSQRTLPYVTRWHQQYADQGLQVIGIHTPEFKYEHAASNVQKALAEYQIAYP
VPLDNNYQTWKAYRNRYWPHLFLTNREGVITYHHIGEGAYQETEQTIQALLG
>7120-2556
MISRRNFLHILLVSCFAVISWLNLPPTAYALGGKLPPINQPAPDFTLPTNTGDGKLSLGDLRGKWVVLYFYPKDFT
SGCTIEARRFQQDLPTYLDKNVQIIGVSADDIDSHAEFCDSEGLKFPLLADTDGAVSKAYGSWIGYVSMRHSFIID
PQGILRETFVKVNPSVHSTEVLARLEKLQSTAS
>7120-2503
MSNIPQAGQPAPDFSTPDQNGNLVSLNDFQGQWVVIYFYPKDDTPGCTTEAKDFTGLHQEFNQLGAKILGVS
PDSGKAHCKFIDKHNLSINLLSDPEHQLIEAYGAWRLKKFMGKEYMGVARSTFLISTDGIIAYAWPNVKTKGHA
QAVLNKLQELINS
>7120-2375
MPLAVGTDAPAFTVKDTNGNTVSLSDFAGKTVVLYFYPKDDTPGCTKQACSFRDAQSDYKNKDVVVLGVSAD
DEGSHQAFTQKYNLNFPLLADTNKTLISAYDVDGGGYAKRVTYVIGPDGKIVHVDASVNTTTHAGDVLAALGL

>7120-3183
MPVKVGDSAPDFTLPAQNGSSVSLSDFRGKKAVVLYFYPKDDTPGCTAESCAFRDRYEVFQTAGAEIIGVSGDS
NESHQKFASKYNLPFTLLSDKGDQVRKLYGATAAFGLFPGRVTYVIDQQGVVQYVFDSMFNFQGHVEEALKTL
QQLASK
>7120-4641
MSITYGTQESLRVGQQAPDFTATAVVDQEFKTIKLSDYRGKYVVLFFYPLDFTFVCPTEITAFSDRYEEFKKLNTEIL
GVSVDSEFSHLAWIQTDRKSGGVGDLNYPLVSDIKKEVSDAYNVLDPAAGIALRGLFIIDKDGIIQHATINNLAFG
RSVDETLRTLQAIQYVQSHPDEVCPAGWQPGEKTMTPDPVKSKVYFAAV
>7120-4404
MALRLGDTVPNFTQASTHGDIDFYAWAGDSWVVLFSHPADYTPVCTTELGTVAKLKPEFDKRNVKAIALSVDD
VESHNGWVGDIEETQSTTLNYPILADADRKVSDLYDMIHPNANAAVTVRSVFVIDPNKKLRLTFTYPPSTGRNF
DELLRVIDSLQLTDNYSVATPADWKDGDKVVIVPSLKDPEVLKEKFPKGYEVVKPYLRLTPQPNK
>7120-4642
MNADRHRYKISVNSGVHLWFYHCIYYQLVDNFERFMLTSTDFSGLLNERFFRNFLPIPASNELRLDVGTPDFQLP
DITNGTLVKLSNYRGKQPILLAFTRIFTEKQYCPFCFPHIKALNENYEQFTNRGIEVLLVTSTDEKQSQIVVKDLGLK
MPLLSDPSCRAFRTYQVGQALGAPLPAQFVLDKDGRLRYKHLFSFFDHNASVEKLLGKFD
>7120-0513
MALTASTMVPIGTQAPDFHLPDVVSGKTISLSTFADKKALLVMFICRHCPFVKHIQDELTRIGQDYSTSDLGIVAIS
ANDAKNYPDDAPESLKALAIELGWQFPFCYDETQETAKAYTAACTPDFFVFDSDRQLAYRGQLDDSRPSNGKP
VTGADLRAAIDAVLAGKPVIGEQKPSIGCNIKWKPTP
>7120-1206
MTILETSNTPVGGYAPDFELPGIDNQVHHLSRYLDNFRAVGVVSLGNYCSYVNLYLDRLKGIQGEFGTDGFILIGI
NASDITEPSWSSLEKMKAFAQNHELNFPYLWDSTQEVSRSFGATKTPIAFLIDSHGILRYRGQIDNHPQEPSSVG
EDYLRNAIAALFKGEEILVPETEPVGTSLIWRI
>29413-0485
MISRRNFLHILLVSCFAVISWLNFAPTAYALGGKLPAINQPAPDFTLPTNTGDGKLSLADFRGKWVVLYFYPKDFT
SGCTIEARRFQQDLPTYVDKNVQIIGVSADDIDSHAEFCDSEGLKFPLLADTDGAVSKAYGSWIGFVSMRHSFII
DPQGILRETFVKVNPSVHSTEVLARLEKLQSAAS
>29413-0435
MSNFPQAGQPAPDFSTPDQNGNLVSLNDFHGQWVVIYFYPKDDTPGCTTEAKDFTGLHEEFHRLGAKILGVS
PDSGKAHCKFIDKHNLSITLLSDPEHQLIEAYGAWRLKKFMGKEYMGVARSTFLISTDGIIAYTWPNVKTKGHAQ
AVLNKLQELTNS
>29413-0194
MPLAVGTDAPAFTVKDTNGNTVSLSDFAGKTVVLYFYPKDDTPGCTKQACSFRDAQSDYKNKDVVVLGVSAD
DEGSHQAFTQKYNLNFPLLADTNKTLISAYDVDGGGYAKRVTYVIGPDGKIVHVDASVNTTTHASDVLAALGL
>29413-3881
MPVKVGDSAPDFTLPAQNGSSVSLSDFRGKKAVVLYFYPKDDTPGCTAESCAFRDRYEVFQTAGAEIIGVSGDS
NESHQKFASKYNLPFTLLSDKGDQVRKLYGATAAFGLFPGRVTYVIDQQGVIKYVFDSMFNFQGHVEEALKTLQ
QLANK
>29413-2024
MSITYGTQESLRVGQQAPDFTATAVVDQEFKTIKLSDYRGKYVVLFFYPLDFTFVCPTEITAFSDRYEEFKKLNTEIL
GVSVDSEFSHLAWIQTDRKSGGVGDLNYPLVSDIKKEVSDAYNVLDPAAGIALRGLFIIDKDGIIQHATINNLAFG
RSVDETLRTLQAIQYVQSHPDEVCPAGWQPGEKTMTPDPVKSKVYFAAV
>29413-1358

MALRLGDTVPNFTQASTHGDIDFYEWAGDSWVVLFSHPADYTPVCTTELGTVAKLKPEFDKRNVKAIALSVDD
VESHKGWVGDIEETQSTTLNYPILADADRKVSDLYDMIHPNANAAVTVRSVFVIDPNKKLRLSFTYPPSTGRNF
DELLRVIDSLQLTDNYSVATPADWKDGDKVVIVPSLKDPEVLKEKFPKGYEVVKPYLRLTPQPNK
>29413-2023
MLTSTDFSGLLNERFFRNFLPIPASNELRLDVGTPDFQLPDITNGTLVKLSNYRGKQPVLLAFTRIFTEKQYCPFCF
PHIKALNENYEQFTNRGIEVLLVTSTDEKQSQIVVKDLSLKMPLLSDPSCRTFRTYQVGQALGAPLPAQFVLDKD
GRLRYKHLFSFFDHNASIEKLLEKFD
>29413-2915
MALTASIMVPIGTQAPDFHLPDVVSGKTISLSTFADKKALLVMFICRHCPFVKHIQDELTRIGQDYATRDLGIVAIS
ANDAENYPDDAPESLKALAIELGWQFPFCYDETQETAKAYTAACTPDFFVFDDHRHLAYRGQLDDSRPSNGKP
VTGADLRAAIDAVLAGKPVIDEQKPSVGCNIKWKSTP
>29413-0627
MTILETSNTPVGGYAPDFELPGIDNHVHHLSRYLDNFRAVGVVSLGNHCPYVNLYLDRLKAIQGEFGAEGFILIGI
NASDITEPSWSSLEKMKAFAQNHKLNFPYLWDSTQEVSRSFGATKTPIAFLIDSHGILRYRGQIDNHPQEPSSVG
EDYLRNAIAALFKGEEIPVPETEPVGTSLIWHI
>39-L02380
MLRRTLISLIVGIAIALFNLNLASPVLALGGQLPPLNEPAPEFTLPTNTGDGEVSLSDYRGQWVVVYFYPKDFTPG
CTLEARRFQEDLPKYMARNTQILGISADDVDSHAEFCDSEGLKFPLLADVDGSVSKAYGSWLNFFSMRHSFIIDP
DGILRETFVGVIPAIHSQEVLARLDEMINS
>39-E01510
MALSAGTKAPAFTAKDTNGNTVSLSDFAGKTVVLYFYPKDDTPGCTKEAQSFRDNYENYQSRDMVVLGVSTD
DEASHKLFTEKYGLPFQLLADTDGAITKAYDVDGGGYAKRVTYIINGEGVIDHVDDKVNTSTHAQDILAKVN
>39-O06760
MSLNVGDLAPDFTLSDQDGNLISLSQFRRQTVVLYFYPRDHTPGCTKEACGFRDVYADYQGNNIIVLGISTDDP
KSHTKFINKYHLPFPLLCDPDGKVATLYHSYGLKKFMGREFMGIYRQTFIINPDGYIHKIYPKVKPESHAITILQDLS
GEG
>39-A03490
MPVQVGDLAPDFTLTSQTGESVSLKDFRGNKAVVLYFYPKDDTPGCTAEACAFRDSYTIFQETGAEVIGVSADSP
DEHQKFVGKYNLPFTLLSDQGNQVRKLYGVPATLWILPGRVTYVIDNQGVVRHIFDSMLDFKGHVDEALKTLRS
MSAV
>39-M00250
MTSECIRVGLAAPDFAATAVIDQEFKTIKLLEYRGKYVVLFFYPLDFTFVCPTEITAFSDRAEEFSAINTQILGVSVDS
EFSHLAWIQSDRQSGGVGDLNYPLVSDIKKEISAAYNVLDPEAGIALRGLFIIDKGGIIQHATINNLAFGRNVDETL
RTLQAIQYVQANPDEVCPAGWQPGDKTMNPDPVKSKEFFAAI
>39-E02230
MVHSTSILTVGDPAPWFSLPDSNGKNIVLRSHAGKPILLVFYAGDQISECEKIACRLQDIMSLCDRFDLQIFSISLN
PPSTRKTFAENHHLNYSLLSDFKGEVSTKYGAYICDGKNSNTIVYNRVAFLLDRNLRILKIYPLHPLEEFTQKFLGEI
QDLVAQEPPRLIKMQAPVLLIPKVLDLR
>39-F00560
MTLRLGDTVPNFTQSSSEGEINFYDWAGDSWVVLFSHPADYTPVCTTELGTVAKLKPEFDKRNVKVIALSVDDA
DSHKGWIGDINETQQTNINYPILADSDRQVSDLYDMIHPNANASLTVRTVFVIDSNKKLRLTLTYPPSTGRNFSEI
LRVIDSLQLTDNYSVATPADWQDGEDCVIVPSLKDPEELKQKFPKGYKEIKPYLRMTPQPNK
>39-J00990
MMLTSNDFSGLLNQRFFRNFLPIPAAGTLHLGKKTPDFELPDITNNGKVRLSEYRGNTPVILAFTRIFTEKQYCPL

CYPHIKALNENYRAFVERGAEILMITSTDTAQSQTVVKDLGLKMPLLSDSSCGIFRLYHVGQALGAPLPAQFVLD
REGILQYRHLFSFLEPNATVPKLLSILDELAN
>39-Q00510
MALTASTMLSLGTIAPDFQLPDVTSGEIISVDTFKDKKALLVMFICCHCPFVKHIENELAKLGHDYQPLGIGIVAIS
ANSIKTHPQDGPEHLKNQAETVGFSFPYCYDETQEVAQAYTAACTPDFFLFNSDRQLVYRGQLDDSRPGNNLP
VTGADLRAALDAILADQPINSDQKPSIGCNIKWHPQ
>39-C03410
MKGTLINSYAPDFELPGVDDEVHHLARYLQKFQVVIVIFMSNSCPYVQSYVGRMKALQTYLQTVEATIVGINAN
DPLQSPSDSFEQMKIFAQDHDLNFPYIRDVTQDVAHCFGAETTPEAFVLDNQGILRYRGGLDDNPESPEAVTIN
YLKNAVSQLLASVAIAPETTPPIGSPIQWRR
>39-M02570
MVIEKVPDVVFKTRVRDESVGGPNPFRWQDRTTQEIFGGKRVVVFSLPGAFTPTCSSTHLPRYEELYDEIKAQGI
DEIVCVSVNDAFVMFQWGKQQGADKVFLLPDGNGEFTRKMGMLVDKSNLGFGMRSWRYSMVVNDGKIEK
IFVEPDFGDNCPTDPFEVSDADTMLAYLKG
>51142-1296
MLRRQILAILLAIAVIFSGTQTPALALGGPQPPLNESAPNFTLPTNTGDGEISLSDYRGQWVVLYFYPQDFTPGCL
EARRFQQDLPKYEAKNTQILGVSVDDVNSHREFCDAEGLKFPLLADTTGEVSKTYGSFLTGMSLRHTYLIDPDGL
REKFLGVRPAIHSQEVLAYLDEVNSNH
>51142-3358
MALAVGTVAPDFTTVDDEGNTVSLSDYKGKVVVLYFYPKDDTPGCTKEAQSFRDNYQQYQDKDMVVFGVSM
DDQASHKAFKEKYGLPFQLLVDKDGKLTKAYDVEGGGYSKRVTYIINGEGKISYVDEKVKTDTHAQDILGVAG
>51142-2409
MTIEGCLRVGQVAPDFTATVVIDQEFQTKKLSSYRGKYVVLFFYPLDFTFVCPTEVIAFSDRYEEFAKINTEILAVSV
DSEFSHLAWIQTPRTEGGVGDIAYPLVSDLKKEISTAYNILDPESGVALRGLFIIDKEGVIQHATINNLSFGRSVTEL
RTLKAIQHVQTNPEEVCPADWQEGDKTMIPDPIKSKIYFSAV
>51142-0135
MALQLGDTVPDFTQETSEGTISFHEWAGDSWVVLFSHPADYTPVCTTELGTVASLKPEFDKRNVKILALSVDSV
DSHKGWINDINETQNTNVNYPIIADPDRKVADLYGMIHPNSLNNLTVRSVFIIDPNKKLRLTLTYPASTGRNFDEI
LRVIDSLQLTDYHQVATPANWKDGDDCVVVPSISTEEAKQKFPKGVTEVKPYLRMTPQPNK
>51142-4124
MMLTSTDFTGLINQRFFNNFLPIPATNQLLLGSIAPDFILPDITNNRMLRLGDYFHQQPVILAFTRIFTEKQYCPFC
YPHIVSLNKYYEQFVERGVDVLMITSTDTQQSQIVVRDLGLKMPLLSNPDCQVFRKYHTGQALGAPLPAQFVLD
KTGTIQYKHLFSFMDHNASVDQLLLKIS
>51142-3126
MGAIETVPSVVFKTRVRDESVPGPNPYRWQDLTSEEIFKGKKVIVFSLPGAFTPTCSSNHLPRYEELYDEFKALGV
DEVICISVNDAFTMFQWGKQQEAKNVFLLPDGNGEFTRKMGMLVDKSNLGFGMRSWRYSMLVNDCKIEKM
FVEPGYEDNCPTDPFEVSDADTMLAYLKGTESSGVSEPVKAFVG
>51142-3631
MLMVKTLSTMLPLGTKAPNFSLEDVVSGKKISLDTFADKKALLVMFICVHCPFVKHLQDALAALGKEYVDKGLGI
VAISANDITTHPDDSPEHMKKMAETLGLNFPFCYDESQEVAKAYTAACTPDFFLFDRDNSLVYRGQFDDSRPG
NDVPITGKDLKAAIEDVLRDRKVDTQQKPSIGCNIKWKAGNEPPYFGA
>7424-2927
MFGRRVLSLFLAFGLTVLTWFNTTYTAAALGGAQPPLNQPAPQFTLPTNTGDGEISLSDYHGKWVVLYFYPQDF
TPGCTLEARRFQEDLAKFEAKNAQIIGVSADDINSHAEFCDSEGLKFPLLADTTGKVSKAYGSWLGSMSLRHTYL

IDPDGNLRDIFLGVRPAIHSAEVLARLDELQQNS
>7424-4280
MSNLPQIGEKVPNFTGKNQEGILVSLADFSSHWLVLYFYPKDNTPGCTTEAIDFSQFLGEFTALGAKILGVSPDSE
KSHGKFIEKHNLSIQLLSDPDHQVAEAYGVWGLKKFMGKEYMGIIRSTFLIDPDGKLVYTWPNVRVKGHAEAVL
KKLEEVM
>7424-3638
MALAVGTEAPNFTTVNDEGKTVSLSDFKGKVVVLYFYPKDDTPGCTKEAQSFRDNYQEYQTKDMVVLGVSMD
DQASHKQFKEKYGLPFNLLVDTDGAITKAYDVEGGGYSKRVTYIIDAEGKIMYVDEKVKTDSHAKDILSTVG
>7424-5046
MSVKVGDRAPDFTLSSQSGEKVSLKDFLGKKAVVVYFYPKDDTPGCTAESCAFRDSYEVFKEMGAEVIGISGDS
PDSHKSFASKYNLPFILLSDTNNQVRKLFGVPATLFVLPGRVTYIIDKEGIIRQIFDSAFDFKAHVNEALKTLQGVK
>7424-0090
MEGCLRVGQNAPDFTATAVVDQEFKTIKLSDYRGKYVVLFFYPLDFTFVCPTEIIAFSDRYEEFKKINTEVLGVSVD
SEFSHLAWIQTDRKSGGIGDIAYPLVSDIKKEISTAYNVLDPEAGVALRGLFIIDKEGVIQHSTINNLSFGRSVDETL
RTLKAIQYVQTHPDEVCPAGWQEGDKTMNPDPVKAKVYFASV
>7424-0397
MALQLGDTVPDFTQESSEGTISFHEWAGDSWVVLFSHPADYTPVCTTELGTVANLKPEFDKRNVKVLALSVDD
VESHKGWINDINETQKTTVNYPILADPDRKVADLYGMLHPNSLTNLTVRSVFIIDPNKKLRLTITYPASTGRNFDEI
LRVIDSLQLTDNYQVATPANWKDGEDCVVVPSIPTEEAKQKFPKGVTEVKPYLRMTPQPNK
>7424-1402
MTANSGLFNRRFAQNFIPLPGFNVPSVGSLAPNFTLPQVGGNIIQLSDYRGQKSVILAFTRIATEKLFCPYCYPHIL
ELKERYEEICDRGSELLMITSTDSIQSQEIVKNLRLPYPFLYDPECKIFRRYGAGQALGVPLPAQFIINLEGRITYRHL
FSFIDGHASTDTILNQLDQL
>7424-4031
MALTQSTMLTLGTTAPDFQLPDVVSGQTISLKNFEDKQALLIMFICQHCPFVKHIKQELANLGQDYVPKGLGIIAI
SANDVANYPSDSPENLKAMAQELEFNFPFCYDETQETAKTYTAACTPDFFLFDSDRKLVYRGQLDDSRPSNNLP
VTGKDLRAAIENILAGKPVNSDQKPSIGCNIKWKPGHEPSY
>7424-2508
MLPNREGQKVPDAKFRLRVNNEWVDKTTDDLFAGKTVVVFSLPGAFTPTCSSTHLPGYNDLAPVFKDNGVDD
ILCISVNDAFVMNEWAKDLNANNVTLIPDGNGEFSEGMGMLVDKSDLGFGKRSWRYSMLVKDKTIEKMFIEP
EEPGDPFKVSDADTMLNYINPSAKKKPPVTMFTKVGCPFCTRAKNMLQEKGIEYEEIPLSKTVTTKTLSAVSGAK
TTPQVFIGGQLIGGSEALEEYLKTHY
>7425-2289
MSFSFRRRFLLLACFLSLSLLLPALPALALGGDLPPLNQPAPPFALPTNSGTGTVTLDDYRGQWVVVYFYPKDFTS
GCTLEAQRFQRDLAQYQTRNTQIIGISADSVQSHAEFCDSEGLKFPLLADTTGRVSQAYGSWLGFMSMRHSFIV
DPQGILRERFVKVNPAIHSTEVLARLDELQQFL
>7425-1511
MTLNPGDSAPDFTLADASGNLVSLAALRGKWVILYFYPRDNTPGCTKEACGFREIYGELQQKNVVLLGVSGDD
ARAHQKFAQKYDLPFPLLCDPEALVAKAYESYGPKKFMGKELQGIYRQTFILDPEGKIARIYRKVKPDPHPAEVLA
ELDELLASEAQA
>7425-3716
MAVKVGDRAPDFTLPSSSGANVSLADFIGKKPVVLYFYPKDETPGCTAQACTFRDEYAVFQDLGAEVLGVSSDS
VEAHKKFIANHRLPFQLLSDTGAQLRKLYGVPATLGILPGRVTYIIDQAGIVRHIFNSQMNFKGHIEEAIAALKQLQ
TA

>7425-5136
MALAVGTMAPTFTVKDTNGNTVSLADFAGKTVILYFYPKDDTPGCTKEACSFRDNYTAYQGKDLVVLGVSMDD
EASHQAFTQKFNLPFPLLADVDGAITGAYQVAGEKNGFRYAQRVTYVVDGTGKISHVYDSVKTDTHATDILADL
GY
>7425-0928
MTHYECLRVGQPAPDFTATAVFDQEFKTIKLSDYRGKYVVLFFYPLDFTFVCPTEITAFSDRYSEFSSINTEILGVSV
DSEFSHLAWTQTDRKSGGVGDLNYPLVSDIKKEISAAYNVLTEGGISLRGLFIIDKDGVIQHATINNLAFGRSVDET
LRTLQAIQYVQSHPDEVCPAGWKPGEKTMNPDPVKSKVYFSAV
>7425-0215
MPLRLGDQVPNFTQASSTGEINFYDWAGDSWVVLFSHPKDYTPVCTTELGVVARLKPEFDKRNVKALALSVDD
VESHLGWIKDIEETQNVTLNYPVLADGDKKVSELYDMIHPNSSTGNTLTVRSVFIIDSQKKLRLTFTYPASTGRNF
DELLRVIDSLQLTDNYSVATPANWADGGDCVIVPSVSDEEAKQKFPKGFNAVKPYLRLTPQPNK
>7425-4300
MGLRIPTLSSSNFTGLVNDRFWKNFLPIPATNQMEIGSATPDFELPDVTHNQNLRLSNLYRQQPVVLAFTRIFTE
HQYCPLCLPHIKALNEAYPQFQQQGVEVLLITSTDQLQSQKVVTDLQLNLPLLSNPSCDVFRRYGTGQALGAPLP
AQFVIDTQGKLRFKHLFSFFDNNASITTLLQALAS
>7425-0955
MARTESTMLPLGTIAPDFQLPDVVTGKTISLDSFIGQKALLVMFICRHCPYVKHVQVELARLGQDYLDQGVGIV
AISANDADKYPNDAPESLKQMAEELDFNFPLCFDQTQSTAKVYTAACTPDFFLFDADRKLAYRGQLDDSRPSSD
RPVTGKDLRAALDTVVAGGVPDPDQKPSLGCNIKWKPGNEPAYYG
>7425-1596
MAVIERVPNVTFKTRVRDESVPGPNPYRWQDLTTEDIFKGKKVVVFSLPGAFTPTCSSNHLPRYEELYEEFQAQ
GVDQIICISVNDAFVMFQWGKHIGAKNVFLLPDGNGEFTRKMGMLVDKSNLGFGMRSWRYSMLVDDCKIEK
IFIESEFGDNCPIDPFEVSDADTMLAYLKGSESAGVSTPRLAFVG
>8801-1115
MVSIMSRRSLFKWLLALCLTLFSSSFLITPALALGGTQPPLNEPAPEFTLPTNSGEGEISLSDYRGQWVVLYFYPKD
FTPGCTLEAKRFQQDLPKYQERNAQILGVSVDDVDSHAEFCDSEGLKFPLLADTTGKVSKTYGSFLTGMSLRHTY
LIDPNGILRERFLGVIPAIHSAEVLEKLDELTISLLEVRRFIT
>8801-3492
MSNFPKIGEPAPNFAAKNQKGETVSLSDFNAQWLVLYFYPKDNTPGCTTEAIDFTHHQAEFDALGATIIGVSPD
SEKSHCKFIEKHHLSLQLISDEDHQVAEAYGVWGLKKFMGKEYMGIIRSTFLIDPDGKVAETWTKVKVKNHVEG
VLNKLQELKGNSQQTLGYP
>8801-1631
MALAVGTVAPNFTTVDDEGKTVSLSDFKGKVVVLYFYPKDDTPGCTKEAQSFRDNYEQYKGKDMVVLGVSMD
DQTSHKQFKEKYGLPFQLLVDTDGTLTKAYDVDGGGYSKRVTYIIDEEGKISYVDDKVQTATHAQDILSIVG
>8801-1491
MTTQGCLRVGQLAPDFTATAVIDQEFQTKKLSGYRGKYVVLFFYPLDFTFVCPTEITAFSDRYEEFTKINTEVLGVS
VDSEFSHLAWIQTDRKEGGVGDVAYPLVSDIKREISIAYNVLDPDAGVALRGLFIIDKEGYIQHSTINNLSFGRSVD
ETLRTLKAIQYVQSHPDEVCPAGWEEGDRTMIPDPVKAKVYFSAI
>8801-4218
MALQLGDVVPDFTQESSQGTISFHEWAGDSWVVLFSHPADYTPVCTTELGAVASLKSEFDKRNVKVLALSVDS
VESHRGWINDINETQNTTVNYPILADADKKVADLYGMIHPNSLNNLTVRSVFIIDPNKKLRLTITYPASTGRNFDE
ILRVIDSLQLTDYHQVATPVNWKDGDDCVVVPSISTEEAKQKFPKGVTEIKPYLRMTPQPNK
>8801-3616

MMLTSTDFTGLINRRFFQNFLPIPASNSLALGSVAPDFSLPDITNNRTVKLSDYRNQQPIVLAFTRIFTEKQYCPFC
YPHIINLNQNYERFIEQGIEVLMITSTDAKQSQIVVRDLGLKMPLLSNPDCRVFRRYHTGQALGAPLPAQFVLDK
TGKIQYKHLFSFFDHNAKFDKLLKFL
>8801-4240
MVLTPSTMLPLGTKAPNFQLPDVVSEKTISLETFANKKAILVMFICQHCPFVKHIQTQLAKLGHDYVDKGLGIVAI
SANDVANYPDDSPSNLKKMAQELGFNFPVCYDESQEIAKAYTAACTPDFFLFDGDYQLVYRGQLDDSRPGNNI
PVTGKDLRIAIDAILGGNSITIEQKPSIGCNIKWKAGNEPPYYG
>7421-3389
MRKLLLKSSLLFALLLAGPQAALLAAPRVGEAAPAFELPVAAGKKISLKDFQGKWLVLYFYPKDMTSGCTIEAQRF
QKDLAQYKQLGAEIVGVSADALDSHSMFSKKEGLTFLLASDVGGKVAQAYDSWYGLGDMGMAARNTYLIDPQ
GRVAKVFSNVDPTGHSREVLSTLKQIQAKAQR
>7421-2376
MSEPLNVGDPAPEFAAEQTSGERLSLADLRGKKVVLYFYPRDNTPGCTKEACALRDRYARLQAAGVTVVGVST
DGVRSHQKFTEKFALPFALLADTEGAIAQAYGAWGEKKFMGRTSIGILRHTVVIDEAGRIAHIYRKVKPEEHADQ
ILRDLGIAFEA
>7421-2375
MALNVGDKAPDFTAKDTHGNTVSLASLAGKKFVLYFYPKDDTPGCTKEACSFRDNWSTYQAQGLGVYGVSM
DDEASHQAFTSKYALPFPLLADTDGTLAKAYDADAGNYAKRITYVIDEAGKIAQVFTSVNSASHASDVLAAVGV
>7421-0506
MAIEVGRPAPDFTLDGSQGPVTLSQYRGKNNVLLAFYPGDFTPVCTKELTCFVEDWSKFQGKDTVILGISSGSV
DSKSKFAQSLGAQFPLLSDGEKRVAGLYDVSGFLGVARAYFIVDKQGVLRYKHVEALPFFKREDEELLGVLAEL
>7421-3158
MTASVGLPAPSFTLKSTKGATSPKNLGEPIALTDYRGKWLIFFFYPLDFTFVCPTEILALSDRYEEFQDLDTEVLGCS
CDSVFSHWAWISTPREKNGIAGLKFPLVADFTKDVAEAYGVLNEKEGYSQRGLFIIDPDGILKYATITDDNVGRSV
EETIRVLQALQNGGLCPGDWKPGQKVLEVV
>7421-2155
MALRLGDKVPDFTQESTEGPIQFYDWAGDSWVVLFSHPKDFTPVCTTELGEVAKLKPEFAKRNVKVIALSVDD
VDSHKGWAQDIEETQGAALNFPVLADADRKVSDLYDMIHPSANDTLTVRSVFVIDPNQKLRLTLTYPASTGRNF
DELLRVIDSLQLTDNYSVATPANWQDGEDCVIVPSLKDEAVLKEKFPKGYTVVKPYLRMTPQPNK
>7421-0029
MTADTRLFNQRFAKNFLPLPGFSVPAVGEAAPDFVLPRVGGAPVRLSDYRGRMPVVIAFTRIFTEKLFCPFCYPH
LQDLKNRYGEIRQAGAELLMVTSTDPVQSAQVVADLALPYPFLFDPGCTLFQAYGAGQALGAPLPAQYIVDMT
GTIVYRHLFSFIDSNASTDEVLAVLKALQKV
>7421-3964
MKRKAFVMAALSLALVAANVSTVQADALVGQPAPSFTAADTNGKSHALTDFKGKYVVLEWTNYDCPFVVKQY
STQAMQEMQKMAAGKGVVWLSVNSSAAGKQGNYPAEKWNTLVKEKGAAPKAILLDGDGKIGQLYGAKTTP
HMYIINPEGKLIYNGAIDDKPTTDAATKPQTNYVAAALTEAMAGKPVTVATTRPYGCSVKY
>7421-2108
MARTASTMMELDTLAPAFVLPDVSTGQMLTLASFAGQPALLVMFICRHCPFVKHIQGELARLGKDYADSGLAIA
AISANDAVKYPVDSPESLKEMVDELGFTFPVLYDESQQTAIAYQAACTPDFFLFDAARRLVYRGQLDDSRPGNDL
PVTGKDLRIAIDAVLGGKAPAIEQKPSIGCNIKWKEGNTPPYYG
>843-15330
MSRRQLLSFLIAVILAFFAFIPDANALGGPQPPLNQLAPDFTLPTNTGEGNISLSDYRGKWVVLYFYPKDFTPGCT
LEARRFQQDLPKYMAKNTQVLGVSADDVDSHAEFCDSEGLKFPLLADTTGDVSKAYGSWMGYVSLRHTYLIDP

QGILKEIYLGVNPAIHSAEVLARLEELQASS
>843-44920
MTLEVGQKAPEFATPNQRGEISKLADFAGQWLVLYFYPKDNTPGCSAEAIDFTALSPQFQQLNAVILGVSPDSE
KSHCRFIEKHNLTIQLLSDPEHQLAEIYQVWGLKKFMGKEYMGIRRSTFLIDPRGNIAYIWSNVKVKAHAEAVLK
KLEELQ
>843-60930
MALTVGTIAPNFTTTDDTGKTVSLSDFQGKVVVLYFYPKDDTPGCTKQAQSFRDNYEQYQDKEIVVFGVSRDD
QGSHAQFKEKYGLPFQLLVDTDGAITKAYDVDGGGYSKRVTYIIDGEGKISHVDDKVNTASHASDILCVVG
>843-35830
MTAEGCLRVGQPAPDFTATAVFDQEFKTIKLSDYRGKYVVLFFYPLDFTFVCPTEITAFSDRVSEFSSINTEILGVSV
DSEFAHLAWIQTERKSGGVGDVAYPLVSDLKKEISTAYNVLDPDAGVSLRGLFIIDKEGVIQHATINNLSFGRSVD
ETLRTLKAIQYVQSHPDEVCPAGWQEGDATMVPDPVKSKVYFAAV
>843-36510
MALQLGDIVPDFTQDSSEGPISFHQWVGDSWVVLFSHPADYTPVCTTELGTVASLKSEFERRNVKVIALSVDSA
ESHRGWINDINETQNTTVNYPIIADGDRKVSDLYGMIHPNSLNNLTVRSVFIIDPNKKLRLTITYPASTGRNFNEIL
RVIDSLQLTDNYQVATPANWTDGGDCVVVPSIPTEEARSKFPKGVEEIKPYLRMTPQPNK
>843-62780
MLPNREGQRVPDVTFPVRQNNQWVNITSEELFKGKTVVLFALPGAFTPTCSTSHLPGYNELAPVFRENGVDSI
VCLSVNDTFVMNEWAKDQECDNVVLIPDGNGEFSAGMGMLVDKADLGFGQRSWRYSMLVKDGVIEKMFIE
PEEPGDPFKVSDAETMLNYIGRDATSRSLRAMSGNTTTPQVFINGQLIGGSEELAAYLGA
>843-59730
MARTESTMLDLGTKAPSFALPDVVSGETISLDSFAAKTALLVIFLCEHCPFVKHIQEELTRLGRDYANTNLGILAISS
NDVEKYPDDSPENLKKMAITLDFKFNLCYDESQEVAKAYTAACTPDFFLFDSQRILVYRGQLDDSRPSNGIPVTG
KDLRAAIDKVLTGQPVPTDQKPSLGCNIKWKLGNEPPYYG
>29133-F2425
MISRRTFLNILFASCISVISWLNFIPAADALGGKLPAINQPAPEFTLPTNTGDGKISLSDLRGKWLVLYFYPKDFTSG
CTIEARRFQQDLPQYLAKNTQIIGVSADDIDSHAKFCDSEGLKFPLLADTDGSVSKAYGSWLGFLSMRHSFIIDP
QGVLRETFVKVNPTVHSSEVLARLEKLSTAS
>29133-R6477
MTNIPQIGQPAPDFSTPDQNGNSVTLDDFSSQWVILYFYPKDDTPGCTTEAKDFTELYQDFSALGAKILGVSPDL
GKSHCKFISKHNLSITLLSDPEHILTEAYGAWRLKKFMGKEYMGVARSTFLISPDKIIAYTWPNVKAKGHAQAVLT
KLQELAAT
>29133-F2872
MPIKVGDTAPNFTLPAQNGSTVSLQDFRGTKAVVLYFYPKDDTPGCTAESCAFRDQYEVFKSVGAEVVGVSADS
SESHQKFAAKYNLPFTLLTDKGDQVRKLYGATAAFGLFPGRVTYVIDQQGVVQYVFDSMLNFKGHVEEALKTLQ
QIAK
>29133-R0493
MPLAVGTDAPAFTAKDTNGNTVSLSDFAGKTVVLYFYPKDDTPGCTKQACSFRDAQSEYQGKDIVVLGVSADD
EVSHQAFTQKYNLNFPLLADYDKSLITAFDVDGGGYAKRVTYVIDPNGKITHVDNAVNTTTHASDILAALGL
>29133-F6082
MSLTYGTEGSLRVGQQAPDFTATAVVDQEFKTIKLSDYRGKYVVLFFYPLDFTFVCPTEITAFSDRYEEFKKINTEV
LGASVDSEFSHLAWIQTDRKSGGVGDLNYPLVSDIKKEISAAYNVLDPAAGIALRGLFLIDKDGIIQHATINNLAFG
RSVDETLRTLQAIQYVQSHPDEVCPAGWQPGDKTMNPDPVKSKVYFSAV
>29133-F6498

MALRLGDTVPNFTQASTHGDIDFYQWAGDSWVVLFSHPADFTPVCTTELGTVAKLKPEFDKRNVKAIALSVDD
VESHKGWVGDIEETQSTTLNYPILADADRKVADLYDMIHPNANAAVTVRSVFVIDPNKKLRLSFTYPPSTGRNF
DELLRVIDSLQLTDNYSVATPADWKDGEDVVIVPSLKDPEVLKEKFPKGYEEIKPYLRMTPQPNK
>29133-F6083
MLTSTDFSGLLNERFFRNFLPVPARNQLRLGVGTPDFQLPDITNGTLVKLSDYKGKQPVLLAFTRIFTEKQYCPFC
FPHIKALNENYEEFKNRGIEVLMITSTDEGQSQIVVKDLGLKMPLLSDPSCRVFRTYQVGQALGAPLPAQFVLDK
EGKLSYWHLFSFLDRNASIEILLEQFN
>29133-F6135
MNILETIDTPIGSYAPDFELPGIDGQVHHLRRYLEKFRAVGVISMCNHCPYVEWYIDRLKKIQAKFAPKGFTLIG
MNGSDGNHETRTSFENMKAFAERHNLNFPYLWDSTQDVTQSFGATKTPMAFLIDANGIVRYKGKIDNHPQD
ASAVGEEYLRTAIASLFLGQPIDVPQTEPVGTTLIWRN
>29133-F2528
MALTASTMLPLGTKAPDFNLPEVVSGKATSLSTFADKKALLVMFICRHCPFVKHIQQELVQLGKDYFTSDLAIVAI
SANDAKNYPDDAPESLQAFATEQGFNFTLCYDESQETAKAYTAACTPDFFVFDEQRKLVYRGQLDDSRPSNGKP
VTGADLRAAIEAVLAGKPVTSDQKPSVGCNIKWKPGNQPSYFG
>29133-R4657
MVANRRGQRVPNVTFHTRKDNQWVDVTTDQLFANKTVVVFSLPGAYTPTCSSTHLPGYNELAGIFKENGVD
DIICISVNDAFVMNEWSKDQEAENITLIPDGNGEFTEGMGMLVDKSDLGFGKRSWRYSMLVRDGVINQMFIE
PDEPGDPFKVSDAETMLRYINPQAVKPEVVSLFAKVGCPFCARAKAMLKEHGINYEEITLGKDITTRSLRAVTGA
TTVPQVFIDGKLIGGSEALEAYFAAK
>29133-R6111
MNHNLLYRRQLLFYLGLGVVGIGAATTFSNFRKVNAPSISPAKSNNTQDLETTVKTPAGKSLPEFQGISQWLNSS
PLAIADLKGSVILIQFWTFACINCQRTLPYITKWHQQYESQGLKVIGIHTPEFAFERDPNNIKKALQQHKITYPVP
VDNEYKTWNAYENQYWPHIFLADRQGLLQYDHIGEGAYEKIEQTIRQLLG
>med4-0903
MKSLFGLFVILISLIFNLKSAIAFDYAPEIGDSAPSFHLEGINKSIKSKKIWDSNELIGKWIVLYFYPKDFTAGCTLEAK
GFSQLKNDFSKYNAEIIGISADNQDSHDSFCSEKSINYTLLSDPNGTISAKYGSWIPPYSDRNTFLISPEGKITYRWI
SVLPINHAKEVLNILKKNI
>med4-0079
MALKVGDKAPEFNLKDSFEKDVSLKDFKGKRIILYFYPKDNTPGCTKEACNFKENWDLLKQNNFVVLGISKDSA
ASHQKFIEKFDLPFVLLTDPEPYKTSSDYDSYGLKKFMGKEYMGMMRNTFLIDVDGKVEKIYLKVKAAIMADHII
SDLGLR
>med4-0345
MQIGDKVPQFSLLDQNGTKRSNNGLKTPLVLFFYPKDDTPGCTIEVCGFRDKYDLFKVLGAQVWGVSNGSSSS
HLAFANKNKLQYPLLCDKNDSLRKAFKVPKVLGLLDGRVTYVIDRNGFVKHIFRDLLNGPEHIKEAIRVLKEIQNQ
>med4-0856
MSLRVGQEAPDFTATAVYDQEFKDITLSSYKGKWVVLFFYPLDFTFVCPTEITAFSDEFNKFSDLNTEVLGVSVDS
KHCHLAWIQTPRNEGGIGDINYPLVSDLKREICQAYNVLNDDGEADRGLFLINPEGIVMHTTVNKAPVGRNVD
ETLRILQGYQYVAANPDEVCPANWTPGEKTMLEDPKGSKEYFSAL
>med4-0283
MVKTNSMVLELGFQLPNFKMLNANSSNNQYFNSHNLDNRHLLLMFICAHCPFVKYIENQIFTLSKEIENTVQT
VAISSNDIVTHPSDSPDYLRLQAQSQGWSFPYLYDENQNFAKELKAACTPDFYLFSNEGDGDFLLFYHGQLDDS
RPGNNIPLSGKDLRSAVKDLNQDNSYPSNQIPSLGCNIKWTPGKEPSWFK
>9312-0897

MRTLVVSILISFFLLINCNSTLAFDFAPEVGDSAPNFQLEGFNKNIKSKKIWELSDFQGKWLVIYFYPKDFTAGCTL
EAKGFSELKKDFSKYNAEIIGISADKKDSHESFCSEKSITYTLLSDPEGIISDKYGSWIPPYSDRNTFLISPEGTISYRW
ISVLPINHAKEVLNVLKKKI
>9312-0082
MALKVGDKAPEFKLKDSFDKEVSLNDFKGKRIILYFYPKDNTPGCTKEACNFKENWDLLQKNNIVVLGISKDNA
VSHQKFIEKFNLPFILLTDPEPFKVSSDYDSYGLKKFMGKEYMGMMRNTFLIDTDGKIEKIYLKVKAAIMADHIIA
DLRLQ
>9312-0353
MQIGDKIPEFSLLDQNGIKRSNKGLKNPLVLFFYPKDDTPGCTIEVCGFRDKYDLFKVLGAQVWGVSNGSSSSH
LAFANKNKLQYPLLCDTNNSLRKTFKVPNVLGFMDGRVTYVIDRNGIVRHIFRDLLNGPEHIKEAIRVLKEIQNK
>9312-0944
MSLRVGQEAPDFTATAVYDQEFKEITLSGLRGKWVVLFFYPLDFTFVCPTEITAFSDRYQDFSSIDAEILGVSVDSK
HCHLAWIQTPRNEGGIGDINYPLVSDLKREICQAYNVLNDDGEDRGLFLINPEGIVMHTTVNKAPVGRNVDETL
RILQGYQYVAANPDEVCPANWTPGEKTMLEDPKGSKEYFSAL
>9313-1012
MKRSELLRVITGAAVLSISQCLGRPSQALAMEGKLPALDAQAPDFELEAAAQEGALAEKLQRDAFLGSWLVLYFY
PRDFTSGCTLEARGFQRDLSDFKAAGAAVVGVSADGTEEHVSFCSSEGLGYTLLSDPGGVVSERYGSWSSPFSQ
RHTFLIDPDGILRARWKDVSPSRHSQDVLRALKELQQS
>9313-1622
MTLKIGDPAPDFNLPNQDGTLVQLASLRGQRVVIYFYPKDDTPGCTKEACNFRDQWERFEANDIKVLGISKDG
ATSHTKFIKKYQIPFTLLSDAEPCPVASSYESYGLKKFMGREYMGMMRHSFVVDPKGNLELIYLKVKAATMADQ
ILKDLGLD
>9313-0387
MALQVGDKAPAIDLVDQNGNKRRSNDLKGKVLVLFFYPKDDTPGCTAEACSFRDNYSVFENLGSEVWGVSGD
DDISHRQFAERHGLPYALLSDKDNTLRRAFEVPRTLGMLPSRVTYVIDGQGTIRHIFNNLLDGPAHMREACRVV
EEIKKKR
>9313-0754
MTTNECLRVGQQAPDFNATAVVDQEFKEISLSQYRGKYVVLFFYPLDFTFVCPTEITAFSDRYTDFSSRNTEVLG
VSVDSQFSHLAWIQTSRKEGGLGDINYPLIADLKKEISTAYNVLDDEAGVALRGLFIIDPDGVIMHSTINNLPVGR
NVDETLRVLQAFQYVQSHPDEVCPANWTPGEKTMKPDPVGSKEFFASIN
>9313-1186
MPFPQNVPDVTFKTRVRDESVPGSNPFRWQDLTSAEIFKGRKVVVLALPGAFTPTCSSNHLPRYEELHAELVAQ
GVDQIVCLSVNDAFVMFQWSKHLGTKNIFMLPDGNGEFTRKMGMLVEKSNLGFGLRSWRYSMLVNDCNIEK
MFVEPGFSDNADGDPFEVSDADTMLAYMKGVQSAGVSSPCREFQG
>120-0932
MVINYIKNIIFILIFAITITSYPSIVFSNSVPNINEQAPGFELEGYNPERPDKTKWSSNDFKGSWLILYFYPEDATSGC
TIEAKGFQRLNKEFLSHKANIVGISKDSKASHKLFCSDQKLDFTLLSDKDSQISKSFGSWQEGFSSRNTFLIDPNGI
IKYRWLAVIPIKHAKEVLSVLNRIDQT
>120-0093
MSLQIGEKAPDFTLPDQDGKLIKLSSFKGERVVIYFYPKDDTPGCTKEACNFRDRWDILQNHKINVIGISKDPAN
KHSKFINKYQLPFILLTDLEPCPVATSYESYGLKKFMGKEYMGMKQTFVIDSEGKLEVIYLKVKAASMADQIVQDL
ALE
>120-0978
MTEGCLRVGQMAPNFTAEAVVDQEFKQISLTDYRGKWVVLFFYPLDFTFVCPTEITAFSDRFSDFSSKSTEILGVS

VDSKHCHLAWIQTPRNRGGIGDITYPLVSDLKREIATAYNVLNDDGEADRGLFLINPEGIIMHCTINKAPVGRNV
DETLRILQGYQYVASNPDKVCPANWNPGDKTMLEDPKGSKEYFASL
>9601-09581
MRNLVISIFISFIFLFNCTSVIAFDFAPEVGDIAPNFQLEGFNKNIKSKNIWELNDFQGKWLVMYFYPKDFTAGCT
LEAKGFSELKKDFSKYNAEIVGISADNQDSHESFCSEKSINYTLLSDPDGIISKKYGSWIPPFSDRNTFLISPKGEISY
RWISVLPINHAKEVLNVLKKKI
>9601-00921
MALKVGDKAPEFKLKDSFEKEVSLSDFKGKRIILYFYPKDNTPGCTKEACNFKENWDLLQKNNIVVLGISKDNAS
SHQRFIEKFNLPFILLTDPEPFKVSSDYDSYGLKKFMGKEYMGMMRNTFLIDTEGNIEKIYLKVKAAIMADHIIAD
LGLS
>9601-03811
MQIGDKIPEFSLLDQNGVKRSNKGLKNPLVLFFYPKDDTPGCTIEVCGFRDKYDLFKVLGAEVWGVSNGSTSSH
LAFANKNKLQYPLLCDTNDALRQTFKVPKVLGFMDGRVTYVIDRKGTVRHIFRDLLNGPEHIKEAIRVLKEIQNQ
>9601-10131
MSLRVGQEAPDFSATAVYDQEFKEVTLSGLRGKWVVLFFYPLDFTFVCPTEITAFSDRYQDFSALNTEILGVSVDS
KHCHLAWIQTPRNEGGIGDINYPLVSDLKREICQAYNVLNDDGEADRGLFLINPEGVVMHTTVNKAPVGRNV
DETLRILQGYQYVAANPDEVCPANWTPGEKTMLEDPKGSKEYFSAL
>9211-08571
MLNLIRILSKNLATILVVFLIASPTYAINSNNINIGDKAPYFELDGYSKNNPDKDLWKLSDFKGVWTVLYFYPKDFT
SGCTIEARSFEKLSPEFEKLNAMIVGISNDIQESHESFCSKESLSFTLLTDLQGIVSEKYSSWNKPYSKRNTFLIDPE
GVIRYSWMNVNPLKHPTDVLKKLTELQV
>9211-15101
MSLNIGDKLPDFSLFDQEGKIRTSKQCKGKKLVLFFYPKDDTPGCTAEACGFRDKYDLFKLFGAVVWGVSNDNQ
LSHQKFAEKNKLPFPLLCDENNSLRTLFGVPKVLGLLDGRVTYLVDSTGVIRHIFNDLLNSTKHVTTALEILEEIK
>9211-00881
MIAMPLQVGDTAPEFTLPDPNGTLVSLSSFRGQRVVIYFYPKDNTPGCTKEACNFRDLWKDFKSNNIKVIGISKD
SGTSHLKFISKYQLPFTLLSDSEPCPVASSYESYGLKKFMGKEYLGMMRKTFVIDSEGKIELIYHKVKAEIMAKQIL
DDLSLT
>9211-09041
MMTNECIRVGQKAPDFTATAVIDQEFKEISLSNYRGKYVVLFFYPLDFTFVCPTEITAFSDRYSEFSSKNTEVLGVS
VDSQFSHLAWIQTQRNDGGIGDINYPLVADLKKEISLAYNVLDDAEGVALRGLYIIDPDGVIMHATINNLPVGRN
VDETLRVLQAFQYVQANPDEVCPANWTPGEKTMKPDPEGSKEYFSSL
>9215-09891
MRNLVLGIIIPIILLFNYNSALSFDFAPEVGDQAPNFHLEGFNKNIKSKSIWELSDFQGKWLVMYFYPKDFTAGCT
LEAKGFSELKKDFKKYNAEIVGISADNQDSHESFCSEKSINYTLLSDPEGVISDKYGSWIPPFSDRNTFLISPEGNIS
YRWISVLPINHAKEVLNVLKKKI
>9215-00921
MALKVGDKAPEFKLKDSFEKEVSLSDFKGKRIILYFYPKDNTPGCTKEACNFKENWDLLQKNNIVVLGISKDNAS
SHQKFIEKFNLPFILLTDPEPFQVSSDYDSYGLKKFMGKEYMGMMRNTFLIDTEGKIEKIYLKVKAAIMADHIIAD
LGLS
>9215-03801
MQIGDKIPEFSLLDQNGVKRSNKGLKNPLVLFFYPKDDTPGCTIEVCGFRDKYDLFKVLGAQVWGVSNGSTSSH
LAFANKNKLQYPLLCDTNDSLRKTFKVPKVLGFMDGRVTYVIDRKGTVRHIFRDLLNGPEHIKEAIRVLKEIQNQ
>9215-10441

MSLRVGQEAPDFSATAVYDQEFKEITLSGLRGKWVVLFFYPLDFTFVCPTEITAFSDRYNDFSALNTEILGVSVDS
KHCHLAWIQTPRNEGGIGDINYPLVSDLKREICQAYNVLNDDGEADRGLFLINPQGVVMHMTVNKAPVGRNV
DETLRILQGYQYVAANPDEVCPANWTPGEKTMLEDPKGSKEYFSAL
>9215-03081
MVRTNSMILELGFQLPNFEMLNANSSNNEYFNSHNLDNRHLLLMFICAHCPFVKYIEKQICTLSKEIENIVQTVA
ISSNDIVTHPSDSPENLRLQAESQGWNFPYLYDENQNFAKELKAACTPDFYLFSNKGDGNFLLYYHGQLDDSRP
GNNIPLSGEDLRSAVRDLNQDNTYPANQMPSLGCNIKWTPGKEPSWFK
>9301-09561
MRSLVVSILIPFIILFNCYSALSFDFAPEVGDYAPNFKLEGFNKNIKTKKTWELSDFEGQWLVMYFYPKDFTAGCT
LEAKGFSELKKDFSKYNAEIVGISADNEDSHESFCSERSINYTLLSDPDGIISDKYGSWIPPFSDRNTFLISPDGKISY
RWISVLPLNHAKEVLNVLKKKI
>9301-00911
MALQVGDKAPEFKLKDSFEKEVSLSDFKGKKIILYFYPKDNTPGCTKEACNFKENWDLLQKNNIVVLGISKDNAS
SHQKFIEKFNLPFILLTDPEPFKVSSDYDSYGLKKFMGKEYMGMMRNTFLIDTDGNIEKIYLKVKAAIMADHIIAD
LGLS
>9301-03801
MQIGDKIPEFSLLDQNGVKRSNRGLKNPLVLFFYPKDDTPGCTIEVCGFRDKYDLFKVLGAQVWGVSNGSTSSH
LAFANKNKLQYPLLCDTNDSLRKTFKVPKVLGFMDGRVTYVIDRKGTVRHIFRDLLNGPEHIKEAIRVLKEIQNQ
>9301-10121
MSLRVGQEAPDFSATAVYDQEFKEITLSGLRGKWVVLFFYPLDFTFVCPTEITAFSDRYQDFSALNTEILGVSVDS
KHCHLAWIQTPRNEGGIGDINYPLVSDLKREICQAYNVLNDDGEADRGLFLINPEGIVMHTTVNKAPVGRNVD
ETLRILQGYQYVAANPDEVCPANWTPGEKTMLEDPKGSKEYFSAL
>9303-10801
MLSISQCLGRPSQALAMEGKLPALDAQAPDFELEAAAQEGALAEKLQRDAFLGSWLVLYFYPRDFTSGCTLEAR
GFQRDLSDFKAAGAAVVGVSADGTEEHVSFCSSEGLGYTLLSDPGGVSERYGSWSSPFSQRHTFLIDPKGILRM
RWLAVSPSRHSQDVLRELKELQQS
>P9303-02611
MTLQIGDPAPDFNLPNQDGTLVQLASLRGQRVVIYFYPKDDTPGCTKEACNFRDQWERFEANDIKVLGISKDG
ATSHTKFINKYQIPFTLLSDTEPCPVASSYESYGLKKFMGREYMGMMRHSFVVDPKGNLELIYLKVKAATMADQ
ILKDLGLD
>9303-19011
MALQVGDKAPAIDLVDQNGKKRRSNDLKGKVLVLFFYPKDDTPGCTAEACSFRDNYSVFEKLGSEVWGVSGD
DDISHRQFAERHSLPYALLSDKDNTLRRAFEVPRTLGMLPSRVTYVIDGQGTIRHIFNNLLDGPAHMREACRVV
EEIKKKR
>9303-14621
MTTNECLRVGQQAPDFSATAVVDQEFKEISLSQYRGKYVVLFFYPLDFTFVCPTEITAFSDRYTDFSSRNTEVLGV
SVDSQFSHLAWIQTSRKEGGLGDINYPLIADLKKEISTAYNVLDDAAGVALRGLFIIDPDGVIMHSTINNLPVGRN
VDETLRVLQAFQYVQSHPDEVCPANWTPGEKTMKPDPVGSKEFFASIN
>9303-08311
MPSLQSVPDVTFKTRVRDESVPGPNPFRWQDLTSAEIFKGRKVVVLALPGAFTPTCSSNHLPRYEELHTELVAQ
GVDQIICLSVNDAFVMFQWRKHLGTKNIFMLPDGNGEFTRKMGMLVEKSNLGFGLRSWRYSMLVNDCNIEK
MFVEPGFSDNADVDPFEVSDADTMLAYMKGVQPAGVSSPRRKFEG
>9303-25151
MQSHPEPALMGCFPNMALTPSTMLPLGTPLPSFELKVVPGTNLEIDDGLGALNTINTSTLPTKPLLLMALCAHC

PFVKHIEKELTHLDQDYGDSVQLLAIASNSLITHPQDGPEQLAAQAKQQGWRFPYLLDTDQSLAKALQAACTP
DFFLFAPSPQGLQRLCYRGQLDNSRPGNHAPVTGSDLRAALDAVLNGQDVSPNQQASIGCNIKWHPGQEPP
WFG
>9515-09851
MKSLFSIFIIIISFFFNFKSVIAFDYAPEVGDIAPSFHLEGINKNIKSKKIWDSNELKGKWVVLYFYPKDFSAGCTLEA
KGFSKLKKDFSKYNAEIIGISADKEETHESFCSEQSINYTLLSDPNGTISEKYGSWIPPYSDRNTFLISPKGEIVFRWI
SVLPINHAKEVLNVLKKNI
>9515-00891
MALRVGDVAPEFKLKDSFEKDVSLKDFKGKRIILYFYPKDNTPGCTKEACNFKENWDLLKQNNFVVIGISKDSSA
SHLKFIEKFDLPFVLLTDPEPFKVSSDYDSYGLKKFMGKEYMGMMRNTFLIDADGKVEKIYLKVKAAIMADHIIS
DLGLR
>9515-03821
MKIGDKVPLFSLLDQNGTRRTNEGLKSPLVLFFYPKDDTPGCTIEVCGFRDKYDLFKVLGAQVWGVSNGTSSSH
LAFANKNKLQYPLLCDIDDSLRKTFKVPKVLGLLDGRVTYVIDRNGFVKHIFRDLLNGPEHIKEAIRVLKEIQNQ
>9515-09331
MSLRVGQEAPNFTATAVYDQEFKEITLSSYKGKWVVLFFYPLDFTFVCPTEITAFSDEYEKFSALNTEILGVSVDSK
HCHLAWIQTPRNEGGIGDINYPLVSDLKREICQEYNVLNDDGEADRGLFLINPQGIVMHTTVNKAPVGRNVDE
TLRILQGYQYVAANPDEVCPANWTPGEKTMLEDPKGSKEYFSAL
>1A-09761
MKRSEFIKSFILFSSILFIKPSRLYASINSVKIGKKAPNFLLNGYNKNSPNKKQWSLDDFSKKWLVLYFYPKDFSSGC
TLEAKNFQDNLSNYNKINASIVGISADSEEEHESFCTSENLGYTLLSDANSEISKSYDSWLDPYSKRNTFLINPEGI
VVYKWIGVRPIGHAQEVLKELTKQQKIYA
>1A-15651
MKLKIGDQIPSFSLKDQKGNIRTSNNLKKSLVLFFYPKDDTPGCTIEVCGFRDKYDLFKILGAEVWGISNGSSQSH
LGFANKNKLQYPLLCDQNNILRNQFGVPKKLGFIEGRVTYIINSEGVIKHIFEDLLNGPAHIKEAIKALKKLQ
>1A-16391
MPLKLGDQIPDFSLSDQLGVSRTNKQAKGRPLVLFFYPKDDTPGCTAENCGFRDKYDLFKLFGAEVWGVSGDD
EASHRSFADKNKLPFPLLCDTENSLRRAFGVPKVLGLLDGRVTYIIDSKGVIRHIFNDLLNGPAHVNEALRVLGEIR
N
>1A-01441
MTLCIGDSAPDFTLPNQDGVDISLSSFQGSRVVIYFYPKDDTPGCTKEACSFRDNWELFKSNNIQVLGISKDASK
SHIKFIDKHKLPFTLLTDSDPCPVAASYESYGLKKFMGREYYGMMRHTFVVNKYGKIELIYLKVKSDNMANQILN
DLKLN
>1A-10331
MTTNECLRVGMQAPDFATTAVVDQEFKDITLSQYRGKYVVLFFYPLDFTFVCPTEITAFSDRYSDFSSKNTEVLG
VSVDSKFTHLAWIQTPRNEGGIGDINYPLVSDLKREICQSYNVLNEDGEADRGLFIINPSGIIMHSTINKAPVGRN
IDETLRVLQAYQYVESHPDEVCPAGWTPGDKTMKEDPKGSKEYFSAL
>1A-03631
MVRTASTMLPLGTQLPDFELGVVSGVNSAPDDPLKGLSHIRSVDLTKRPLFLMVICAHCPFVKHVESGITNLFNS
FGNDIQFLAISSNSLITHPQDAPEFLASQANKLGWKFPYLFDSDQKLAKALRAACTPDFYVFWPSPDGNLTLRY
RGQMDGSRPGNEIPVSGDDIRLVFKSLLKGEVISANQKPSIGCNIKWHPGMEPEWFG
>2A-0304
MKRSEFIKSFILFSSILFIKPSRLFASINSVKIGKKAPNFLLNGYNKNSPNKKQWSLDDFSKKWLVLYFYPKDFSSGC
TLEAKNFQDNLSKYNKINASIVGISADSEEEHESFCTSENLGYTLLSDANSVISKSYDSWLDPYSKRNTFLINPEGIV

VYKWIGVRPIGHAQEVLKELTKQQKIYA
>2A-0727
MKLKVGDQIPSFSLKDQKGNIRTSNNLKKSLVLFFYPKDDTPGCTIEACGFRDKYDLFKILGAEVWGISNGSTQS
HLGFANKNKLQYPLLCDQNNILRNQFGVPKKLGFIEGRVTYIINSEGVIKHIFEDLLNGPAHIKEAIKALKKLQ
>2A-0787
MPLKLGDQIPDFSLSDQLGVSRTNKQAKGRPLVLFFYPKDDTPGCTAENCGFRDKYDLFK
LFGAEVWGVSGDDEASHRSFADKNKLPFPLLCDTENSLRRAFGVPKVLGLLDGRVTYIID
SKGVIRHIFNDLLNGPAHVNEALRVLGEIRN
>2A-1443
MTLCIGDSAPDFTLPNQDGVNISLSSFQGSRVVIYFYPKDDTPGCTKEACSFRDNWELFKSNNIQVLGISKDASK
SHIKFIDKHKLPFTLLTDSDPCPVAASYESYGLKKFMGREYYGMMRHTFVVNKDGKIELIYLKVKSDNMANQILN
DLKLN
>2A-0352
MTTNECLRVGMQAPDFATTAVVDQEFKDITLSQYRGKYVVLFFYPLDFTFVCPTEITAFSDRYSDFSSKNTEVLG
VSVDSKFTHLAWIQTPRNEGGIGDINYPLVSDLKREICQSYNVLNEDGEADRGLFIINPSGIIMHSTINKAPVGRN
IDETLRVLQAYQYVESHPDEVCPAGWTPGDKTMKEDPKGSKEYFSAL
>2A-1649
MVRTASTMLPLGTQLPNFELGVVSGVNSAPDDPLKGLSHIRSVDLTKRPLFLMVICAHCPFVKHVESGITNLFNS
FGNDIQFLAISSNSLITHPQDAPEFLASQANKLGWKFPYLFDSDQKLAKALRAACTPDFYVFWPSPDGNLTLRY
RGQMDGSRPGNETPVSGDDIRLVFKSLLKGEVISANQKPSIGCNIKWHPGMEPEWFG
>6301-0883_d
MPVSRRQLLLSSLLALPALVLAPRSAQALGGPQPPVDEPAPDFSLPTDDGRERLSLADFRGQWLVLYFYPKDGTP
GCTLEAQRFQQDLAAYAERNAQIVGVSADDLSSHSRFRENEGLSYPLLADVKGEVSKRYGSWLAPFSLRHTYIID
PEGVLRANFTAVRPVIHSKEVLAKLDELQAG
>6301-2152_c
MAIAVGDVAPDFSLPAQDGTTVSLSDFRGQKPVVLYFYPKDDTPGCTIEACSFRDSYTAFQEVGAVVLGVSSDSI
DSHQRFAQKYNLPFQLLSDAGDRLRQTYGVPKTLFVIPGRVTYVIDKEGKVRHIFDSLLNAQAHIQESLNILRSL
>6301-2287_c
MPLQVGDRAPDFTLPDQQGNPVSLTDLRGQRVVIYFYPKDDTPGCTKEACGFRDDFSLFEQAGIVVLGVSKDP
ASKHQKFIAKYELPFTLLTDADAAVASAYDSYGLKKFMGREYMGMMRHTYVIDVEGKIEQIYTKVKPETHARQIL
TDLGVAVAE
>6301-1915_d
MALTVGTAAPDFTALDDAGQSIQLSQFRGKTIVLYFYPKDDTPGCTKEACSFRDSYSAYQGKDIIVLGVSTDDSSS
HEQFKTKFSLPFPLVADPDRSITQAYDVDGGGYAKRVTYVIDGEGQIIRVYDSVKTDTHAGDILADLGL
>6301-1793_d
MRLMTEGALRVGQLAPDFEATAVVDQEFQTIKLSNYRGKYVVLFFYPLDFTFVCPTEITAFSDRYADFSALNTEIL
GVSVDSQFSHLAWIQTSRKEGGLGDLAYPLVADLKKEISTAYNVLDPAEGIALRGLFIIDKEGVIQHATINNLAFGR
SVDETLRVLQAIQYVQSHPDEVCPANWQPGAATMNPDPVKSKEFFAAV
>6301-1657_c
MSLRLGDTVPNFTQQSSEGEINSYDWAGDSWVILFSHPADYTPVCTTELGTVAKLEEEFAKRNVKPIALSVDDV
ESHKGWIGDINETQGTQVNYPILADPDRKVSDLYDMIHPNANNTLTVRSVFIIDPNKKLRLTLTYPASAGRNFDE
LLRVIDSLQLTDNYSVATPANWNDGDDVVVVPSIPTEQAREQFPKGVTEVKPYLRLTPQPNR
>6301-2361_c
MAKTASTMLALGTAAPDFALPDVVSGRTVQLADFSDRAGLLVMFICQHCPFVKHVEEELAAIGRDYLDLGIVAIS

PNSLETHPQDGPEQLKAQAERLGFTFPYCLDETQAIAKAYTAACTPDFFLFDRDRRLVYRGQLDDSRPSSDRPVT
GADLRAAITAVLTGDAPSADQKPSIGCNIKWHPGQEPDYYGAQLV
>7942-0642
MPVSRRQLLLSSLLALPALVLAPRSAQALGGPQPPVDEPAPDFSLPTDDGRERLSLADFRGQWLVLYFYPKDGTP
GCTLEAQRFQQDLAAYAERNAQIVGVSADDLSSHSRFRENEGLSYPLLADVKGEVSKRYGSWLAPFSLRHTYIID
PEGVLRANFTAVRPVIHSKEVLAKLDELQAG
>7942-1942
MAIAVGDVAPDFSLPAQDGTTVSLSDFRGQKPVVLYFYPKDDTPGCTIEACSFRDSYTAFQEVGAVVLGVSSDSI
DSHQRFAQKYNLPFQLLSDAGDRLRQTYGVPKTLFVIPGRVTYVIDKEGKVRHIFDSLLNAQAHIQESLNILRSL
>7942-1806
MPLQVGDRAPDFTLPDQQGNPVSLTDLRGQRVVIYFYPKDDTPGCTKEACGFRDDFSLFEQAGIVVLGVSKDP
ASKHQKFIAKYELPFTLLTDADAAVASAYDSYGLKKFMGREYMGMMRHTYVIDVEGKIEQIYTKVKPETHARQIL
TDLGVAVAE
>7942-2180
MALTVGTAAPDFTALDDAGQSIQLSQFRGKTIVLYFYPKDDTPGCTKEACSFRDSYSAYQGKDIIVLGVSTDDSSS
HEQFKTKFSLPFPLVADPDRSITQAYDVDGGGYAKRVTYVIDGEGQIIRVYDSVKTDTHAGDILADLGL
>7942-2309
MTEGALRVGQLAPDFEATAVVDQEFQTIKLSNYRGKYVVLFFYPLDFTFVCPTEITAFSDRYADFSALNTEILGVS
VDSQFSHLAWIQTSRKEGGLGDLAYPLVADLKKEISTAYNVLDPAEGIALRGLFIIDKEGVIQHATINNLAFGRSVD
ETLRVLQAIQYVQSHPDEVCPANWQPGAATMNPDPVKSKEFFAAV
>7942-2449
MSLRLGDTVPNFTQQSSEGEINFYDWAGDSWVILFSHPADYTPVCTTELGTVAKLKEEFAKRNVKPIALSVDDV
ESHKGWIGDINETQGTQVNYPILADPDRKVSDLYDMIHPNANNTLTVRSVFIIDPNKKLRLTLTYPASAGRNFDE
LLRVIDSLQLTDNYSVATPANWNDGDDVVVVPSIPTEQAREQFPKGVTEVKPYLRLTPQPNR
>7942-1730
MMAKTASTMLALGTAAPDFALPDVVSGRTVQLADFSDRAGLLVMFICQHCPFVKHVEEELAAIGRDYLDLGIV
AISPNSLETHPQDGPEQLKAQAERLGFTFPYCLDETQAIAKAYTAACTPDFFLFDRDRRLVYRGQLDDSRPSSDR
PVTGADLRAAITAVLTGDAPSADQKPSIGCNIKWHPGQEPDYYGAQLV
>9311-1400
MNRRSLLQTAVLGAGIFLLAPTRVHALGGQSPEIGIKAPDFDLPGFSSVNPDQKHWSLTGLQGRWLVVYFYPRD
FTSGCTIEAHGFQDSLSAFNKSGAEVVAISADSVSDHESFCSSEELKFPLLSDPDGLVSKAYGSWMAPYSMRHTF
LIDPESVVQAVWTGVRPVGHAKEVLNRLNDLQTS
>9311-2508
MTLQIGDLAPDFTLPDQNGEPVHLASLRGKRVVIYFYPKDATPGCTKEACNFRDRWSSFKDHGIHVLGISKDNA
SSHTRFIAKQELPFTLLSDEDPCPVASSFESYGLKKFMGRESMGMMRHTFVVDAEGRLELIYRKVKSDSMADQI
LSDLGIS
>9311-1556
MALTIGDRAPEIALEDQDGVLRRRDELQDKVLVLFFYPKDDTPGCTAEACAFRDTYATFEALGAVVWGVSSDDA
VSHRKFAQRYQLPFPLLSDQGQRLRTSFGVPKVLGLLPGRVTYVIDAEGTIRHLFNNMLDGPAHVREAERIVTEL
SKG
>9311-1322
MGQQAPDFTATAVVDQEFQEISLSQYRGKYVVLFFYPLDFTFVCPTEITAFSDRYSDFSSKNTEVLGISVDSQFSH
LAWIQTARNQGGIGDINYPLVSDLKKEISTAYNVLDDAEGVALRGLFIIDPEGVIMHATINNLPVGRNVDETLRVL
QAFQYVQSNPDEVCPANWTPGEKTMKPDPKGSKEFFAAIG

>9311-0254
MLDAMVLTPSTMLPLQHPLPQFSLPLVSGHPPWSSLDELVTSRDLPGRPLLVMLICAHCPFVKHVEPEITRVESD
FAGQITLLAISSNSLTTHPQDGRDGLRQQADQQGWRFPYLLDEQQTLAKDLRGACTPEFYAFAPDADGTQTLR
YRGQLDSSRPGNNQPLDGADLRAALTNILAGT
AVSETQLPSVGCNIKWNPGQEPPWFGQST
>9311-0689
MIQMKSFDQVPDVVFHTRVRDESVGGTNPFVWKDRSSAEIFHNKKVVLFSLPGAFTPTCS
SNHLPRYEELYDEFRSQGVDQVICLSVNDAFVMFQWGKHIGVKNIFLLPDGNGEFTRKMG
MLVSKDNLGFGMRSWRYSMLVDNGTIEKIFIEPGFADCCPDDPFEVSDADTMIAYLKGSTSSGIAKPNDFVG
>9605-1406
MNRRELLVKSGLFLAALTLTPSRASALGGVVLETGTTVPDFDLPGSSQSEPDRKQWSSRDLRGRWLAAYFYPRD
FTGGCTIEARGFESLHNDFLQAGAEVIGISADSVDDHESFCESEGLSFPLLSDPDGTVSKAYGSWMAPYSLRHTF
LIDPDGVLRERWVAVRPNGHAREVLDSLVTFQSEAAV
>9605-1141
MALGIGDRLPSFSLEDQNGDLRTPASVQGRWLVLFFYPKDDTPGCTAEACSFRDNAESFAALDAEVWGISGDD
AVSHRRFATRHNLTFPLLCDRNNALRREMGVPKALGLLPGRVTYIVDGEGVIRHTFSNLLDGPAHVREAQQVLN
QLRG
>9605-2307
MSLQIGDAAPDFTLPDQNGDSISLASLRGKKVLLYFYPKDDTPGCTKEACNFRDRWEQLKANNITVLGISKDGA
TSHNKFINKHELPFTLLTDEEPCAVASLYESYGLKKFMGREYMGMMRHTFLIDEEGKLERIYLKVKAATMADTLIS
DLGLS
>9605-1324
MTDTGCLRVGQLAPDFTATAVVDQEFKEVTLSQYRGKYVVLFFYPLDFTFVCPTEITAFSDRYADFSSKNTEVLGV
SVDSQFSHLAWIQTPRNQGGLGDINYPLVADLKKEISTAYNVLDDAEGVALRGLFIIDPDGVIMHATINNLPVGR
NVDETLRVLQAFQYVQSNPDEVCPANWTPGEKTMKPDPKGSKEYFSAIG
>9605-0215
MALTPSTMLALGTRLPDFDLPQVTDGRLNSQSLVERPVLLMVLCAHCPFVKHVEPELSRLDHDFSEAVQLVGVS
SNSLITHPQDGPEQLADQAKRQGWSFPYLLDEQQTLARDLRAACTPEFYLFSPDGEGLQSLRYRGQLDGSRPS
NDLPLDGRELRAALDAVLTGSPVNPDQTASVGCNVKWNPGHEPEWFG
>9902-1080
MHRRELLIQTGLLLTAWSLKPSRASALGGVVLETGNTAPAFSLPGSSRSQPDQSNWSLQDFKGRWVAVYFYPR
DFTGGCTIEARGFETLHSDYLAANAEVVGISADSVDDHESFCESEGLSFPLLSDPDGTVSKAYGSWMAPYSLRHT
FLIDPDGVLQQRWVAVRPSGHAQEVLDFLTNLQNKTSV
>9902-1316
MGLGIGDRLPNFSVEDQNGDQRSSESVQGRWLVLFFYPKDDTPGCTAEACSFRDNSSNFAALNAEVWGVSG
DDAVSHRRFAERHSLSFPLLCDRNNALRRQMGVPKALGMLPGRVTYVVDTTGVIRHTFSNLLDGPAHVREAER
VLKELQS
>9902-0386
MALQVGDPAPDFTLPNQDGESVQLSSLKGKRVILYFYPKDDTPGCTKEACNFRDRWGRFEEHNIKVFGISKDN
AASHTKFISKHSLPFTLLTDEEPCAVASLYNSYGLKKFMGREYMGMMRHTVVIDAEGRIERLYLKVKAATMADTL
LSDLGLD
>9902-1150
MTIMTDNGCLRVGQKAPDFTATAVVDQEFKEISLSQYKGKYVVLFFYPLDFTFVCPTEITAFSDRYSDFSSKNTEV
LGVSVDSQFSHLSWIQTARNQGGLGDINYPLVSDLKKEIATAYNVLDDAEGVALRGLFIIDPDGVIMHSTINNLPV

GRNVDETLRVLQAFQYVQANPDEVCPANWTPGEKTMKPDPEGSKEYFSAIS
>9902-0242
MPSGAILMETVWICSAAMARTASTMLALGTPLPAFRLARVNGDKVNGVQVKADTFASTDFEQKPILLMVLCA
HCPFVKHIEPEITRLENDFASQVQFVGLSSNSLITHPQDGPAQLAEQAQRHGWAFPYLLDDQQVLAKSLQAAC
TPEFYLFSQDSKDSSPTLQYRGQLDSSRPGNNQPLNGSDLRAALNAVLTGTSVSQKQVASVGCNVKWNPGQE
PEWFG
>JA23-2186
MGLVVGLLVGFGMTQPAWAQWGTTPLPPIGSPAPEFALPDQSGQIRRLADFRGEWVVLYFYPRDFTSGCTIEA
RRFQQDLPKFRAMGAQVVGVSADSVDSHRRFCSAEGLQFPLLSDPDGTVSRAYGSWMGDVALRNTFLIDPEG
ILREIDPIVNPSRHSAEVLAQLQQLIRN
>JA23-0523
MPLAVGDPAPEFTLPDAEGNPISLSQLRGQRVVLYFYPRDNTPGCTQEACGFRDAYADYQAHGIAILGVSADDA
RSHQKFAQKLQLPFPLLVDEGAKVARAYGVYGPKKFMGKEYNGIHRTTFVIDPEGKIEAVITKVKVETHAAELLK
QLTGS
>JA23-1057
MVLAVGTPAPGFNTTDTQGNQVSLADFAGKTVVLYFYPKDDTPGCTKEACGFRDAYADYQSKDVVVLGVSMD
DQASHQKFTEKYNLPFPLLVDSSGVITRAYDVDGGGYAKRVTYVIDGEGKIAKVYQNINTETHARDILADLGL
>JA23-1376
MGIVIGAVAPDFSLPAVGKGIQKGAQISLAAYRGKSKVLLAFYPADFSPVCTSEMRCFREDWEAFRAAGCEILGIS
SDPLSRHQAFAEQLKLEFPLLSDVDRTVSQQYGVDSLLGTRRAYFLVDSQGILRYQHVEWLPLFKRDNRELLEAV
RAIS
>JA23-2254
MSQEGCLRVGQPAPDFSATAVYDMEFKTVKLSDYRGKKYVVLFFYPLDFTFVCPTEITAFSDRYDDFAKLDTEILG
VSVDSEYSHLAWIQTDRKAGGVGELRYPLVSDLKKEISAAYNVLDPEAGVALRGLFIIDKEGIIQHATINNLAFGRS
VDETLRTLQAIQYVQSHPDEVCPANWQPGQKTMHPDPVKSKEFFAAIAK
>JA23-0113
MSLRLGDTAPNFTQKSTHGEIDFYSWAGDSWVVLFSHPADYTPVCTTELGTVAKLMPEFEKRGVKVIALSVDD
VDSHVGWVKDIEETQNATLTYPILADADRKVSQLYGMLDQTNLNQEGLPLTVRSVFVIDPNKKIRLILTYPASTGR
NFDELLRVIDALQLTDKYNVVTPADWKDGDDVIIPPSLKDPEVLKEKFPKGYKEIKPYLRVAPQPNK
>JA23-0140
MTSNSGLFNRRFADNFIPQPANTSLEIGQIAPDFELPRVQGDPVRLSDYRGQKPVVLAFTRIFTEKLFCPFCYPHI
QELKARYGEIQALGAELLMVTSTDPVQSQQIQADLQLPYPLLVDPECKTFRLYGVGQALGAPLPAQFVVDREGK
LRFKHVFSFAQPNADTDEVLRVLREGLQTGWDGTRRVAPIPFF
>JA23-2461
MIEETLRRRFVMARTPSTMLPLGTPAPDFSLPDVVSGQTISLSTFADKPALLVMFICQHCPYVKHVQEELARLGR
DYKDRGVGIVAISANDVEKYPDDSPENLKKMAETLGFTFPFCYDETQEVAKAFTAACTPDFFLFDADRKLVYRG
QLDDSRPSLTDVPVTGKDLRAALDNLLAGKPIDPDQKPSLGCNIKWKPGNEPAYFAG
>JA33-0907
MGLVGLFTALLVSWAGVQPALAQWGTTPLPQIGAPAPEFELPDQSGQVRRLADFRGKWVVLYFYPRDFTAGC
TIEARRFQQDLPKFRTLGAEIVGVSADSVDSHRRFCSAEGLQFPLLSDPEGTVSRAYGSWTGEMALRNTFLIDPE
GILRAIDPIVNPSRHSAEVLAQLQELAQAG
>JA33-2145
MALAVGDPAPEFTLPDAEGNLVSLSQLRGRRVVLYFYPRDNTPGCTREACGFRDAYAEYQAHGIHVLGVSADD
ARSHQKFAQKLQLPFPLLVDEGAKVASAYGVYGPKKFMGKVYNGIHRTTFVIDPEGKIEAIITKVKVEAHAAELLK

QLTGS
>JA33-0672
MVLAVGTVAPGFNTTDTHGNQVSLADFTGKTVVLYFYPKDDTPGCTKEACGFRDAYADYQSKNVVVLGVSMD
DQASHQKFAEKYNLPFPLLVDTSGVIARAYDVDGGGYAKRVTYVIDAEGKIAKVYQNINTETHARDILADLGL
>JA33-2305
MGIVIGTAAPDFNLPAVGKGIPKGATISLGAYRGKSKVLLAFYPADFSPVCTSEMRCFREDWGAFRAAGCEILGIS
SDPLSRHEAFAEQLKLEFPLLSDVDRKVSQLYGVDSFLGTRRAYFVVDSQGILRYQHVEWLPIFKRDNRELLEAVR
AVG
>JA33-0537
MSQEGCLRVGQPAPDFSATAVYDMEFKTVRLSDYRGKKYVVLFFYPLDFTFVCPTEITAFSDRYDEFAKLDTEILG
VSVDSEYSHLAWIQTDRKAGGVGELRYPLVSDLKKEISAAYNVLDPAAGVALRGLFIIDKEGIIQHATINNLAFGRS
VDETLRTLQAIQYVQAHPDEVCPANWQPGQRTLNPDPVKSKEFFAAVAK
>JA33-2849
MALRLGDTAPNFTQKSTHGEIDFYSWAGDSWVVLFSHPADYTPVCTTELGTVAKLMPEFEKRGVKVLALSVDD
VDSHLGWVKDIEETQNAIVNYPILADVDRKVSQLYGMLDQTNLNQEGLPLTVRSVFVIDPNKKIRLILTYPASTGR
NFDELLRAIDALQLTDKYSVVTPADWKDGDDVIIPPSLQDPEVLKEKFPKGYKELKPYLRLTPQPNK
>JA33-1949
MTSNSSLFNRRFFDNFIPQPANSPLQIGELAPDFELPNVHGDPVRLSDYRGQKPVVLAFTRIFTEKLFCPFCYPHI
QELKARYEEIRALGAELLMVTSTDPVQSQQIQADLELPYPLLVDPECKTFRLYGVGQALGAPLPGQFIVDREGRL
RYKHLFSFAEPNASTDEVLRVLREGLASGWDGTRRVAPVPFF
>JA33-2781
MARTPSTMLPLGTPAPDFSLPDVVSGQTISLSTFADKPALLVMFICQHCPYVKHVQEELARIGRDYKDRGVGIVA
ISANDVEKYPDDSPENLKKMAETLGFTFPFCYDESQEVAKAFTAACTPDFFLFDAERKLVYRGQLDDSRPSLPDV
PVTGKDLRAALDNLLAGKPIDPDQKPSLGCNIKWKPGNEPDYFKQA
>7002-A0109
MLQFFRTILITVVAIAFMWFPGEAAIALGGPQPELNQLAPEFTLLGNDGEGKIQEFSLQDYRGQWVVLYFYPQD
FTPGCTLEARRFQQDLPKYLERNVQVLGVSVDDVDSHEAFCDAEGLKFPLLADSTGDVSKQYGSYLTGYSLRHTY
LIDPEGILRKIYLGVNPAIHSQEVLTDLDSLMANSSAQLPPMG
>7002-A1108
MTTLTIGQAAPDFALTNAQGEARTLADYSGKWLVFYFYPKDNTPGCTTEALDFTALVPEFTALNAVIVGVSPDSE
KSHGRFIEKKELAIELLSDPDHQTAGVYGVWQLKKFMGKEYMGIVRSTFLIDPQGQVAAIWSKVKVKGHAQTV
LETLQT
>7002-A0558
MAAQVGQLAPDFTATAVIDQEFKTIKLSDYRGQYVVLFFYPLDFTFVCPTEVGAFSDRHGEFQKLNTEVLGVSV
DSEFAHLAWIQTDRKMGGVGDLAFPLVSDLNKTISTAYGVLEPEAGISLRGLFIIDPEGMIQHITVNNFSFGRSLD
ETLRVLQAIQHVQTNHNEVCPVDWQVGDRTMVPNPSEAQAYFSTL
>7002-A0320
MSLRLGDVAPNFTQDSTIGEINFYDWAGDSWVVLFSHPADFTPVCTTELGEVARLKGEFEKRNAKVIALSVDDT
ASHNGWVGDIDETQGCAVNYPILSDADQKVATLYNMIHPNANPKVTVRTVFVIDTERKVRLTITYPPSTGRNFQ
EILRVLDSLQLTDNYSVATPVNWQDGEDVVVSPAISTEDAKVKFPKGVKEIKPYLRMTPQPNK
>7002-A1383
MARTESTMLPLGAPAPDFALPDVTTGKTVSLGDFRGKAGLLVIFMCRHCPFVIHVQDELARLGVDFVPQDLGIV
AISANDAENYPDDGPEKLKEMAAALQLNFPILYDESQAIAKSYQAACTPDFFLFDQDLKLVYRGQLDDARPGND
KPVTGADLRQAIAAVLAKETPDPNQKPSLGCNIKWKPGNAPSYFG

>7002-A0793
MARVPDVVFKTRVRDESVEGPNPYRWEDKTTADIFGGKKVVLFSLPGAFTPTCSSNHLPRYEELYSEFQAQGV
DEIICLSVNDAFVMFKWGKEIGADKVFLLPDGNGEFTRKMGMLVEKSNLGFGMRSWRYSMLVEDGEIKKMF
VEPDFSDNCPTDPFEVSDADTMLAYIKGESAPGVSEPRKEFVG
>307-1384
MRRQEVLKLLGALPLLALRPRPAAAMGGTLPALDQLAPDFDLESAGQTGALGQRLQRDDFRGSWLVLYFYPRD
FTSGCTLEARGFQRDLSLYKAANGAVVGVSADGSDKHASFCTSEGLDFPLLSDPGGEVSKLYGSWIPPFSQRHTF
VIDPDGVLRARFVGVNPSIHSQEVLSTLKELQV
>307-0392
MALQIGDPAPDFTLPDQDGQPVALKDLRGQRVVIYFYPKDDTPGCTKEACNFRDQWDSFKSHGIKVLGISKDN
AKSHAKFIDKYTLPFTLLSDSEPCPVAESYESYGLKKFMGREYMGMMRHTFVIDADGKLEKIYKKVKAATMADD
ILKDLELAG
>307-1342
MPIAVGDQAPPFKAKDQNGVERCLADLGGKPLVLFFYPKDDTPGCTIEACGFRDQFAALQALGAEVWGVSGD
GAGSHRRFVSKFQLPYPLLVDEGNALRDAFGVAKTLFVIPGRVTYVIDGGGVVRLVFDEMLDANAHVRQAMA
TLQQLKG
>307-1238
MTTGVFRVGLQAPDFTATAVVDQEFKDISLSQYRGKYVVLFFYPLDFTFVCPTEITAFSDRYDAFKALNTEVLGVS
VDSQFSHLAWIQTERKQGGLGDIAYPLVADLKKEIASAYNVLDEAEGVALRGLFIINPEGVVQHATVNNLPVGRN
VEETLRVLQAFQHVEANPDEVCPANWTPGERTMNPDPVGSKDFFAAVN
>307-0386
MCHDQKDTLSTVMALQLGDTVPNFTQDSQQGPINFYDYAGDSWVVLFSHPADYTPVCTTELGAVAKLRAEW
DKRNVKTIALSVDSAESHKGWIGDINETQCTVVDYPILADEDKTVSDLYGMIHPKALNNLTVRSVFVIDPAKKLRL
QITYPASTGRNFDEILRVIDSLQLTDHHQVATPVNWKDGEDCVVVPSIPTDEARNKFPKGVTEVRPYLRMTPQP
NR
>307-2285
MALTASMMLPLGTPLPPFSLPTVEGGTWSTQQLDRRPLLVLFICAHCPYVKHVEASLSAMAQDFSAELQMLAIS
SNSVITHPQDAPEHLLAQKLRCGWDFPYLHDVDQQVARLFQAACTPDPYLFDGDQRLIYRGQLDASRPSSGQ
ASDCADLRTALAAWKAGQPPLLQQHPAMGCNIKWQP
>7803-1236
MNRRQLLQSGLVAAAAITLRPGKTWALGGVAPEIGSSAPDFELAGTNLNDPEKNRWTLSDWSGRWLVLYFYP
RDFTSGCTIEAHGFQDTLTEFQKLNCEITAISADSVEDHESFCNSEDLGFTLLSDHDGVVSRSYGSWMAPYSLRH
TFLIDPSGVVQARWTGVRPVGHAQEVLEALKGLQTS
>7803-1042
MALKTGDAAPSFALEDQDGQMRRLEDLKGKILVLFFYPKDDTPGCTAEACTFRDQYSDLTALGAEVWGVSGDD
AVSHRRFAERHQLPFPLLSDNTGNLRRSFGVPKTFGLLPARVTYVIDQQGVIQLVFNNLLDGPAHVKEAEAVVRT
LAAG
>7803-2172
MTLQIGDPAPDFTLPNEKEELVTLSSLKGQRVVIYFYPKDATPGCTKEACNFRDRWDQFEAHGIRVLGISKDNAA
SHSRFISKQELPFTLLTDVEPCAVASAYESYGLKKFMGREYMGMMRHTFVVDAQGNLELIYRKVKAEIMADQIL
NDLGLTAS
>7803-1118
MTANGCLRVGQQAPDFTATAVVDQEFKEISLSQYRGKYVVLFFYPLDFTFVCPTEITAFSDRYADFSSKNTEVLGV
SVDSQFSHLAWIQTPRNQGGLGDINYPLVADLKKEIASAYNVLDDEEGVALRGLFIIDPEGVIMHATINNLPVGR

NVDETLRVLQAFQYVQSHPDEVCPANWTPGEKTMKPDPVGSKEFFAAVN
>8102-1280
MRRRDLFIKSGLLLTALSITPTKAWSLGGVVLEQGAEVPSFDLPGSSRREPDRDRWSSDDLRGRWLAVYFYPRD
FTGGCTIEARGFESLHSDFLAAGAEVVGISADSVDDHASFCESEGLSFPLLSDPDGAVSKAYGSWMAPYSLRHTF
LIDPSGILRQRWVAVRPSGHAQEVLDAITDLQNNTSI
>8102-1015
MAIGVGDSLPSFCLDDQDGVQRTPETARGRWLVLFFYPKDDTPGCTAEACGFRDSSAAFQELGAEVWGISGD
DAISHRRFATRHGLNFPLLVDRNNSLRRSLGVPKALGLVPGRVTYVVDGEGVIRHVFSNLLDGPAHVREAERVIS
SLQG
>8102-2162
MALQIGDPAPDFSLPNQDGDLVQLSSLRGQRVVIYFYPKDDTPGCTKEACNFRDRWDRFEQHGIKVLGISKDN
ATSHTKFISKHELPFTLLTDVEPCEVASSYESYGLKKFMGREYMGMMRHTFVVDADGKLERIYLKVKSATMADTI
LSDLELA
>8102-1213
MTETGCLRVGQQAPDFTATAVVDQEFKEISLSQYRGKYVVLFFYPLDFTFVCPTEITAFSDRYADFSSKNTEVLGV
SVDSQFSHLAWIQTPRNQGGLGDINYPLVADLKKEIATAYNVLDDAEGVALRGLFIIDPDGVIMHSTINNLPVGR
NVDETLRVLQAFQYVQSNPDEVCPANWTPGEKTMKPDPKGSKEYFSAIG
>8102-0221
MALTPSTMLELGTPLPAFSLPVVAGGSISSADLHGRPLLLMVICAHCPFVKHVEPEVSRLEQDYGTAVQLLAVSS
NSLITHPQDGPKQLADQAKRWGWSFPYLLDEQQSLAKALRAACTPEFYLFDADANGLQTLLYRGQLDGSRPG
NDVPLNGVDLRAALDAMLAGQAVNPEQRPSVGCNVKWNPGQEPDWFG
>6803-0221
MTSKKFSWPKTIIALLLTLGLWLGLADLPTYALGGIQPELDQPAPLFTLPSTTGEGEVNLTDYRGQWVVLYFYPQ
DFTPGCTLEAQRFQRDLTKYQALNAQVIGVSVDDLDSHEAFCDAEGLKFPLLADSDGAVIKTYGSWLSGMALR
HTYVIDPEGILRERFLGVRPATHSEEVLARLAELQA
>6803-0242
MATALETNQPAPTFSAPNAEGKTISSDDFLGQWLVLYFYPKDNTPGCTTEAIDFSEKLPEFTDLNAVVVGVSPDS
EKSHGKFIDKHNLTVQLLSDPEHELAAAYGAWGPKKFMGKECEGILRSTFLINPQGNIAHIWPNVRVKGHAEKV
LEKLQQLNSAD
>6803-0755
MTEVLRVGQPAPDFTATAIVDQSFQTVKLSTYRGKYLVLFFYPLDFTFVCPTEIIAFSDRHSEFTALDTEVVGISVDS
EFSHLAWIQTERKMGGIGNINYPLVSDLKKEISQAYNVLEPDAGIALRGLFIIDREGILQYATVNNLSFGRSVDETL
RVLKAIRHVQSHPNEVCPVDWQEGDKTMIPDPEKAKTYFETVAEP
>6803-1198
MALQLGDVVPDFTQESSQGPISFHEWAGDSWVVLFSHPADYTPVCTTELGTVAKLKPEFDKRNVKVIALSVDD
VESHKGWICDIDETQNTTVNYPILADGDKKVSDLYGMIHPNALNNLTVRSVFIIDPAKKLRLTFTYPASTGRNFDE
ILRVIDSLQLTDYHQVATPANWQDGDKCVVVPSISTEDAKVKFPKGVEEIKPYLRLTPQPNK
>6803-1159
MNLQIELYKFQQESLKRSSPERAAIFSDFIQGLSEEFRNRRLLRIGDFAPDFTLKNTKGETIILSEQLKTGPILLKFFR
GYWCPYCGLELRAYQKVVNKIRALGGTILAISPQTLVASQKTIDRHDLTYDLLSDSGFQTAQDYGLVFTVPDAVKQ
IYLQSGCVIPEHNGTEEWLLPVPATFVIDRRGHIALAYANVDFRVRYEPEDAIAILLSLFVGN
>6803-1621
MTPERVPSVVFKTRVRDESVPGPNPYRWEDKTTEQIFGGKKVVLFSLPGAFTPTCSSNHLPRYEQLFEEFQALG
VDDIICLSVNDAFVMFQWGKQIGADKVKLLPDGNGEFTRKMGMLVEKSNLGFGMRSWRYSMFVNDGKIEK

MFIEPEFGDNCPVDPFECSDADTMLAYLKGAEAPGVSEPVKAFVG
>BP1-1194
MFLRSVLVALLSLILFLSPSAPSWALGGELPPLNAPAPDFSLPSSVNGQLISLKDYRGKWVVLYFYPKDFTSGCTLE
AQRFQRDIEQFHAHNAEVIGVSADSVDSHQDFCDSEGLTFPLLSDPDGQVSKAYGSWLGFVSLRHSFIIDPEGIL
RERYRKVNPAIHSQEVLARLEELQQQG
>BP1-1451
MSLTAGAIAPPFELADATGQLVRLADFQGQWVILYFYPRDNTPGCTTEACAYRELSATLGDRNVVILGISPDSVTS
HAKFQQKLDLPFRLLADVDAKVAQAYGSYGPKKFMGKEYLGVYRDTFLIDPTGKIAAIYRRVKPAAHVAQVLAD
LERLQG
>BP1-1198
MALAVGTPAPPFTAKDTQGNTISLSDFAGKTVVLYFYPKDDTPGCTKEACSFRDNYAAYQGKNIVVLGVSADDE
TSHQKFTAKFNLPFPLLADVDRSIIKAYDVDGGGYAKRVTYVIDGNGIISHVYTSVKTDTHATDILADLGL
>BP1-1454
MSECLRVGQPAPDFEAVAVYDQEFKTIKLSDYRGKYVVLFFYPLDFTFVCPTEIVAFSDRYDEFAKLNTEILGVSVD
SQFSHLAWTQTDRKAGGVGDLKYPLVSDLKKDISTAYNVLTEEGVALRGLFIIDKEGIIQHATINNLAFGRSVDETL
RVLQAIQYVQTHPDEVCPAGWHPGDKTMNPDPVKSKVYFEAVG
>BP1-2261
MSLKLGDVVPNFTQASSMGNINFYEWAGDSWVVLFSHPADYTPVCTTELGEVARLRPEFEKRNVKVLALSVDS
VESHLGWIKDIEEVNNVKVDYPILADEDKKVSTLYDMIHPNSLNNLTVRTVFIIDPQKRLRLTMTYPASTGRNFAE
ILRVIDSLQLTDNYSVATPANWQEGQECVIVPSLSDEEAKQKFPKGFNAVKPYLRLTPQPNK
>BP1-1289
MAVQLPFLTSTNFSGLFNERFWQNAWPLPPQNELKRGALVPDVALPGVGLSDRVRLSNEWKKQPLLLVFTRIFT
AHQYCPLCYPYLKTLNENHETFQGKGVAVLVVTSTDAQQSEKVKADMALKMPLLYDPSCQVFRKYRTGQALGA
PLPAQFLIDQEGKLHYKHLFSFLEPNAPLERLFQEIDALAQGATVTTAA
>BP1-1788
MARTESTMLALGTVAPDFQLPDVVSGQTISLSTFADKKALLVMFICRHCPYVKHVQQELAKLGRDYKDTGLGIV
AISANDAANYPEDAPESLKAMATELGFTFPLCYDESQETAKAYTAACTPDFFLFDSDRKLVYRGQLDDSRPQNGL
PVTGKDLRAAIDAVLAGQTPSEDQKPSLGCNIKWKPGNEPAYYR
>101-2703
MRSRRHFQTIFLVICLALITWLNFIPNAWALGGKLPELDQPAPEFTLPTNIGDREISLSDYRGQWVVLYFYPKDFT
SGCTIEARRFQQDLPKYRERNTQILGVSADDINSHQEFCDSEGLKFPLLADTDGTVSKAYGSWLNFFSMRHTFII
NPGGILQEIFLGVKPTIHSQEVLSSLDNLQQT
>101-3959
MALSVGTKAPEFTVKDTNGKTVSLSDFAGKTVVMYFYPKDDTPGCTKEAQSFRDNYAEYQGKDMVVLGVSM
DDEASHKAFTEKYGLPFQLLADVDGAITKAYDVEGGGYPKRVTYIIDAEGNISHVDQNVKTGSHAQDILEVIGKK
>101-3182
MKIQVGDQAPEFTLPSQEGTEVSLSDFRGKKNVVLYFYPKDDTPGCKIQACTFRDQYTIFKNVDAEIIGISGDSK
QSHKQFSTKYSLPFTLLCDTDNKVRQLYDVPPTLWILPGRATYVIDKEGIIRYICDSTFDFEVHSKEALKALRKLRVT
V
>101-0235
MTDECLRVGQKAPDFTATAVVDQEFETIKLSDYRGKYVVLFFYPLDFTFVCPTEITAFSD
RYKEFELLNTEILGVSVDSEFSHLAWIQTDRKSGGIGDLNYPLLSDIKKEISTAYNVLDL
EAGIALRGLFIIDREGIIQHATVNNFAFGRNVDEAIRTLQAIQYVQIHPNEVCPVGWKPG
DKTMNSDPIKSKEFFAAVS

>101-5038
MTLRLGDKVPDFTQDSSEGQISFYDWAGDSWVVLFSHPADFTPVCTTELGAVARLKPEFDKRGVKVIALSVDD
AESHKGWVGDINETQKTTVNYPILADVDKKVSDLYDMIHPNSLNNLTIRSVFIIDSEKKLRLTFTYPASTGRNFNEI
LRVIDSLQLTDNHQVATPVDWKDGDDCVIVPSLKDPEVLKEKFPKGYTEVKPYLRMTPQPNK
>101-0234
MLTSYDFRGLLNQRFFQNLLPIPAINDLDNGKRTPDFELPDITNGKLVRLSNYWGVTPVIVALTRIFTEKQYCPFCF
PHILALNKNYERFLERGVEVLMITSTDEKQSKIVVNDLSLKMPLLSDSSCQVFKNYKVGQALGAPLSGQFVLDKQ
GRLCYKHLFSFLDHNASIEDLLGILDNYIFE
>101-3842
MVLTESTMLKLGTIAPNFELPDVVSGNIISPSTFKGKKALLVMFICRHCPFVKHIQLELAKIGKDYVPKDVGIVAISS
NNIESHPDDAPDKLKEMASELEFNFPYCFDETQEVAKSYIAACTPDFFLFDVDYKLVYRGQLDDSRPSNEKPVTG
SDLRAALDTVLAGKNIEEEQKPSVGCNIKWKAGNEPSYFG
>101-0162
MVIDKVPDVVFKTRVRDESVDGPNPFRWQDKTTQEIFGGKRVVLFALPGAFTPTCSSTHLPRYEELYNEFKGQG
IDEIICLSVNDAFVMFQWGKQQGSKNVFLLPDGNGEFSRKMGMLVEKSNLGFGMRSWRYAMVVNDCTIEK
MFVEEGFADNYGDDPFEVSDADTVLAFLKGQEAPKDKPARLDFVG
>101-2011
MMILSVNEKTFKKEVLESSQPVLVYFWAPWCGLCKMIVPQLVKFQSEWNCHLKLVGVNADKSLKLASTYQLQT
LPTLILFVNGQIVNRLGYFQAREDFQRTLDAFISTLVKDPPSQPREHRNEKQSILW
